# *CNPY3* variants associated with a recessive developmental and epileptic encephalopathy affect inflammasome and TLR function

**DOI:** 10.1016/j.ebiom.2026.106506

**Published:** 2026-10-14

**Authors:** Vera Garloff, Mona Grimmel, Reza Maroofian, Stephanie Efthymiou, Sven Stengel, Dolores Bello, Carolin Tille, Mohamed Ghait, Oliver Ohlenschläger, Andrea Dieckmann, Heike de Vries, Matthias Waginger, Seba Nadeef, Ehsan Barkhordari, Rauan Kaiyrzhanov, Sarah Bühler, Irisa Pjeci, Stefanie Beck-Wödl, Javeria Raza Alvi, Tipu Sultan, Anna Chassevent, Constance Smith-Hicks, Irina Mishina, Agustí Rodríguez-Palmero, Evangeline Wassmer, Julie Vogt, Christopher Manuel Meudt, Rami Abou Jamra, Mohammad Reza Abbaszadegan, Naser Ajami, Reza Azizimalamiri, Hamid Galehdari, Niloofar Chamanrou, Mina Zamani, Gholamreza Shariati, Pardis Nourbakhsh, Farhan Bahadar Ali, Ambrin Fatima, Sohana Nadeem Hashmi, Zafar Iqbal, Sidra Kaleem, Mathias Toft, Erum Afzal, Shazia Maqbool, Fatima Rahman, Vincenzo Salpietro, Margot Grisval, Caroline Racine, Louis Januel, David Cheillan, Cecile Acquaviva-Bourdain, Chadi Al Alam, Majid Alfadhel, Muhammad Umair, Barbara Oehl-Jaschkowitz, Shahzad Haider, Vivek Jain, Bhuvandeep Narang, Fawzia Amer, Nour Elkhateeb, Carolina Alvarez, Eduardo Pérez-Palma, Loreto Ríos-Pohl, Rikke S. Møller, Debangana Baruah, Lokesh Lingappa, Ahmad Farooq, Maha S. Zaki, Gulnara Svyatova, Alexandra Murtazalieva, Asem Burabayeva, Kamran Salayev, Ulviyya Guliyeva, Kimia Kahrizi, Hossein Najmabadi, Anya Revah-Politi, Maureen S. Mulhern, Tristan Timothy Sands, Hafiz Muhammad Mustafa, Hani Akbar Rao, Gabriela Oprea, Aboulfazl Rad, Mohammad Miryounesi, Ali Mardi, Vykuntaraju K. Gowda, Varunvenkat M. Srinivasan, Rola Mohammed Alanazi, Iman Sabri Abumansour, Bader Alhaddad, Khadijah Bakur, Michael Bauer, Fowzan S. Alkuraya, Peter Huppke, Henry Houlden, Tobias B. Haack, Ignacio Rubio, Ralf A. Husain

**Affiliations:** a Department of Anaesthesiology and Intensive Care Medicine, Member of the Leibniz Center for Photonics in Infection Research (LPI), Jena University Hospital, Jena, 07747, Germany; b Institute of Medical Genetics and Applied Genomics, University of Tübingen, Tübingen, 72076, Germany; c Department of Neuromuscular Disorders, UCL Queen Square Institute of Neurology, London, WC1N 3BG, UK; d Department of Pediatric Neurology, Jena University Hospital, Jena, 07747, Germany; e Center for Sepsis Control and Care, Jena University Hospital, Jena, 07747, Germany; f Leibniz Institute on Aging - Fritz Lipmann Institute (FLI), Jena, 07745, Germany; g Section of Pediatric Radiology, Department of Radiology, Jena University Hospital, Jena, 07747, Germany; h Department of Translational Genomics, Centre for Genomic Medicine, King Faisal Specialist Hospital and Research Centre, Riyadh, 11564, Saudi Arabia; i Innovation and Research, King Faisal Specialist Hospital and Research Centre, Riyadh, 11211, Saudi Arabia; j South Kazakhstan Medical Academy, Shymkent, 160001, Kazakhstan; k Developmental and Behavioral Pediatrics, Children Hospital and the Institute of Child Health, 60000 Multan, Pakistan; l Department of Neurology and Developmental Medicine, Kennedy Krieger Institute, Baltimore, MD, 21205, USA; m Department of Neurology, Johns Hopkins University School of Medicine, Baltimore, MD, 21205, USA; n Research Centre for Medical Genetics, Moscow, 115522, Russian Federation; o Pediatric Neurology Unit, Department of Pediatrics, Hospital Universitari Germans Trias i Pujol, Universitat Autònoma de Barcelona, 08916, Badalona, Spain; p Department of Paediatric Neurology, Birmingham Women's and Children's Hospital, NHS Trust & Birmingham Health Partners, Birmingham, B4 6NH, UK; q Institute of Health and Neurodevelopment, Aston University, Birmingham, B4 7ET, UK; r West Midlands Regional Genetics Service, Birmingham Women's and Children's NHS Foundation Trust, Birmingham, B15 2TG, UK; s Department of Pediatrics, Varisano Klinikum Frankfurt Höchst, Frankfurt am Main, 65929, Germany; t Institute of Human Genetics, University of Leipzig Medical Center, Leipzig, 04103, Germany; u Faculty of Medicine, Department of Medical Genetics, Mashhad University of Medical Sciences, Mashhad, 9177948564, Iran; v Faculty of Medicine, Department of Basic Sciences, Islamic Azad University, Urmia Branch, Urmia, 6389657169, Iran; w Golestan Medical, Educational, and Research Center, Department of Pediatric Neurology, Ahvaz Jundishapur University of Medical Sciences, Ahvaz 6135733118, Iran; x Faculty of Science, Department of Biology, Shahid Chamran University of Ahvaz, Ahvaz 6155953916, Iran; y Narges Medical Genetics and Prenatal Diagnosis Laboratory, Kianpars, Ahvaz, 6155889467, Iran; z Faculty of Medicine, Department of Medical Genetics, Ahvaz Jundishapur University of Medical Sciences, Ahvaz, 6135733118, Iran; aa Department of Neurology, School of Medicine, Ahvaz Jundishapur University of Medical Sciences, Ahvaz, 6135733118, Iran; ab Department of Paediatrics & Child Health, The Aga Khan University, Karachi, 78400, Pakistan; ac Department of Biological and Biomedical Science, The Aga Khan University, Karachi, 78400, Pakistan; ad Centre for Regenerative Medicine and Stem Cells Research, The Aga Khan University, Karachi, 78400, Pakistan; ae Department of Neurology, Oslo University Hospital, Oslo, 0424, Norway; af Institute of Clinical Medicine, University of Oslo, Oslo, 0318, Norway; ag Department of Pediatric Neurology, Children's Hospital and Institute of Child Health, Lahore, 54600, Pakistan; ah Department of Developmental Behavioural Paediatrics, University of Child Health Sciences and The Children's Hospital, Lahore, 54600, Pakistan; ai European Brain Research Institute (EBRI), Rome, 00161, Italy; aj Service de pédiatrie, CHU Dijon-Bourgogne, Dijon, 21079, France; ak Centre de Génétique, FHU-TRANSLAD, CHU Dijon-Bourgogne, Dijon, 21079, France; al Genetics Department, Hospices Civils de Lyon, Bron, 69500, France; am Auragen Laboratory, Plan France Medecine Génomique, Lyon, 69003, France; an Biochemistry and Molecular Biology Laboratory, Inborn Errors of Metabolism Unit, Hospices Civils de Lyon, Bron, 69500, France; ao Carmen Laboratory - Inserm U1060, INRAE UMR1397, Pierre Benite, 60310, France; ap Pediatric Neurology, American Center for Psychiatry and Neurology, Abu Dhabi, United Arab Emirates; aq Medical Genomics Research Department, King Abdullah International Medical Research Center, King Saud Bin Abdulaziz University for Health Sciences, Ministry of National Guard Health Affairs, Riyadh 11426, Saudi Arabia; ar King Salman Center for Disability Research, Riyadh, 11614, Saudi Arabia; as Genetics and Precision Medicine Department, King Abdullah Specialized Children's Hospital, King Abdulaziz Medical City, Ministry of National Guard Health Affairs, Riyadh, 11426, Saudi Arabia; at Praxis für Humangenetik, Bioscientia MVZ Labor Saar GmbH, 66424, Homburg, Germany; au Shamshad Aslam Hospital, Wah Cantt, 47040, Pakistan; av Children's Neuroscience Centre, Neo Clinic Children Hospital, Jaipur, 302019, India; aw Revvity Omics Laboratory, Thane, Maharashtra, 400615, India; ax Department of Pediatrics, Pediatric Neurology and Metabolic Medicine Unit, Kasr Alainy Faculty of Medicine, Cairo University, Cairo, 12311, Egypt; ay Department of Pediatric Neurology, King Fahad Specialist Hospital, Dammam, 32253, Saudi Arabia; az Department of Clinical Genetics, Cambridge University Hospitals NHS Foundation Trust, Cambridge, CB2 0QQ, United Kingdom; ba Department of Pediatric Neurology, Advanced Epilepsy Center, Clinica Alemana, Santiago, 7550000, Chile; bb Hospital de Niños Dr. Luis Calvo Mackenna, Santiago, 7500539, Chile; bc Universidad del Desarrollo, Centro de Genética y Genómica, Facultad de Medicina Clínica Alemana, Santiago, 7610658, Chile; bd Clínica Integral de Epilepsia y Neurodesarrollo, Santiago, 7610448, Chile; be Department for Genetics and Personalized Medicine, Danish Epilepsy Centre, Dianalund, 5230, Denmark; bf Department of Regional Health Research, University of Southern Denmark, Odense, 5230, Denmark; bg Rainbow Children's Hospital, Hyderabad, Telangana, 500034, India; bh Department of Biochemistry, Women University Swabi, Swabi, Khyber Pakhtunkhwa, 23430, Pakistan; bi Clinical Genetics Department, National Research Centre, Human Genetics and Genome Research Institute, Cairo, 12311, Egypt; bj Center of Molecular Medicine, Almaty, 0500008, Kazakhstan; bk Fetal Neonatal Neuroimaging and Developmental Science Center, Division of Newborn Medicine, Boston Children's Hospital, Boston, MA, 02115, USA; bl MediClub Hospital, Baku, AZ 1010, Azerbaijan; bm Genetics Research Center, University of Social Welfare and Rehabilitation Sciences, Evin, Tehran, 1985713834, Iran; bn Institute for Genomic Medicine and Precision Genomics Laboratory, Columbia University Irving Medical Center, New York, NY, 10032, USA; bo Departments of Pathology & Cell Biology and Neurology, Vagelos College of Physicians and Surgeons, Columbia University Irving Medical Center, New York, NY, 10032, USA; bp Division of Child Neurology, Departments of Neurology and Pediatrics, Columbia University Vagelos College of Physicians and Surgeons and NewYork-Presbyterian Morgan Stanley Children's Hospital, New York, NY, 10032, USA; bq SMS Pakistan, Karachi, 75400, Pakistan; br VitroGenetics, Child Surgical Foundation, Clinical Genetics Department, Islamabad, 44000, Pakistan; bs Arcensus GmbH, Rostock, 18119, Germany; bt Genomic Research Center, Shahid Beheshti University of Medical Sciences, Tehran, 1985717413, Iran; bu Faculty of Medicine, Department of Medical Genetics, Shahid Beheshti University of Medical Sciences, Tehran, 1985717443, Iran; bv Center for Comprehensive Genetic Services, Shahid Beheshti University of Medical Sciences, Tehran, 1985717413, Iran; bw Department of Pediatric Neurology, Indira Gandhi Institute of Child Health, Bangalore, 560029, India; bx Department of Pediatrics, King Fahad Armed Forces Hospital, Jeddah, 23325, Saudi Arabia; by Faculty of Medicine, Department of Medical Genetics, Umm Al-Qura University, Makkah, 24381, Saudi Arabia; bz Lifera Omics, Riyadh, 13519, Saudi Arabia; ca College of Medicine, Alfaisal University, Riyadh, 11533, Saudi Arabia; cb Center for Rare Diseases, Jena University Hospital, Jena, 07747, Germany; cc Biochemistry and Molecular Biology Department, Hospices Civils de Lyon, Bron, 69500, France

**Keywords:** Neurodevelopmental, Epilepsy, Neuroinflammation, Innate immunity, Toll-like receptor, IL-1β, PRAT4A

## Abstract

**Background:**

Canopy FGF signalling regulator 3 (CNPY3) is an endoplasmic reticulum (ER)-resident chaperone essential for toll-like receptor (TLR) folding, trafficking and inflammasome activation. In rare paediatric cases, biallelic *CNPY3* variants have been associated with developmental and epileptic encephalopathy (DEE), but available data remain sparse.

**Methods:**

50 individuals from 38 unrelated families with biallelic *CNPY3* variants and DEE were evaluated for exome and genome findings, standardised clinical phenotype descriptions, EEG results, and neuroimaging data. A selection of variants underwent functional analysis focussing on protein stability and localisation, TLR2 function, NLPR3 inflammasome activation, and structural conformation.

**Findings:**

Disease onset occurred in early infancy (median age at onset 68 days), with a clinical phenotype comprising global developmental delay (100%) and intractable epilepsy (86%), accompanied by microcephaly (70%), spasticity (77%), growth faltering (63%), stridor (48%), and early mortality (38%). Epileptic spasms and tonic seizures (46% each) were the most frequent seizure types, and EEG findings included suppression-burst (37%) and hypsarrhythmia (28%). Phenotypic severity varied substantially, with most patients being severely affected (median score 16, range 2–20). Most variants occurred in the homozygous state (95%), including 13 missense changes and 7 truncating alleles. Variants were classified as of uncertain significance (71%), followed by likely pathogenic (23%) and pathogenic (6%) according to ACMG/AMP criteria. Genotype–phenotype correlation showed that individuals with truncating variants were, on average, more severely affected than those with non-truncating variants (median score 18 vs 14.5). Protein modelling predicted conformational effects for most missense variants. In cells, frameshift alleles abolished CNPY3 protein expression, whereas nonsense alleles markedly reduced protein levels. ER localisation of CNPY3 remained preserved across tested genotypes; however, capacity to support TLR2 maturation and processing varied substantially. Remarkably, some missense variants permitted TLR2 surface expression but impaired downstream signalling and inflammasome activation, uncoupling trafficking from signal transduction.

**Interpretation:**

CNPY3-associated DEE is a severe early-onset disorder with genotype-dependent phenotypic severity, accompanied by allele-specific disruption of innate immune pathways. Our findings reveal separable effects on TLR trafficking, downstream signalling and inflammasome activation, identifying CNPY3 as a key regulator of multiple cellular pathways involved in inflammatory responses and neurodevelopment.

**Funding:**

Interdisciplinary Center of Clinical Research and Jena School of Molecular Medicine, 10.13039/501100007653Jena University Hospital; 10.13039/100012957Friedrich Schiller University Jena; The Wellcome Trust; The Medical Research Council; 10.13039/100013128The MSA Trust; The National Institute for Health Research University College London Hospitals Biomedical Research Centre; 10.13039/100000864The Michael J Fox Foundation; 10.13039/501100000268Biotechnology and Biological Sciences Research Council; The Fidelity Trust; 10.13039/501100000833Rosetrees Trust; 10.13039/501100000346Ataxia UK; 10.13039/100013790Brain Research UK; Sparks GOSH Charity; Alzheimer's Research UK; CureDRPLA; UK Dementia Research Institute; 10.13039/501100000304Parkinson's UK; 10.13039/501100006095South-Eastern Norway Regional Health Authority, Norway; France Genomic Medicine Plan 2025; 10.13039/501100001659Deutsche Forschungsgemeinschaft; 10.13039/501100000780European Commission; Federal Government of Germany; State of Thuringia; 10.13039/100009374Pakistan Science Foundation; 10.13039/501100013302King Abdullah International Medical Research Center; 10.13039/501100003009Science and Technology Development Fund; 10.13039/501100019345King Salman Center for Disability Research.

**Research in context**

**Evidence before this study**

Biallelic variants in the gene *CNPY3* were first reported in 2018 in children with a severe condition that interferes with brain development and causes epilepsy. Since then, only a small number of affected children have been described worldwide. Studies in mice lacking CNPY3 show similar neurological features, supporting the idea that loss of *CNPY3* function is related to the condition. However, how changes in *CNPY3* lead to the observed clinical manifestations has remained unclear and limited the use of *CNPY3* findings in routine clinical diagnosis. *CNPY3* encodes an intracellular chaperone that supports immune function through Toll-like receptors (TLRs), but the effects of disease-associated *CNPY3* variants on immune responses are not well understood.

**Added value of this study**

This study reports clinical and genetic data from 50 affected individuals from 38 unrelated families with disease-causing *CNPY3* variants, substantially expanding the number of cases described to date. It provides a comprehensive description of the clinical spectrum, characterised by global developmental delay, intellectual disability, and early-onset, often intractable epilepsy, frequently accompanied by microcephaly, spasticity, stridor, growth impairment, and early mortality. By integrating detailed clinical data with functional analyses, this study helps to explain how genetic changes lead to disease and shows that different *CNPY3* variants affect protein function in distinct ways, thereby providing a variant-to-function framework.

**Implications of all the available evidence**

Our findings show that changes in the *CNPY3* gene affect immune signalling through diverse mechanisms, reflecting multiple levels of TLR function regulation by individual *CNPY3* variants. While all tested variants impair TLR signalling, they do so in different ways, modulating receptor trafficking and downstream pathways, such as the NLRP3 inflammasome. Despite this biological diversity, affected individuals share a set of clinical features, although symptom severity can vary widely. Together, these results underscore the complex biological and clinical consequences of *CNPY3* dysfunction and highlight the pivotal role of this gene in both brain development and immune function. These insights provide a clearer basis for interpreting genetic test results, linking specific gene changes to clinical features, and identifying potential therapeutic targets aimed at modifying disease-related immune pathways.

## Introduction

Developmental and epileptic encephalopathies (DEEs) denote a heterogeneous group of infantile neurological diseases with a mostly genetic aetiology that includes infantile epileptic spasms syndrome (IESS, formerly West syndrome), Dravet syndrome, Lennox-Gastaut syndrome, tuberous sclerosis complex and others.^1^^,^^2^ Biallelic variants in *CNPY3* (canopy FGF signalling regulator 3; HGNC:11968) were initially reported in 2018 as causal for IESS in three individuals from two unrelated families who presented with intractable seizures, severe developmental delay, brain atrophy, hippocampal malrotation, short stature and infantile death (DEE60; phenotype MIM # 617929).^3^ To date, recessive variants in *CNPY3* have been reported in a total of 14 patients from ten families exhibiting a similar phenotype (Table 1).^3^, ^4^, ^5^, ^6^, ^7^, ^8^, ^9^, ^10^ The pathogenic association between a loss of function in CNPY3 (protein canopy homologue 3, also known as PRAT4A; UniProt ID: Q9BT09) and DEE is supported by decreased CNPY3 levels in patient cell lines and animal models.^3^^,^^5^^,^^11^ *Cnpy3*^−/−^ mice resembled the clinical condition, exhibiting motor and behavioural abnormalities, EEG irregularities, impaired growth, and increased mortality.

**Table 1:** Previously reported individuals with biallelic *CNPY3* variants.

Affected individuals (families)	14 (10)
*CNPY3* variant types (zygosity, families)	missense (hom, 4), truncating (hom, 1), aberrant splicing (hom, 2), missense + truncating (comp-het, 3)
Developmental and epileptic encephalopathy, n/N	10/14
Seizures or epilepsy, n/N	12/14
Infantile seizure onset, n/N	10/10
Intractable seizures, n/N	5/7
Cerebral atrophy, n/N	8/8
Death in infancy, n/N	2/14

The table summarises genetic and clinical characteristics of previously reported individuals carrying biallelic *CNPY3* variants. Symptom frequencies are reported as number affected/number evaluated.

comp-het, compound-heterozygous; hom, homozygous.

References: Mutoh et al., 2018,^3^ Maddirevula et al., 2019,^7^ Hu et al., 2019,^6^ Xiang et al., 2021,^6^ Ghait et al., 2022,^5^ Agarwala et al., 2023,^4^ Rehman et al., 2025,^9^ and Xu et al., 2025.^10^

*CNPY3* (NM_006586.5) is located on chromosome 6p21.1 (genomic coordinates (GRCh38): 6:42,928,002–42,939,294), consists of six exons and encodes a 278 amino acid protein conserved across vertebrates. CNPY3 is widely expressed across human tissues, with the highest levels in the brain (notably cortex, hippocampus and cerebellum), thyroid and immune-related tissues (spleen and lymph nodes).^12^ Prominent neuronal expression of Cnpy3 was confirmed in mice, with age-dependent changes in expression and localisation to the endoplasmic reticulum and synaptosome.^13^ CNPY3 is confined to the ER by N- and C-terminal motifs,^5^^,^^14^^,^^15^ where, together with endoplasmin (also called HSP90B1, Grp94 or gp96) it facilitates the proper folding and trafficking of several Toll-like receptors (TLRs), including TLR1, TLR2, TLR4, TLR5, and TLR9, but not TLR3.^5^^,^^11^^,^^15^ Accordingly, experimental knock-out of CNPY3 results in incomplete N-linked glycosylation and defective cell surface expression of various TLRs.^5^^,^^11^ In addition, we recently reported a TLR-independent function of CNPY3 in inflammasome activation, as CNPY3-deficient macrophages showed impaired IL-1β and IL-18 release, irrespective of upstream TLR signalling.^5^ This is indicative of yet unknown substrates of CNPY3 affecting inflammatory signalling at multiple levels.

We hereby report on a cohort of 50 individuals from 38 unrelated families affected by CNPY3-DEE. Of these, 43 individuals from 33 families carry 19 homozygous or compound heterozygous *CNPY3* variants that have not been reported in public databases. Through this expanded cohort and in-depth analysis of clinical data, we provide a comprehensive characterisation and present pathophysiological, structural and biochemical analyses of selected variants, focussing on their functional impact on inflammatory signalling.

## Methods

### Patient enrolment and study conduct

This study is a cross-sectional analysis of all available individuals with biallelic *CNPY3* variants and a consistent neurodevelopmental phenotype. The cohort was assembled using GeneMatcher, ClinVar, DECIPHER and LOVD databases,^16^, ^17^, ^18^, ^19^ and through data sharing among collaborators worldwide. Additional cases were identified from the UCL Queen Square Genomics database (UK). Five families (F9, F12, F17, F28, and F31) had been reported previously and were included here to provide expanded phenotypic detail or add additional affected relatives.^4^, ^5^, ^6^, ^7^ Patients were recruited between October 2019 and June 2025, and the study analyses were conducted between November 2020 and December 2025.

### Clinical parameters

Clinical data were retrospectively extracted from medical records at participating centres using a structured template with predefined variables (Supplementary Data 1). Information on sex, consanguinity, country of origin, and ethnicity were self-reported by the patients or their guardians. Additional free-text clinical descriptions were compiled in the Supplementary Material–Case Reports. All datasets underwent centralised quality control; ambiguous entries were clarified with centres, and targeted queries were issued to retrieve missing key variables where possible. Clinical features were annotated using terms from the Human Phenotype Ontology (HPO)^20^ to ensure consistent terminology across centres, without reinterpretation of the original documentation. Anthropometric measures were converted to z scores using WHO Child Growth Standards and INTERGROWTH-21st references.^21^, ^22^, ^23^ Software R including R-package gigs was used for calculations and graphical displays.^24^ Potential sources of bias include incomplete reporting, variability in clinical work-up, and missing data. These were mitigated through follow-up queries, standardised HPO-based annotation, and exclusion of insufficiently documented variables. Analyses were restricted to available data without imputation.

### Phenotype severity score

A phenotype severity score (PSS) was developed to quantify clinical severity across seven domains—development/cognition, motor function, speech, epilepsy, EEG, growth, and respiratory involvement; details are provided in the Supplementary Material (Supplementary Methods). The PSS was designed as a pragmatic, disease-specific tool to facilitate standardised comparison of clinical severity across affected individuals. The seven domains were selected a priori based on recurrent and clinically meaningful manifestations observed across the cohort. Although intended for descriptive and comparative analyses, the PSS is not a formally validated clinical outcome measure. Given the rarity of CNPY3-associated disease and the absence of an independent external cohort, formal validation is currently not feasible. Each domain was scored from 0 to 3 according to predefined criteria, with higher scores indicating greater severity. The PSS was assigned independently by two experienced clinical raters, and interrater agreement was assessed using kappa statistics for individual domains and the intraclass correlation coefficient for total scores. Discrepancies were resolved by consensus, and consensus scores were used for all subsequent analyses. For each patient, a raw PSS was calculated as the sum of available domain scores. Missing domains were not imputed. To account for incomplete data, a normalised PSS was calculated by dividing the observed score by the maximum possible score of available domains and rescaling to a range of 0–21. A family-level sensitivity analysis was performed using family median PSS values.

### Phenotypic clustering

Unsupervised clustering of phenotypic profiles was performed using HPO annotations. To reduce sparsity and ensure stable similarity estimates, only HPO terms present in at least three individuals were retained for analysis. HPO ontology structure and metadata were retrieved using the ontologyIndex package in R. Pairwise phenotypic similarity between patients was calculated using the Lin semantic similarity metric implemented in the ontologySimilarity package. The resulting similarity matrix was converted into a dissimilarity matrix (1—similarity) and used for hierarchical agglomerative clustering with Ward.D linkage. Additional details of the clustering workflow are provided in the legend of Supplementary Figure S5.

### Radiological parameters

Available MRI imaging data were originally acquired during routine clinical examinations and were re-evaluated by a paediatric radiologist.

### Genetic sequencing and analysis

Genetic testing was performed using standard protocols at each participating centre. *CNPY3* variants were primarily identified by exome sequencing (n = 40), trio-based exome or genome sequencing (n = 3 each). In individual cases clinical exome, trio-based genome sequencing, or epilepsy gene panels were applied. Variant confirmation and segregation analysis were performed via Sanger sequencing. Variants located within the low-complexity repetitive region of *CNPY3* were retained for analysis but flagged and interpreted with increased caution due to the higher risk of alignment and calling artefacts.

*CNPY3* variants were annotated following HGVS guidelines (GRCh38/hg38, transcript NM_006586.5), with nomenclature verified using VariantValidator.^25^ Functional predictions for missense variants were obtained via SIFT, PolyPhen, CADD, MaxEntScan, SpliceAI, and AlphaMissense using Ensembl VEP.^26^ Population allele frequencies were obtained from gnomAD.^27^ PhyloP conservation scores for missense variants were obtained from Alamut Visual Plus v1.4 (Sophia Genetics).

Shared ancestry was assessed by genotype comparison of single nucleotide variants (SNVs) with a read depth ≥10 flanking each variant locus in all individuals carrying the respective variants to determine the minimal region of shared homozygosity.

### Antibodies and accessory reagents

Antibodies for flow cytometry: Anti-TLR2 (Miltenyi Biotec, Cat#130-120-138, RRID:AB_2752006) and corresponding isotype control (Miltenyi Biotec, Cat#130-113-446, RRID:AB_2733446). Antibodies for Western blot: CNPY3 (Proteintech, Cat#15215-1-AP, RRID:AB_11182172), TLR2 (Proteintech, Cat#66645, RRID:AB_3084262), FLT3 clone C-20 (Santa Cruz Biotech), HA-tag (Biolegend Cat#901513, RRID:AB_2565335). Secondary Antibodies IRDye 680RD Goat-anti-Rabbit (LICORbio Cat# 925-68071, RRID:AB_2721181) and IRDye 800CW Goat-anti-Mouse (LICORbio Cat#926-32210, RRID:AB_621842) for immunofluorescent detection, antibody diluent and blocking solutions (LI-COR Biotech, Lincoln, NE, USA). Antibodies for immunocytochemistry: Nogo/Reticulon4 (abcam, Cat# ab47085, RRID: AB_881718) and PRAT4A (Santa Cruz #sc-515151).

### Plasmids and cloning

The cDNAs of interest were amplified from available expression plasmids: CNPY3 from myc-CNPY3-BIOID2 plasmid (kind gift of Sachin Deshmukh, Jena), mCherry from pmCherry-N1 (Clontech, Cat.# 632523) and TurboID from V5-TurboID-NES pCDNA3 (kind gift from Alice Ting, Addgene plasmid Cat# 107169; http://n2t.net/addgene:107169; RRID:Addgene_107169). CNPY3 site-directed point mutagenesis was performed using KOD polymerase (Merck, Cat#71086-3). Mutants were subcloned into pCMV-HA (Clontech, Cat.# 631604) or lentiviral vectors pCDH-MSCV-MCS-EF1α-Puro (System Biosciences, Cat#CD710B-1) and pCDH-MSCV-MCS-EF1α-copGFP-T2A-Puro (System Biosciences, Cat#CD713B-1) either in fusion to TurboID (S156Y) or mCherry (all other mutants) by Gibson assembly (New England Biolabs, Cat#E2621). All plasmids were verified by Sanger sequencing. Primer and plasmid sequences are available upon request. All engineered constructs are denoted with superscripts indicating protein changes using the one-letter amino acid code, e.g., as CNPY3^S156Y^.

### Cell culture

HEK293T cells (ATCC, RRID:CVCL_0063) were cultured in DMEM/10% FCS (Gibco, Cat#10270106) at 37 °C, 5% CO_2_ and passaged every 2–3 days. THP-1 cells (ATCC, RRID:CVCL_0006) and CNPY3 variant THP-1 cell lines were grown in RPMI/10% FCS. Cell lines expressing CNPY3^S156Y^-TurboID were deprived of biotin according to a biotin-removal protocol^28^ prior to experimentation. Cells were routinely checked for mycoplasma contamination.

### HEK293T cell transfection

HEK293T cells were transfected by applying a 1:3 (w/w) plasmid DNA/Polyethylenimine (PEI, branched; SIGMA/Aldrich Cat#408727) mix in Opti-MEM (Thermo Fisher, Cat#11058021). Plasmid amounts were 2 μg/1.5 cm dish and 15 μg/10 cm dish. Efficiency was assessed 24 h post-transfection by fluorescence microscopy.

### Lentiviral transduction

Lentiviral particles were obtained by PEI-based transfection of HEK293T cells with pCDH plasmids in combination with packaging plasmids encoding VSV-G, pRSV and pMDL in a (1:2.5:5) ratio.^29^ THP-1 cells deficient in CNPY3 (CNPY3^KO^) were seeded in six well plates at a density of 4 × 10^5^ cells/well/2 ml standard RPMI/10% FCS medium.^5^ HEK293T culture supernatant, containing the pseudovirus, was harvested at 24, 48 and 72 h post transfection, filtered through a 0.2 μm syringe filter, concentrated by centrifugation (Amicon 50K, Cat#UFC901024) and added to the CNPY3^KO^ cells in the presence of 9 μg/ml polybrene (Merck, Cat#TR-1003-G). Plates were spun at 500 g for 1 h. Cells were subjected to three transduction rounds, followed by selection with 0.6 μg/ml puromycin). Stable cell lines co-expressing the variants R121Q and Q210^∗^ were enriched by flow cytometric sorting (BD Aria).

### Flow cytometry

1 × 10^6^ THP-1 cells were harvested and washed with ice cold PBS. Antibodies diluted in FACS buffer (PBS/2% FCS) were added and samples were incubated 10 min at 8 °C in the dark. Cells were pelleted by centrifugation, washed twice and analysed on a BD LSR Fortessa.

### Live-cell fluorescence microscopy

THP-1 cells reconstituted with mCherry tagged CNPY3 variants were treated with the live cell ER staining kit Blue Fluorescence (AAT Bioquest, Cat#22634) per manufacturer's instructions. 1 × 10^5^ cells were seeded in self-made 35 mm glass-bottomed dishes pre-coated with poly-lysine.^30^ Cells were washed with Opti-MEM and imaged live on an LSM900 laser scanning microscope equipped with a thermostated chamber in Airyscan mode.

### Immunocytochemistry

THP-1 cells and CNPY3 variant cell lines (1 × 10^6^) were seeded on poly-l-lysin (Sigma–Aldrich, Cat# P4707-50 ml) coated glass coverslips in 12-well plates, fixated by addition of 4% paraformaldehyde (PFA) (Roth, Cat# P087.6) and permeabilized with TBS containing 0.5% Triton X-100. Free PFA was quenched with 50 mM glycine/50 mM ammonium chloride). Antigen retrieval was performed for 15 min at 80 °C in 0.1 M Tris–HCl (pH 9.5) containing 6 M urea.^31^ After blocking in 10% non-fat milk/PBS for 1 h at RT, cells were incubated over night with primary antibodies in a Tris-buffered saline with 0.1% Tween-20/2% BSA at 4 °C, followed by secondary antibody incubation in PBS/2% BSA for 1 h. Coverslips were mounted and images were acquired on an LSM900 (Zeiss, Oberkochen, Germany) in Airyscan mode.

### SDS-PAGE and immunoblotting

1 × 10^7^ THP-1 cells/sample were lysed in buffer (Cell Signalling, Cat#9803) with protease inhibitors (Thermo Fisher, Cat#36978), sonicated and cleared by centrifugation. For chemoluminescence detection, supernatants were resolved by electrophoresis, transferred to PVDF membranes (Merck/Millipore, Cat#10600021) and imaged with a Fusion-FX (Vilber). For immunofluorescence detection, low-autofluorescence membranes (Merck/Millipore, Cat#IPFL00010) and fluorescent antibodies were used. Images were acquired on a LI-COR Odyssey XF.

### Glycosidase assay

Cell lysates were prepared as described above and glycoproteins were deglycosylated with PNGase F (New England Biolabs, Cat#P0704S) or Endo H (New England Biolabs, Cat#P0702S) under denaturing conditions following the manufacturer's instructions.

### Cell surface biotinylation assay

THP-1 cells were primed with 25 ng/ml human INF-γ (Proteintech, Cat#HZ-1301) overnight, then treated with 0.5 mg/ml EZ-Link NHS-SS-Biotin (Thermo Fisher, Cat#A39258) for 20 min at 4 °C. Residual crosslinker was neutralised with cold blocking solution (100 mM glycine + 0.2% BSA in PBS). Cells were lysed and biotinylated proteins captured on immobilised streptavidin (Thermo Fisher, Cat#29201). Beads were washed, and eluates analysed by western blotting.

### LC–MS analysis

Cell lysates and CNPY3 immunoprecipitates from THP-1/CNPY3^Q210^^∗^ cells were subjected to tryptic digestion and analysed by LC–MS using a Bruker timsTOF HT mass spectrometer (Bruker Daltonics, Bremen, Germany) operated in DIA-PASEF mode. Data were processed with DiaNN (version 1.8). The resulting data were used to confirm expression of the CNPY3^Q210^^∗^ protein.

### Enzyme-linked immunosorbent assay

THP-1 monocytes were differentiated into macrophages overnight with 100 ng/ml phorbol myristate acetate (Invivogen, Cat#tlrl-pma) in 96-well plates. TLR2 was stimulated with 100 ng/ml Pam3CSK4 (Invivogen, Cat#tlrl-pms) in Opti-MEM (Gibco, Cat#11058-021); TNF levels were detected in supernatants after 4 h. Cells were then treated with 6.5 μM nigericin (Invivogen, Cat#tlrl-nig) for stimulation of the NLRP3 inflammasome and IL-1β and TNF levels were assessed 3 h later with ELISA kits from Biolegend (Cat#437004) and R&D Systems (Cat#DY210-05), according to manufacturer's instructions.

### Data processing and image analysis

Flow cytometry data were analysed using FlowJo v10 software (BD Biosciences, Franklin Lakes, NJ, USA). Live cell pictures were processed by Zen 3.5 Blue Edition software (Zeiss, Oberkochen, Germany), and Western blot images by ImageJ software (National Institutes of Health, Bethesda, MD, USA).

### AlphaFold structure prediction

The initial sequences of CNPY3 (Q9BT09) and Grp94 (P14625/NP_003290.1) were obtained in FASTA format from the UniProtKB database. Structure predictions of CNPY3, CNPY3 without signal sequence (residues 1–30), CNPY3 variants, as well as their complexes with Grp94 were performed using AlphaFold2 (v2.3.2; alphafold2_multimer_v3 model weights; AlphaFold git commit hash f715f016d875e2fd8515457af2b12277a716b9b0, dated 1 November 2023) deployed locally (CentOS Linux 7.9.2009; Python v3.10.13; HMMER v3.4; HH-suite v3.3.0; Kalign v3.4.0; CUDA ptxas v11.7.64) (Evans et al., 2022; bioRxiv preprint).^32^ Calculations were run on servers equipped with AMD EPYC 7F52 16-core processors and NVIDIA A100-PCIE-40GB GPUs (CUDA 12.2; driver version 535.54.03). Multiple sequence alignments were generated using JackHMMER and HHblits against the full sequence databases (UniRef90, BFD, MGnify, Uniprot, and PDB; accessed between 17 January 2024 and 7 May 2024). For each target complex, 25 structural models were generated using five random seeds across all five model parameter sets. Models were ranked by the ipTM + pTM score, and the top-ranked model was selected for analysis. Model confidence was assessed using per-residue pLDDT scores and predicted aligned error (PAE) plots. Two types of calculations were performed: (A) single-chain predictions of the wild type and variant CNPY3 (MSA pairing disabled; -p false) and (B) complex predictions of CNPY3 with Grp94. In both cases, the multimer model preset was used, as alphafold2_multimer_v3 provides reliable predictions for both single-chain and multi-chain inputs.^32^

### Ethics

The study was approved by the Jena University Hospital institutional review board (ethics approval #2019-1602) and by all participating centres. Written informed consent was obtained from all participants or their legal guardians, and the study was conducted in accordance with the 2024 Declaration of Helsinki.

### Statistics

Clinical data were analysed using descriptive statistical methods, including median, minimum and maximum values and interquartile ranges (IQR). Missing data were addressed by restricting analyses to individuals with available data; no data were imputed. Variables with insufficient documentation were excluded from descriptive analyses, and the PSS was normalised to account for incomplete domains. No formal sensitivity analyses were performed for the descriptive analyses, as missingness was generally low for the principal disease-defining variables and unlikely to have materially affected the main descriptive findings. To evaluate the impact of incomplete PSS domain data, PSS-based comparisons were repeated after sequential exclusion of individuals with incomplete PSS domain data.

Interrater reliability of the PSS was assessed using Cohen's kappa for individual domains and the intraclass correlation coefficient for the overall score. To account for potential familial clustering, key PSS analyses were repeated using family-level median PSS values, such that each family contributed a single value.

Statistical tests were selected according to the specific analytical question and the distributional properties of the data. To determine whether parametric or non-parametric testing was appropriate, data sets with n ≥ 5 were assessed for normality using the Shapiro–Wilk test. If subgroups within a comparison deviated from normality, or if normality was not consistently met across subgroups, non-parametric tests were applied. Otherwise, clinical data were analysed using parametric tests.

Functional data were obtained from at least three independent experiments and are presented as mean ± SD unless stated otherwise. In accordance with standard practice for molecular biology experiments with small numbers of independent biological replicates, parametric analyses were applied.

The statistical test used for each analysis is indicated in the corresponding figure legend or in the main text. Parametric analyses included one-sample *t*-tests and one-way ANOVA, followed by Šídák's post hoc test to control for type I error. Non-parametric analyses included two-tailed Mann–Whitney *U* tests and Kruskal–Wallis tests, followed by Dunn's multiple-comparison test where appropriate. *P*-values ≤0.05 were considered statistically significant (∗∗∗∗ *P* ≤ 0.0001; ∗∗∗ *P* ≤ 0.001; ∗∗ *P* ≤ 0.01; ∗ *P* ≤ 0.05; ns *P* > 0.05).

### Role of funders

The funders had no role in the study design, data collection, data analysis, data interpretation, or writing of the report.

## Results

### Clinical characterisation of affected individuals with biallelic *CNPY3* variants

The cohort comprises 43 individuals with biallelic *CNPY3* variants from 33 families, together with 7 previously published cases from 5 families, yielding a total of 50 individuals from 38 unrelated families, of whom 48% were female (Fig. 1A). Individuals were aged 2 months to 41 years (median 4.0 years, IQR 1.7–7.0 years; Fig. 1B) at their last assessment. Families originate primarily from Southern Asia (56%) and Western Asia (20%), but also from Northern Africa (10%), Europe (6%) and Latin America (4%) (details see Supplementary Data 1 and Supplementary Figure S1).

**Fig. 1:**
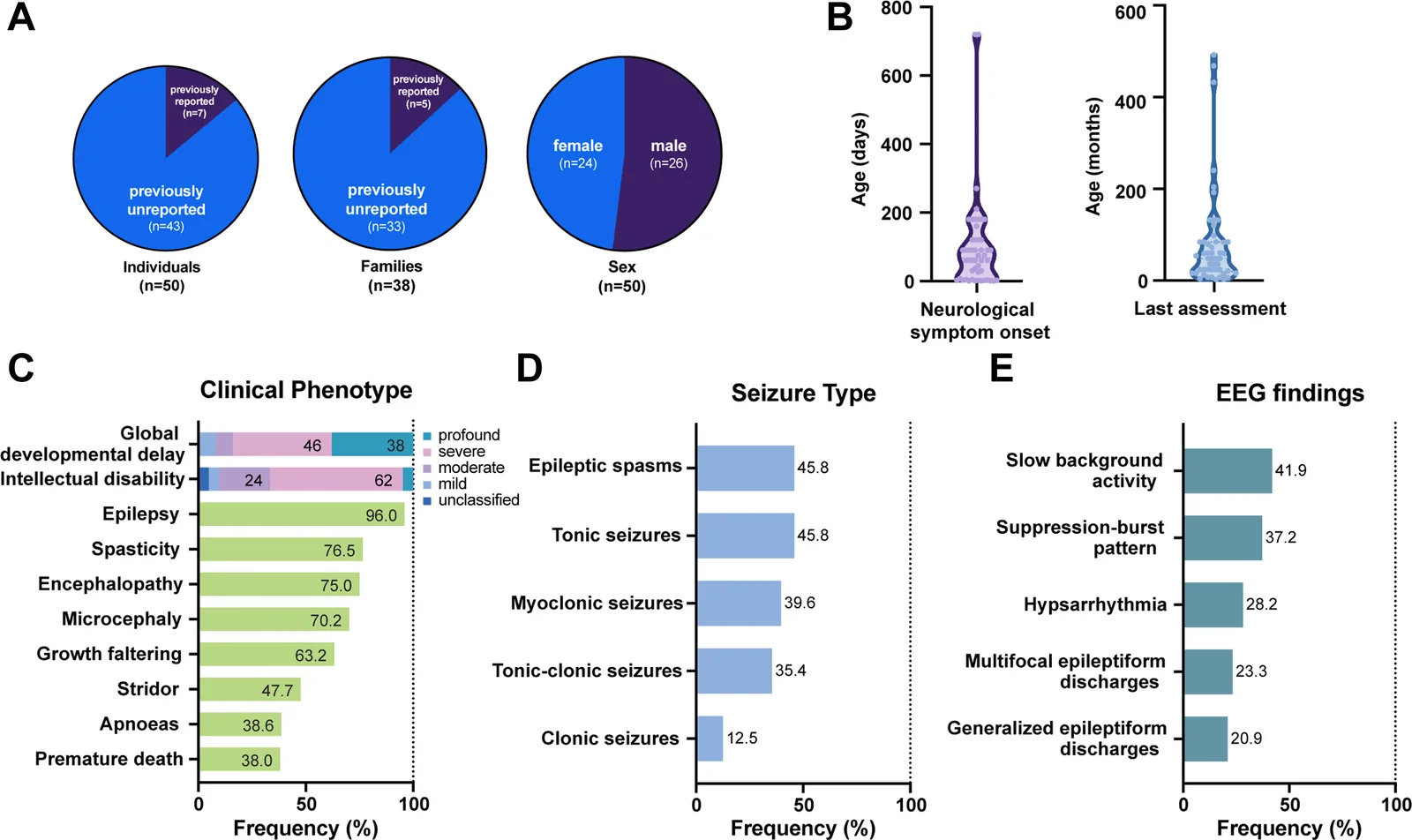
**Demographics and clinical characteristics of patients with CNPY3-DEE.** (**A**) Cohort demographics, showing the composition of the study cohort comprising 50 individuals from 38 unrelated families, including the relative proportions of previously reported and unreported individuals/families, with 48% female individuals. (**B**) Age distributions, shown as violin plots for age at neurological symptom onset (days) and age at last assessment (months) (n = 50). (**C**) Prevalence of major clinical features in the cohort, expressed as frequency (%), with denominators as follows: global developmental delay (n = 50), intellectual disability (assessed in patients aged ≥5 years; n = 21), epilepsy (n = 50), spasticity (n = 47), encephalopathy (n = 48), microcephaly (n = 47), growth faltering (n = 49), stridor (n = 44), apnoeas (n = 44), and death (n = 50). (**D**) Seizure types, reported relative to patients with epilepsy (n = 48). (**E**) EEG findings, reported relative to patients with available EEG data (n = 43).

Common pregnancy-related complications occurred in 33.3% (16/48) of pregnancies, within the range of the general population (details see Supplementary Figure S2). Birth histories were mostly unremarkable, while postnatal abnormalities (respiratory distress, transient tachypnoea, apnoea/cyanotic episodes, hypoxia, asphyxia and stridor) were noted in 42.2% (19/45).

Onset of neurological symptoms occurred between the first day of life and 5 years of age, (median 68 days, IQR 4–120 days) with seizures in 84% (42/50), developmental delay in 24% (12/50), muscular hypotonia in 18% (9/50), apnoeas in 8% (4/50) and irritability in 6% (3/50). Most cases (92%) presented within the first 6 months of life, including 30% during the neonatal period.

Global developmental delay was present in all individuals, classified as profound (38%, 19/50), severe (46%, 23/50) and moderate or mild (each 8%, 4/50). 52% (26/50) of patients did not achieve any motor milestones, 18% (9/50) walked independently, while 73.3% (33/45) remained non-verbal. Developmental regression was observed in 32.4% (12/37). Intellectual disability (assessed in patients aged ≥5 years) was reported as severe in 61.9% (13/21) and moderate in 23.8% (5/21). Microcephaly was reported in 70.2% (33/47). Encephalopathy (defined as diffuse brain dysfunction, altered mental state, cognitive impairment, and neurological symptoms) was observed in 75% of cases (36/48) and spasticity in 76.5% (36/47) without evidence of an additional consistent movement disorder (Fig. 1C). Visual (19/36) and hearing (6/26) impairment were observed, but not attributable to specific sensory organ dysfunction.

Epilepsy manifested in 96.0% (48/50) of individuals. Generalised seizures were observed in 93.8% (45/48) and focal seizures in 25.0% (12/48). The most common seizure types were epileptic spasms and tonic seizures (each 45.8%, 22/48), followed by myoclonic (39.6%, 19/48), tonic-clonic (35.4%, 17/48) and clonic (12.5%, 6/48) seizures (Fig. 1D). Percentages overlap because multiple seizure types could occur in the same individual. Status epilepticus was documented in 29.8% (14/47) of cases. Episodic apnoeas or cyanosis occurred in 38.6% (17/44), not consistently linked to seizure activity. EEG findings—allowing for multiple per individual—included slow background activity (41.9%, 18/43), suppression-burst pattern (37.2%, 16/43), hypsarrhythmia (28.2%, 11/43), multifocal epileptiform discharges (23.3%, 10/43) and generalised epileptiform discharges (20.9%, 9/43) (Fig. 1E). Over the treatment course, patients received a median of four anti-seizure medications (IQR 2–6; range 1–12) and 5 patients (10.4%) were treated with a ketogenic diet. Intractable epilepsy was observed in 86.0% of cases (37/43) and no single drug demonstrated superior efficacy across the cohort.

Growth faltering was reported in 63.2% (31/49 individuals), accompanied by feeding difficulties (67.3%, 33/49), recurrent vomiting (56.5%, 26/46), tube feeding (41.3%, 19/46) and chronic constipation (52.3%, 23/44). Body measurements assessed at the last examination in a subset of the cohort, using available WHO growth standards, showed median z scores of −3.03 for body weight (range −7.56 to 3.18; IQR −4.62 to −1.72; n = 39), −3.42 for body length (range −10.1 to 0.79; IQR −5.11 to −1.70; n = 24), and −3.73 for head circumference (range −7.65 to −1.16; IQR −5.01 to −2.71; n = 24). Extreme growth restriction (z score ≤ −4) was observed in 33.3% (13/39) of patients for body weight and in 37.5% (9/24) for both body length and head circumference. At birth, a significant negative deviation from the reference z-score of 0 was observed only for body weight; over time, however, by the time of the last examination, all growth parameters exhibited highly significant negative deviations, without substantial differences between them (Supplementary Figure S3). Body measurements at the last examination, plotted against WHO growth reference percentiles, are shown in Supplementary Figure S4.

Recurrent infections were reported in 40.8% (20/49) and decreased immune function in 10.2% (5/49). Stridor and laryngomalacia, specifically assessed because of their prominence in index patients, were reported in 47.7% (21/44) and 41.0% (16/39), respectively. No consistent syndromic pattern could be identified.

Of the 50 individuals, 19 (38%; from 16/38 families) died prematurely between ages 3 and 132 months (median 25 months; IQR 16–53 months). Circumstances of death were available for 15 individuals, with most deaths (11 cases) being respiratory-related (pneumonia, aspiration, or progressive apnoeas), followed by seizure-related (four), infection-related (three) and cardiac-related (two) causes. In addition, 17 affected siblings from 10 families, in whom genetic testing had not been performed, also died prematurely. Overall, premature death of neurologically affected individuals was observed in 57.9% (22/38) of families.

Data completeness was high for the principal neurological variables, including global developmental delay, intellectual disability, epilepsy, encephalopathy, and other core neurological features (<10% missing values). Moderate missingness (10–20%) affected selected variables, including developmental regression, intractable epilepsy, and respiratory manifestations, whereas higher rates (>20%) were observed mainly for anthropometric measurements and sensory assessments, reflecting the retrospective multicentre design and variable availability of historical clinical documentation (overview provided in Supplementary Data 2).

Detailed individual data, including information not shown here, are provided in Supplementary Data 1; clinical descriptions are available as Case Reports in the Supplementary Material.

To quantify overall clinical severity, we applied a phenotype severity score (PSS) encompassing seven domains: development/cognition, motor function, speech, epilepsy, EEG findings, growth, and respiratory involvement. Interrater agreement for the PSS was substantial to excellent across all domains (Cohen's kappa 0.74–1.0), with a high intraclass correlation coefficient for total scores (ICC 0.98, 95% CI 0.96–0.99). Severity was greatest in epilepsy and, among the graded domains, in motor function and development/cognition, whereas growth and respiratory involvement were least affected; speech was frequently maximally affected but was assessed dichotomously (score 0 or 3 only) (Fig. 2A). PSS values ranged from 2 to 20 and were skewed toward higher severity, indicating substantial variability with predominantly severe phenotypes (median 16, IQR 11.8–18.0) (Fig. 2B, Supplementary Data 3). Within families, PSS values were generally similar among affected individuals, indicating consistent intrafamilial severity, although moderate variability was observed in two families (F26, F38).

**Fig. 2:**
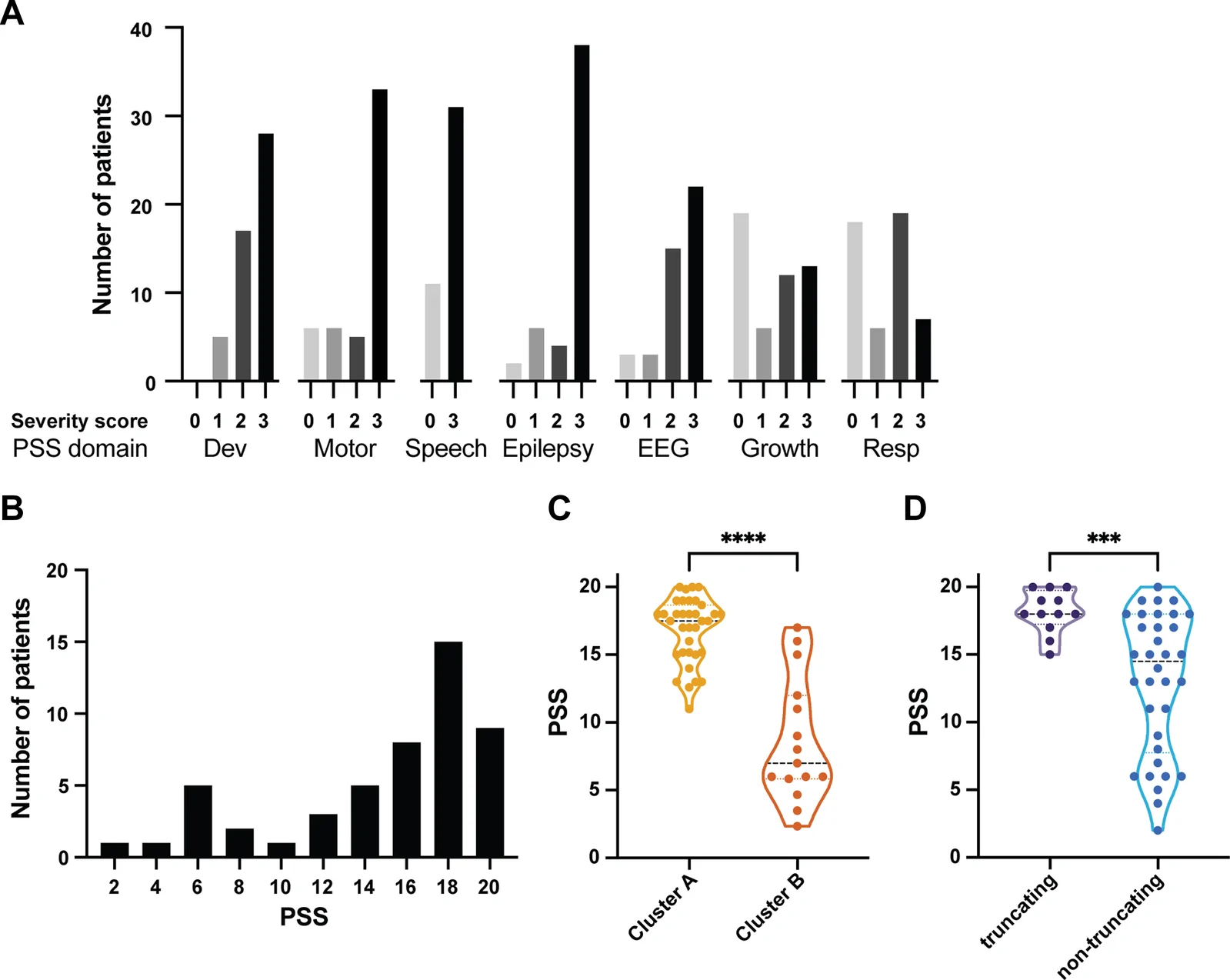
**Phenotype Severity Score (PSS) across clinical clusters and genotype groups.** A domain-based clinical severity score (range 0–21) was applied to all patients. (**A**) Distribution of raw severity scores across the seven domains (n = 50). (**B**) Frequency distribution of normalised PSS values in the CNPY3 cohort (n = 50). (**C**) Differences in PSS distribution between two patient clusters identified by unsupervised hierarchical clustering of HPO terms (n = 50). (**D**) Differences in PSS distribution between truncating and non-truncating genotype groups (n = 46). (**C**, **D**) Data are shown as violin plots, with medians and interquartile ranges. Dev: development, Resp: respiratory. Statistical test: two-tailed Mann–Whitney U test; ∗∗∗ *P* < 0.001; ∗∗∗∗ *P* < 0.0001.

The application of an unsupervised clustering approach based on a more detailed set of HPO terms suggested the presence of two phenotypic groups: cluster A (35/50 patients, 70%) and cluster B (15/50 patients, 30%) (Supplementary Figure S5, Supplementary Data 3). All individuals from the same family clustered together, with 76.3% (29/38) of families assigned to cluster A and 23.7% (9/38) to cluster B.

Patients in cluster A had significantly higher PSS values than those in cluster B, with some overlap between clusters: cluster A, median 17.5 (IQR 15.2–18.7; range 11–20) vs cluster B, median 7.0 (IQR 5.8–12.0; range 2–17; two-tailed Mann–Whitney U test, *P* < 0.0001) (Fig. 2C). These findings remained robust in a family-level sensitivity analysis and after sequential exclusion of individuals with incomplete PSS domain data (Supplementary Figure S6A and B).

### Neuroradiologic imaging findings

In 15 available MRI scans from 12 patients, four patients exhibited hippocampal abnormalities. Two cases (F31:II.9, Fig. 3A and B; F5:II.1, Fig. 3C–E) showed left-sided incomplete hippocampal inversion (IHI), with additional left-sided temporal and frontal atrophy in F31:II.9. However, IHI was not detected in other individuals sharing the same genotype as F31:II.9. One additional case (F11:III.3: Fig. 3F–I) exhibited generalised brain and hippocampal atrophy with hippocampal morphology indicative of IHI, and one case (F2:II.5: Fig. 3J) showed bilateral hippocampal atrophy. Additional findings included varying degrees of regional brain atrophy. Ventriculomegaly was observed in four patients, and two cases showed focal widening of the left temporal subarachnoid space. White matter abnormalities were noted in five cases, including two patients with periventricular cysts consistent with periventricular leukomalacia. Variants of the septum pellucidum were observed in five cases. Additional MRI and CT findings from 27 cases are described in Supplementary Data 1, including diffuse brain atrophy, reduced white matter volume, periventricular white matter changes with cyst formation, delayed myelination, abnormal hippocampal internal architecture, and cerebellar atrophy or hypoplasia. In 13 cases, neuroimaging was reported as normal.

**Fig. 3:**
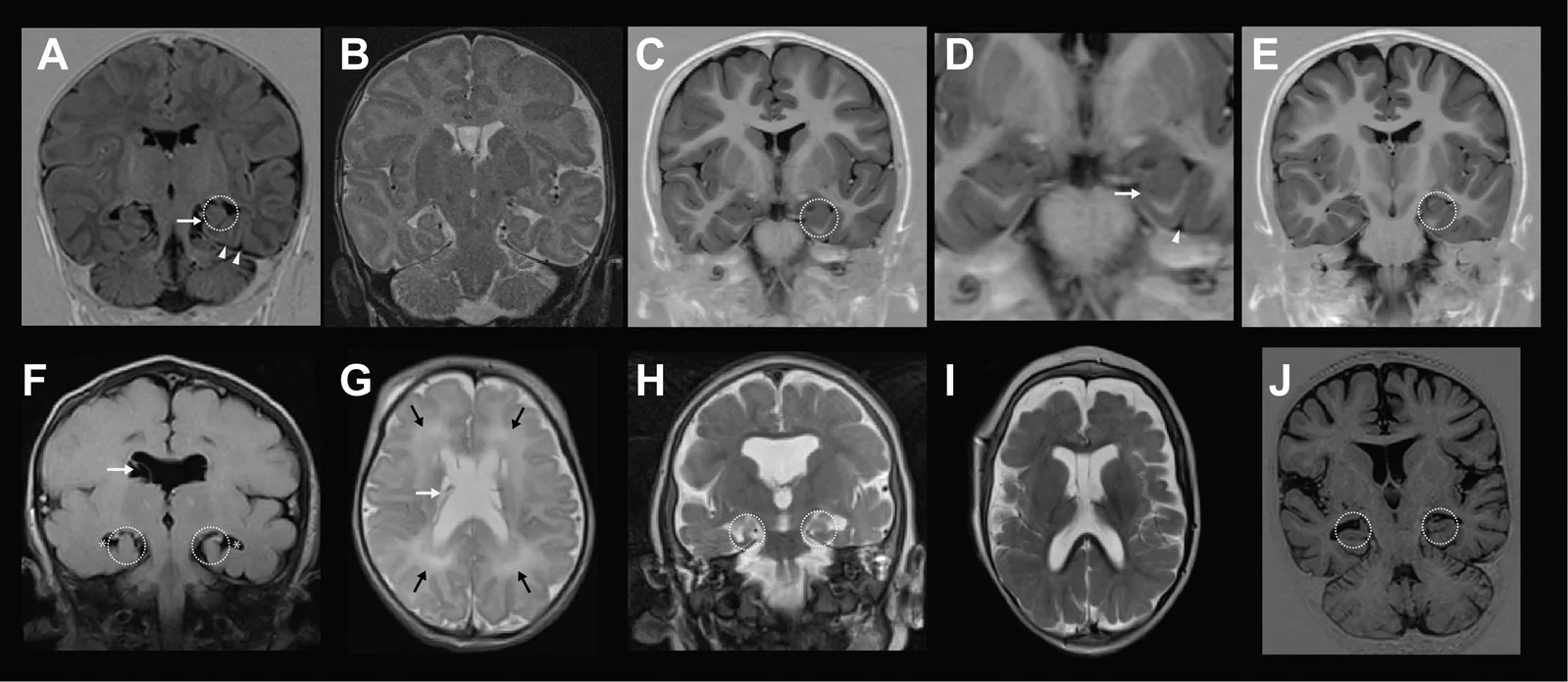
**Neuroradiologic findings.** F31:II.9 at 15 weeks of age: Coronal T1IR (**A**) and T2-weighted (**B**) MR imaging demonstrate incomplete hippocampal inversion (IHI) on the left (circle), characterised by medial orientation of the hippocampal body, verticalization, and increased depth of the collateral and occipitotemporal sulci (arrowheads) extending beyond the subiculum (arrow). Additional findings include a cavum septi pellucidi and mildly prominent subarachnoid spaces on the left, likely secondary to suspected atrophy in the left frontal and temporal lobes. F5:II.1 at 4 years of age: Coronal T1IR-weighted images (**C**; **D**, magnified view of **C**; **E**) show left-sided IHI (circle), characterised by a vertically oriented, rounded hippocampal body and a deep, vertically oriented collateral sulcus (arrowhead) extending beyond the subiculum (arrow). F11:III.3 at 5 days of age: Coronal FLAIR (**F**) and axial T2-weighted (**G**) MR imaging show abnormal white matter signal (black arrows), periventricular cysts (white arrow) and ventricular enlargement. Additional findings include partial septal agenesis, partial cavum septi pellucidi, and abnormal configuration of both hippocampi (circles) with dilatation of the temporal horns (asterisks), raising suspicion of IHI. Follow-up at 21 months of age: Coronal (**H**) and axial (**I**) T2-weighted images show a persistent abnormal hippocampal configuration (circles), compatible with atrophy or IHI. Additionally, there is ventriculomegaly with enlarged subarachnoid CSF spaces, consistent with reduced brain volume. F2:II.5 at 9 years of age: Coronal T1IR-weighted MR imaging (**J**) shows abnormal hippocampal configuration (circles) consistent with bilateral hippocampal atrophy.

### Genetic analysis

Genetic analysis of 50 individuals from 38 families identified 25 distinct *CNPY3* variants, 76% (19/25) of which were absent from public variant databases, and 56% (14/25) restricted to a single family. Consanguinity was present in 92% (46/50) of cases. 95% of variants (36/38 families, 48 individuals) occurred in homozygosity and 5% (2/38 families, 2 individuals) in compound heterozygosity. Genetic findings are summarised in Table 2, with pedigrees shown in Supplementary Figure S7. According to ACMG/AMP criteria, 22 variants (71.0%) were classified as of uncertain significance, seven (22.6%) as likely pathogenic, and two (6.5%) as pathogenic. Supplementary Data 4 shows a variant-specific overview of genetic details, computational prediction, population allele frequencies, ACMG classification and classification criteria applied for each variant.

**Table 2:** Recessive *CNPY3* variants identified in the CNPY3-DEE cohort and in previously reported cases.

*CNPY3* variant (NM_006586.5, NP_006577.2)	Variant consequence	*In silico* prediction (CADD PHRED score)	Conservation score (PhyloP)	Allele frequency (gnomAD)	ACMG class^b^	Affected families (cohort ID)	Zygosity^c^	Reference(s)
c.59_76del, p.(Leu20_Leu25del)	inframe deletion	.	.	.^a^	3	F1	hom	
c.62_76dup, p.(Leu21_Leu25dup)	inframe insertion	.	.	.^a^	3	F2, F3	hom	
c.65_76del, p.(Leu22_Leu25del)	inframe deletion	.	.	.^a^	3	F4	hom	
c.65_76dup, p.(Leu22_Leu25dup)	inframe insertion	.	.	.^a^	3	F5	comp-het	
c.172G>A, p.(Glu58Lys)	missense	29.7	8.95	.	3	F6	hom	
c.275C>T, p.(Ser92Leu)	missense, splice region	33	7.04	0.00001239	3	F7, F8, *F9*, F10	hom	4,8
c.275 + 1G>A, p.(?)	splice donor	35	8.95	0.000002401	5	F11, F5	hom, comp-het	
c.283C>G, p.(Arg95Gly)	missense	31	3.34	.	3	.	comp-het	9
c.283C>T, p.(Arg95Trp)	missense	30	3.34	0.000002488	3	.	comp-het	10
c.284G>A, p.(Arg95Gln)	missense	32	8.95	0.00001244	3	*F12*	hom	*6*
c.292G>A, p.(Glu98Lys)	missense	33	8.95	0.00001553	3	F13, F14	hom	
c.329A>G, p.(Tyr110Cys)	missense	29.8	8.34	0.000001594	3	F15, F16, *F17*	hom	*4*
c.337C>T, p.(His113Tyr)	missense	26.5	7.04	.	3	F18	hom	
c.362G>A, p.(Arg121Gln)	missense	32	8.95	0.000002059	3	F19, F20	hom, comp-het	
c.370A>C, p.(Lys124Gln)	missense, splice region	33	8.34	.	3	F21	hom	
c.373G>C, p.(Gly125Arg)	missense, splice region	33	9.00	.	4	.	comp-het	3
c.391G>A, p.(Glu131Lys)	missense	24.9	4.42	.	3	F22	hom	
c.436G>T, p.(Asp146Tyr)	missense	32	9.19	0.000684	3	F23, F24	hom	
c.446A>G, p.(Tyr149Cys)	missense	28.4	6.71	0.00001983	3	F25	hom	
c.467C>A, p.(Ser156Tyr)	missense	27.1	5.35	.	3	F26	hom	
c.495 + 1G>A, p.(?)	splice donor	35	9.04	0.000001368	4	.	hom	3
c.496-3_496-2del, p.Cys166ValfsTer83	splice acceptor	.	.	.	5	F27, *F28*, F29, F30	hom	*7*
c.548del, p.(Asn183ThrfsTer7)	frameshift	.	.	.	4	*F31*, F32	hom	*5*
c.628C>T, p.(Gln210Ter)	stop gained	37	0.14	.	3	F20	comp-het	
c.702_720dup, p.(Ser241GlyfsTer7)	frameshift	.	.	.	4	.	comp-het	3
c.707_716del, p.(Ala236GlyfsTer49)	frameshift	.	.	.	4	F33	hom	
c.733_736del, p.(Gln245GlyfsTer42)	frameshift	.	.	.	4	F34	hom	
c.734_737del, p.(Gln245ArgfsTer42)	frameshift	33	.	0.000007436	4	F35	hom, comp-het	10
c.825del, p.(Asp276MetfsTer12)	frameshift	22.6	.	0.000006581	3	F36	hom	
c.832C>A, p.(Leu278Ile)	missense	25.2	5.23	.	3	F37, F38	hom	
c.834del, p.(Ter279GluextTer8)	frameshift	.	.	.	3	.	comp-het	9

Shown are predicted variant consequences, evolutionary conservation, allele frequency, affected families, zygosity and classification according to ACMG/AMP. Previously reported families (with references) are italicised if part of the cohort.

CADD PHRED scores >10, >20, and >30 indicate variants predicted to be among the 10%, 1%, and 0.1% most deleterious substitutions in the human genome, respectively. PhyloP scores are based on 100-way vertebrate alignments and range from −19 to +11; positive scores indicate evolutionary conservation. Allele frequency refers to the combined population frequency in gnomAD v4.1.0.

a Variants within low complexity region, heterozygous frequencies not shown due to possibly dubious variant annotation and quality.

b ACMG/AMP classification according to ACMG guidelines: 3 = uncertain significance, 4 = likely pathogenic, 5 = pathogenic. Detailed ACMG evidence codes are provided in Supplementary Data S4.

c In cases with multiple families, zygosity refers to different individuals carrying the same variant.

Non-truncating variants (68%, 17/25) were most frequent, including missense variants (52%, 13/25), *in frame* deletions (8%, 2/25) and *in frame* duplications (8%, 2/25). Truncating variants (28%, 7/25) comprised frameshift variants (20%, 5/25), 1 nonsense variant, and 1 intronic deletion (4% each). One intronic splicing variant of uncertain consequence was detected (4%, 1/25) and 2 additional variants were predicted to disrupt splicing according to MaxEntScan and SpliceAI. The intronic splicing variant c.275 + 1G>A (F5, F11) is predicted to result in a skipping of exon 2, causing a frameshift and protein truncation. RT-PCR confirmed aberrant splicing for c.496-3_496-2del (F27–F30, Supplementary Figure S8).^33^ Exonic variants adjacent to conserved splice sites were not predicted to affect splicing.

Variants were distributed across all exons, with clustering in exons 3 and 6, and 12 of 13 missense variants mapping to the DUF3456 domain (Fig. 4). 5 of 7 protein-truncating variants were located in the last exon, likely allowing the corresponding mRNAs to escape nonsense-mediated decay.

**Fig. 4:**
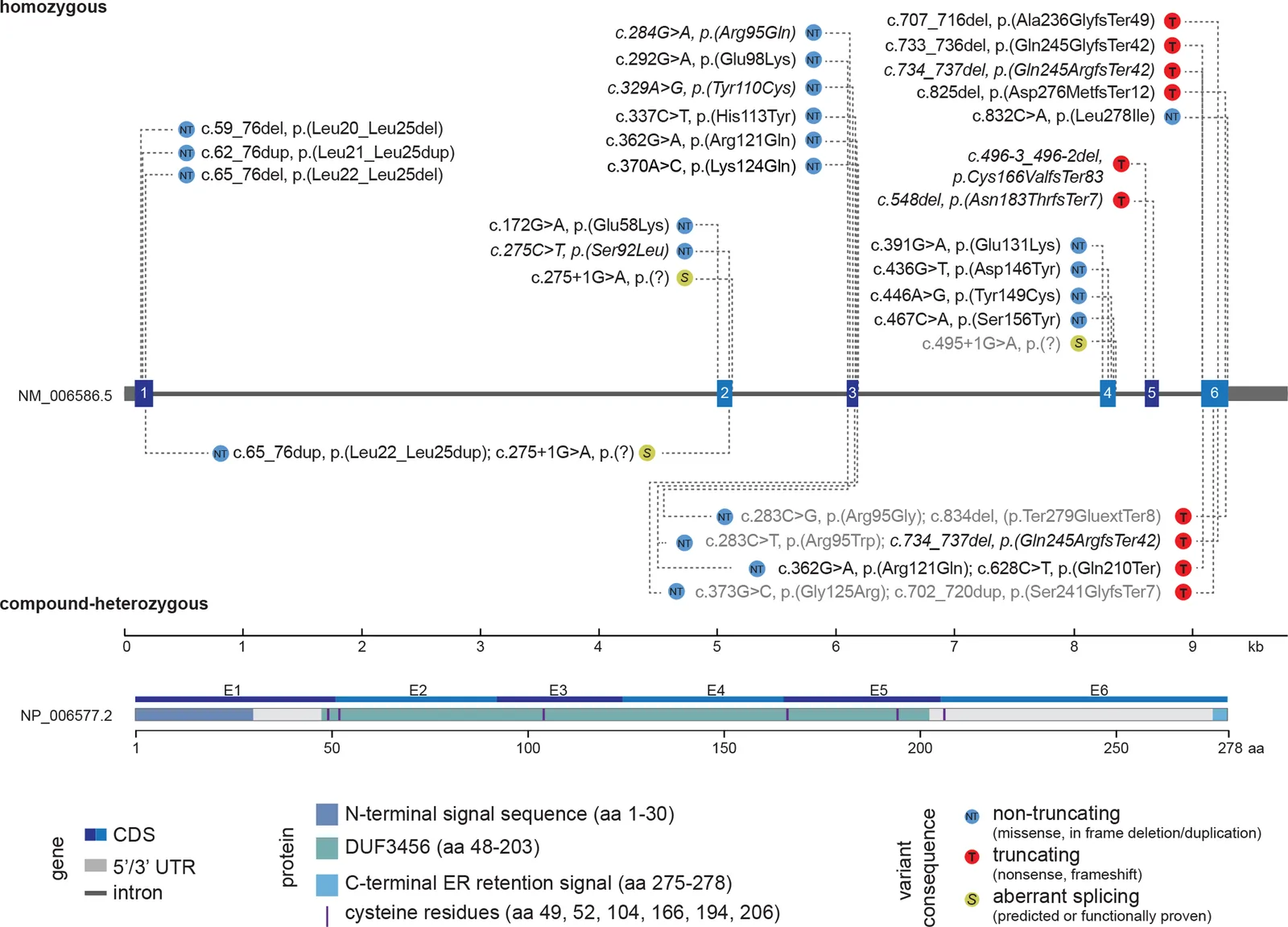
**Genomic andprotein structure of CNPY3 with observed variants.** Gene structure of *CNPY3* and protein structure of the encoded protein. Variants are depicted according to their genomic position and predicted molecular consequence. Homozygous variants are shown in the upper panel, and compound heterozygous variants in the lower panel. Previously reported variants are shown in grey; those also identified in our cohort are indicated in italics. Protein features are shown to scale. CDS, coding sequence; DUF3456, domain of unknown function.

The *CNPY3* locus includes a short low-complexity repetitive region (chr6:42,929,619–42,929,646) characterised by overlapping small insertions and deletions within a repetitive TGC motif. As this segment is flagged as a low-complexity region (LCR) in genome databases, variant calling may be unreliable due to alignment ambiguity and read-mapping errors; in our study, variants in this region were therefore not excluded a priori but interpreted with particular caution and independently validated by Sanger sequencing (Supplementary Figure S9). We identified several variants in this region, comprising either homozygous in-frame deletions or duplications, or a compound-heterozygous combination with a splice donor variant identified in trans; the same splice variant was also observed in the homozygous state in an independent case, supporting the functional relevance of the in-frame variants despite their location within an LCR. Pathogenicity assessment placed limited weight on allele frequency and instead relied on phenotype consistency, segregation analysis, and exclusion of alternative genetic causes. No homozygous occurrences of these variants are reported in gnomAD, despite an increased background of variation at this locus. Nevertheless, interpretation of variants in this LCR requires careful distinction between pathogenic and non-pathogenic variation.

None of the variants was observed in a homozygous state in gnomAD, or in the Queen Square Genomics and Tuebingen in-house databases, except for the cases included in this cohort. *In silico* analysis by AlphaMissense revealed that most missense variants are predicted to be likely pathogenic (84%, 11/13) and affect a highly conserved nucleotide according to PhyloP (61%, 8/13). Almost all missense variants were predicted deleterious (SIFT ≤0.01 in 92%, 12/13; CADD >24 in 100%, 13/13) and probably damaging (PolyPhen >0.9 in 84%, 11/13) (Supplementary Data 4).

Recurrent variants were identified in unrelated families from the same geographic regions: c.62_76dup, c.436G>T, and c.548del were each observed in two families; c.329A>G in three families; and c.275C>T and c.496-3_496-2del in four families. Analysis of c.496-3_496-2del (Saudi Arabia) and c.548del (Syria), revealed identical homozygous haplotypes spanning 2.1 and 1.03 Mb across the respective gene regions (chr6: 42048581–44113981 and chr6: 42194650–43224531), consistent with a common ancestral origin (Supplementary Data 5).

Within the cohort, two patients carried potentially competing genetic alterations: F8:II.3 with a homozygous, likely pathogenic variant in *ADAR*, and F19:II.1 with compound heterozygous variants (one likely pathogenic and one benign) in *HERC2*. However, clinical phenotypes, variant configuration and segregation analysis (Supplementary Data 1), together with recurrence of the respective *CNPY3* variants in other families, point to *CNPY3* as the most likely cause.

PSS values were significantly higher in patients with homozygous truncating variants (median 18.0, IQR 17.3–19.8; range 15–20) than in those with homozygous non-truncating variants (median 14.5, IQR 7.8–18.0; range 2–20; two-tailed Mann–Whitney U test, *P* = 0.0004). This difference remained significant after sequential exclusion of individuals with incomplete PSS domain data (Supplementary Figure S6C). Variants in compound heterozygosity and those with uncertain consequences (i.e. splice site variants without functional investigation) were excluded from statistical analysis (final n = 46). Variability differed significantly between genotype groups: patients with truncating variants showed a narrow distribution of high PSS values, whereas those with non-truncating variants exhibited substantially greater variability, spanning the full range observed in the truncating group (Fig. 2D). Missense variants did not show a consistent relationship with PSS, with variants distributed across a broad range of severity scores. Results were unchanged in family-level and variant-classification sensitivity analyses (Supplementary Figure S6D and E). In-frame variants located in the LCR showed PSS values in the moderate to high range, with insertions tending toward higher scores than deletions (13–19 vs 11–15). Individuals with compound-heterozygous variants showed intermediate to high PSS values (range 12–18). The splice site variant c.275 + 1G>A, which was not functionally investigated for its consequence, showed a high PSS value when observed in homozygous state (range 15–18). Intrafamilial PSS variability was low for most variants observed in homozygous state in multiple individuals within one family (median within-family range of 4.0 points, maximum 9). Within identical genotypes, PSS values showed low to moderate variability overall (median within-genotype range of 4.4 points, maximum 11), which was most pronounced in non-truncating genotypes, where several variants displayed marked discordance (Supplementary Data 3).

Comparison of variant consequences between the two phenotypically assigned clusters showed that cluster A included 45.7% (16/35) of patients with homozygous missense variants, followed by frameshift (20.0%, 7/35), splice donor or acceptor (17.1%, 6/35), and in-frame insertion or deletion variants (14.3%, 5/35); one patient carried compound heterozygous missense and nonsense variants. In cluster B, 86.7% (13/15) of patients carried homozygous missense variants, one patient a homozygous splice acceptor/frameshift variant, and one patient compound heterozygous in-frame insertion and splice donor variants.

### Protein structure prediction reveals conformational deviations in disease-associated variants

To explore structural consequences of CNPY3 variants, we performed artificial intelligence-based structure predictions. The model revealed a central globular core DUF3456 domain with a saposin-like fold (Supplementary Figure S10) flanked by largely unstructured N- and C-terminal segments (Fig. 5A). The core domain (Fig. 5A, green, orange and cyan) was comprised of eight helical segments (all in orange): K48-G76, D93-D109 (kinked), G118–R121, E128–K139, A157–E172, E174–N183, L189–V198, and S205–A208, with at least one helical turn and a short two-stranded anti-parallel β-sheet (Y110–L112:F122–K124; cyan) connected by various loop segments (green). The structure further included three cystine disulfide bridges (Fig. 5B), in agreement with biochemical evidence,^15^ and featured strong spatial overlap in the core domain with a solved CNPY5 structure (data not shown).^34^ Cleavage of the signal sequence did not result in major changes to the core domain (Supplementary Figure S11).

**Fig. 5:**
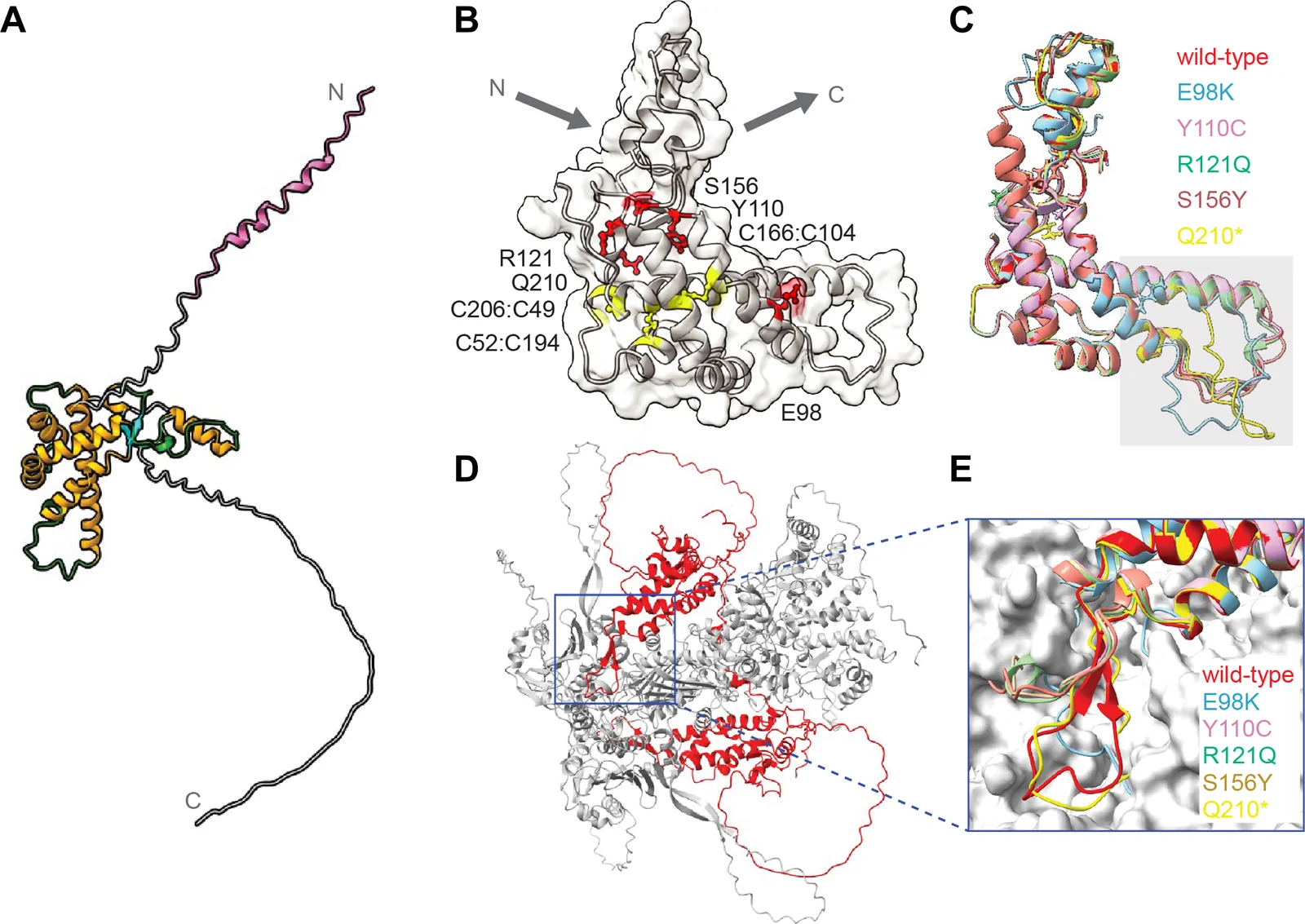
**Structure prediction of CNPY3 wild type and disease-associated variant proteins.** (**A**) AlphaFold prediction of full-length CNPY3 including the largely disordered N- and C-termini with secondary structure elements (helix: orange, β-strands: cyan) of the core domain (loops and turns coloured in green). The N-terminal signal sequence, including a predicted α-helical stretch, is highlighted in violet. N- and C-termini are marked. (**B**) Predicted structure of the CNPY3 core domain. The position and side chain atoms of residues E98, Y110, R121, S156 and Q210 (full-length numbering) mutated in the disease-associated variants are highlighted in red. Disulfide bridges (C49/C206, C52/C194, C104/C166) are indicated in yellow. The entry and exit points of the N- and C-terminus, respectively, are marked with an arrow for orientation. Aspect rotated by −90° via z axis from A. (**C**) Superposition of the CNPY3 variants with the AlphaFold wild type prediction (red). Side chains of the mutated residues are shown. Grey shaded box highlights the area of structural deviations in helices I and II and the intervening loop. (**D**) CNPY3:Grp94 complex prediction. The Grp94 dimer in the CNPY3:Grp94 complex is depicted in grey, the two CNPY3 units in red. Blue rectangle delimits the area of the structure shown in panel E. (**E**) Excerpt (see blue box in D) with superposition of the variants to the wild type prediction (red) highlighting an area of steric clashes with the Grp94 unit for variants E98K, Y110C, R121Q and S156Y. Q210^∗^ exhibits a backbone fold similar to the wild type preventing steric clashes.

All selected missense mutations mapped to the DUF3456 core domain (Fig. 5B, compare with Fig. 4). Structural alignment of wild type and variant structures revealed that E98K and Q210∗ showed significant conformational deviations in the loop between helices I and II (Fig. 5C). In E98K and Q210∗ helix I was predicted to be shortened by one or two turns, whereas Y110C, R121Q and S156Y resembled a helix 1 length and loop orientation similar to the wild type. CNPY3 acts as a co-chaperone of the HSP90 paralogue Grp94, which operates as a dimer.^35^ Accordingly, the CNPY3:Grp94 complex was modelled with a 2:2 stoichiometry (Fig. 5D), where backbone fold predictions of variants E98K, Y110C, R121Q, and S156Y revealed steric clashes with helix E253–L259 in Grp94 (Fig. 5E). By contrast, the shortened helix I of Q210^∗^ resembled the wild type conformation, enabling a compatible dimer formation with the Grp94 unit.

### Protein expression of CNPY3 variants

Eight *CNPY3* variants were selected for functional analysis (Supplementary Data 6). We prioritised missense variants (CNPY3^S92L^, CNPY3^E98K^, CNPY3^Y110C^, CNPY3^R121Q^, CNPY3^G125R^, and CNPY3^S156Y^), as well as one nonsense (CNPY3^Q210^^∗^) and one deletion variant causing a premature termination at the exit of the DUF3456 core domain (CNPY3^N183X^) (Fig. 5B). We reasoned that these variants were likely to produce stable proteins and thus be amenable to experimental analysis. The CNPY3^Q210^∗ and CNPY3^R121Q^ variants occur in compound heterozygosity, making their functional interplay of particular interest. Transient expression in HEK293T cells confirmed protein expression of CNPY3^S92L^, CNPY3^E98K^, CNPY3^Y110C^, CNPY3^G125R^ and CNPY3^Q210^^∗^, and at low levels for CNPY3^R121Q^ and CNPY3^S156Y^ (Fig. 6A). In line with previous findings, CNPY3^N183X^ did not yield a stable polypeptide. In summary, except for CNPY3^N183X^, the variants tested generated stable protein products.

**Fig. 6:**
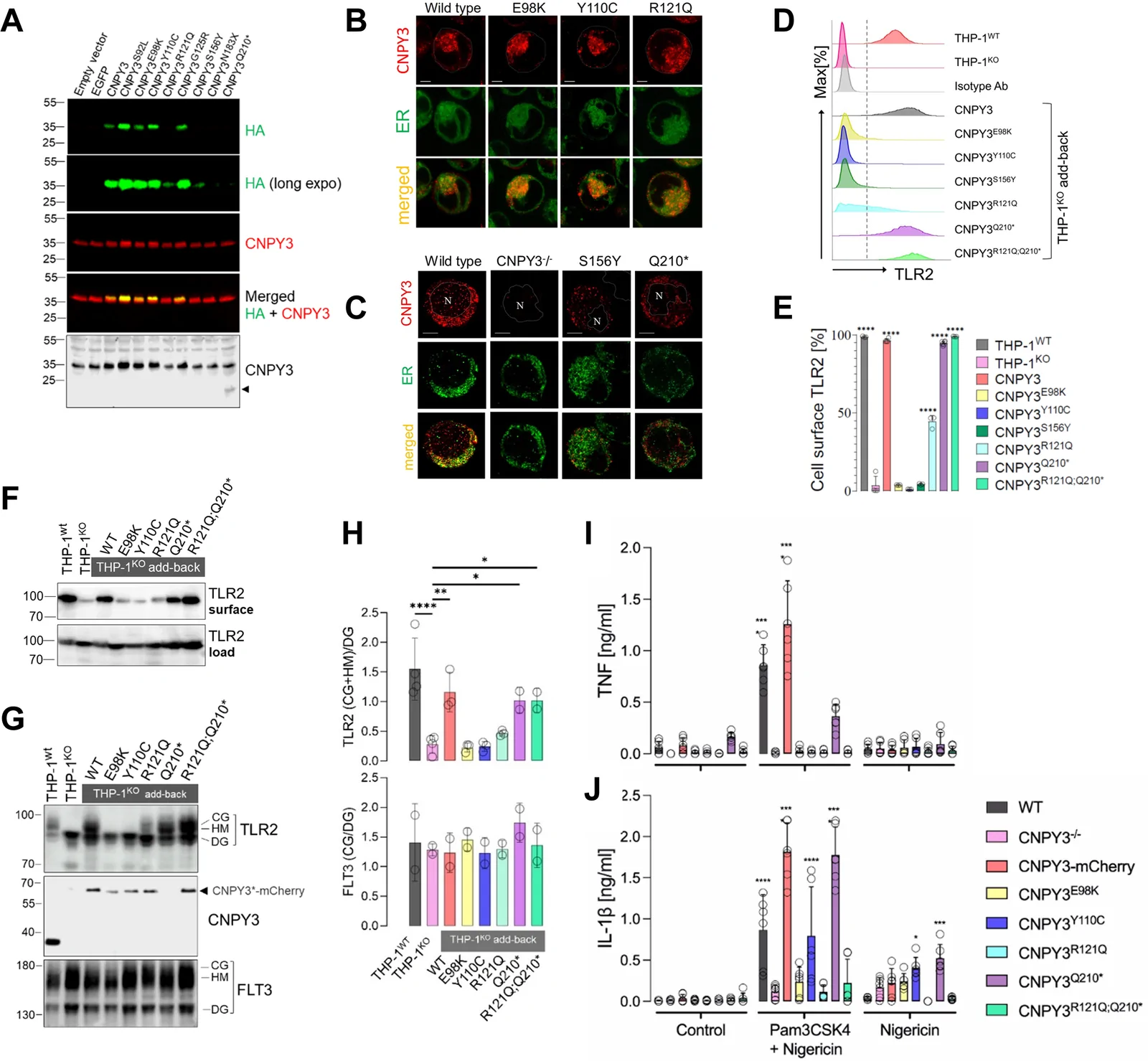
**Functional characterisation of CNPY3 variants.** (**A**) Heterologous CNPY3 variant protein expression in HEK293T cells. The indicated HA-tagged CNPY3 constructs were transiently expressed in HEK293T and visualised by western blotting and immunofluorescent detection with the indicated antibodies. A long exposure is shown to illustrate low level expression of two variants. All tested CNPY3 variants except CNPY3^N183X^ give rise to protein products. A merged image of CNPY3 and HA-tag immunodetections revealed the presence of endogenous CNPY3 in HEK293T cells and the slightly lower mobility of the heterologous variant proteins caused by the appended HA-tag. The CNPY3^Q210∗^ protein was detectable by standard enhanced chemiluminescence detection as shown in the lower panel (marked by arrowhead). See main text for details. (**B**) Live cell imaging of THP-1 cell lines expressing CNPY3 wild type or variants. CNPY3^KO^ cells stably expressing the indicated CNPY3-mCherry variant fusion proteins were stained with a live-cell ER marker and imaged alive by confocal fluorescence microscopy. CNPY3^Q210∗^ could not be visualised as the expression construct retained the premature stop codon to exclude any effects of the C-terminally fused mCherry on the variant function. Cells shown are representative of ≥75% of all cells in the conditions imaged. The perimeter of the cell is traced in the mCherry panels for clarity. Magnification bars: 5 μm. (**C**) Immunocytochemical images of THP-1, CNPY3^KO^, CNPY3^S156Y^ and CNPY3^Q210∗^ cells. Slides were stained against CNPY3 and the endoplasmic reticulum marker Reticulon 4 (ER). Nuclei are silhouetted by a dotted line and marked with N. Magnification bars: 5 μm. (**D**) Wild type THP-1, CNPY3^KO^ and stable add-back cell lines expressing the indicated CNPY3 variants were stained for TLR2 or with an isotype control antibody and processed by flow cytometry. The dotted line marks the threshold gate applied for quantification shown in (**E**). (**E**) Quantification of TLR2 surface levels shown in panel C. One-way ANOVA–Šidak's multiple comparisons (∗∗∗∗ *P* ≤ 0.0001; ns, *P* > 0.05; n = 3–6). (**F**) Biotinylation assay for analysis of TLR2 cell surface expression. The indicated THP-1 cell lines were treated with a membrane-impermeable biotinylation reagent. Cells were lysed, biotinylated proteins collected on streptavidin beads and samples were processed by western blotting for TLR2. (**G**) Electrophoretic mobility shifts document distinct TLR2 glycosylation patterns in CNPY3 variant cells. Cell extracts from the same THP-1 cell lines as before were separated on a gradient SDS-PAGE with high resolving power to highlight mobility shifts of glycosylated TLR2 species. FLT3 was visualised as negative control. For unclear reasons, the CNPY3^Q210∗^ variant was not detectable under these conditions, see text for details. CG, complex glycosylated species; DG, deglycosylated form; HM, high-mannose form. (**H**) Quantification of TLR2 glycosylation in CNPY3 variant lines, expressed as the (CG + HM)/DG ratio. One-way ANOVA–Šidak's multiple comparisons (∗∗∗∗ *P* ≤ 0.0001, ∗ *P* < 0.05; n = 2–4). (**I**, **J**) CNPY3 regulates TLR2 signalling and inflammasome activation. Engineered THP-1 cell lines were challenged with the TLR2 agonist Pam3CSK4 and the inflammasome activator nigericin individually or sequentially as indicated followed by determination of TNF (**I**) or IL-1β (**J**) secretion by ELISA. The same variant/column colour code was used throughout all figure panels. One-way ANOVA–Šidak's multiple comparisons (∗∗∗ *P* ≤ 0.001, ∗∗∗∗ *P* ≤ 0.0001; ns, *P* > 0.05; n = 6).

### *CNPY3* variants exert variable effects on TLR2 maturation and trafficking

Given the role of CNPY3 in TLR chaperoning and inflammasome regulation in myeloid cells, we employed gene-edited THP-1 monocytes lacking CNPY3 expression (CNPY3^KO^) to generate add-back lines by lentiviral transduction of wild type or disease-associated CNPY3 variants. To characterise the compound heterozygous variants CNPY3^R121Q^ and CNPY3^Q210^^∗^ (F20), we generated cell lines expressing each variant individually and in combination. For unknown reasons, the CNPY3^S156Y^ variant in fusion with mCherry did not yield viable cell lines, precluding its analysis. To circumvent this limitation, we resorted to an alternative, viable THP-1 cell line expressing CNPY3^S156Y^ tagged with TurboID, a cell line designed for proteomic screening.^28^ Western blotting confirmed variant protein expression (see below Fig. 6G) while live-cell imaging and immunocytochemical analysis showed their correct localisation to the ER (Fig. 6B and C). Of note, CNPY3^Q210^^∗^ and CNPY3^S156Y^-TurboID were only detectable by immunocytochemistry because both lacked the C-terminal mCherry fusion (CNPY3^Q210^^∗^ due to its premature stop codon) (Fig. 6C). Assessment of TLR2 surface expression by flow cytometry confirmed the loss of TLR2 surface expression in CNPY3^KO^ cells and its restoration upon wild type CNPY3 add-back (Fig. 6D and E, gating strategy shown in Supplementary Figure S12). In line with their presumptive loss of function, variants CNPY3^E98K^, CNPY3^Y110C^ and CNPY3^S156Y^ failed to restore TLR2 trafficking to the plasma membrane, reminiscent of the previously reported case of CNPY3^N183X^.^7^ Unexpectedly, the compound heterozygous variants CNPY3^Q210^^∗^ and, to a lesser extent, CNPY3^R121Q^ restored TLR2 levels at the plasma membrane (PM) if expressed individually or together (see Fig. 6D and E). Cell surface biotinylation using a membrane-impermeable reagent to distinguish between PM-exposed and intracellular TLR2 confirmed these findings (Fig. 6F). Since cell surface exposure of receptors is preceded by their complex glycosylation in the ER and Golgi, we carefully analysed the electrophoretic behaviour of TLR2, as receptor glycosylation causes characteristic mobility shifts. Western blotting for TLR2 confirmed the requirement for CNPY3 in efficient glycosylation, yielding a glycosylation pattern of the variant cell lines that was fully consistent with the TLR2 cell surface analysis (Fig. 6G, quantification in Fig. 6H). Glycosylation of the receptor tyrosine kinase FLT3 was not affected, proving the specificity of CNPY3 for TLRs. Glycosidase assays using Endo H, which cleaves ER-resident mannose-rich but not complex glycosylated tree structures in the Golgi^36^^,^^37^ showed that loss of CNPY3 prevented complex glycosylation of TLR2 at a pre-Golgi step (Supplementary Figure S13). Interestingly, the electrophoretic mobility of CNPY3 was also altered by glycosidase treatment, which suggests that CNPY3 itself may undergo transient glycosylation in the ER (Supplementary Figure 13A). In the course of these experiments, we further noticed that CNPY3^Q210^^∗^ was not detectable by western blotting of THP-1 cell extracts, despite being detectable upon heterologous expression (Fig. 6A), by immunofluorescence (Fig. 6C), via LC-MS analysis of cell extracts and CNPY3 immunoprecipitates (PRIDE dataset PXD077397) and despite the clear rescue effect on TLR2 trafficking. The absence of a signal may be attributable to low protein abundance or protein modifications that prevent the detection of the CNPY3^Q210^^∗^ protein by western blotting in THP-1 cells. Nevertheless, the above observations indicated that the compound heterozygous genotype of individual F20:II.4 did not compromise TLR2 folding and maturation, a finding that appeared inconsistent with TLR dysfunction as a cause of CNPY3-DEE.

### All disease-associated variants prevent TLR signalling while CNPY3^Y110C^ and CNPY3^Q210^^∗^ provide inflammasome priming

To confirm the CNPY3 variant effects on TLR function, we measured cytokine release in response to TLR2 stimulation. Analysis of TNF release in response to Pam3CSK4 yielded a different picture to the previous assays, as all disease-associated *CNPY3* genotypes failed to support TLR2 signalling (Fig. 6I). The TLR chaperoning function of CNPY3 is also essential for downstream signalling to the NLRP3 inflammasome.^5^ Accordingly, inflammasome activation induced by nigericin in Pam3CSK4-primed THP-1 macrophages was lost in the CNPY3-deficient background and rescued by adding back wild type CNPY3 (Fig. 6J). Re-expression of CNPY3^E98K^ and CNPY3^R121Q^ did not support inflammasome activation. However, CNPY3^Q210^^∗^, the variant that sustained TLR processing, provided robust inflammasome activation, which was reverted by co-expression of its compound allele CNPY3^R121Q^, evidencing a strong non-linear interallelic effect. Interestingly, CNPY3^Y110C^ also supported marked inflammasome activation, even though this variant prohibited TLR2 surface expression and signalling (Fig. 6D–H). In summary, CNPY3^Q210^^∗^ and CNPY3^Y110C^ produced a priming signal independent of TLR2 for the activation of the NLRP3 inflammasome, as reflected also by the increased release of IL-1β in response to nigericin in the basal state (Fig. 6J, right panel).

## Discussion

This study presents a comprehensive clinical and genetic characterisation of individuals with biallelic *CNPY3* variants, significantly expanding the spectrum of a disorder that is strikingly early in onset and severe in nature, and delivering functional insights into molecular mechanisms of TLR trafficking and inflammasome activation. Core clinical features included global developmental delay and intellectual disability, epilepsy, encephalopathy, and spasticity. Microcephaly was also frequent, and status epilepticus, apnoeas, stridor and premature deaths were notable additional features. Growth faltering emerged as an integral component of the phenotype, consistent with observations from animal models.^3^^,^^11^ Across individuals in our cohort, a parallel reduction in weight, length, and head circumference was observed. Notably, the absence of relative head sparing suggests that the observed growth deficits cannot be fully explained by secondary effects of central nervous system dysfunction, such as feeding difficulties. Instead, the overall growth pattern may indicate an intrinsic growth disturbance associated with CNPY3 dysfunction. Neuroimaging findings indicated structural brain involvement, including hippocampal abnormalities. Unlike the original report describing hippocampal malrotation,^3^ we did not observe incomplete hippocampal inversion—the currently preferred term—as a consistent feature in the available imaging.

Genetic analyses identified multiple variants, predominantly in homozygous state. According to ACMG/AMP criteria, most variants were classified as variants of uncertain significance, whereas only a minority met criteria for likely pathogenic or pathogenic classification. Variant interpretation relied primarily on population data, computational prediction of variant consequence, and segregation data, which were available for most patients and provided additional supporting evidence. However, given the autosomal recessive inheritance pattern and the presence of consanguineous families in the cohort, segregation evidence should be interpreted with caution. Accordingly, the predominance of variants of uncertain significance should not be viewed as evidence against their clinical relevance, but rather as reflecting the current evidentiary threshold required for confident classification under the ACMG/AMP framework. These variants remain important candidates for future reclassification as additional clinical and functional evidence becomes available. Shared extended haplotypes were observed for two distinct recurring variants in families from two different geographic regions, suggesting possible founder effects. Truncating variants were associated with more severe phenotypes within the cohort, whereas missense variants accounted for most of the observed phenotypic variability, highlighting their heterogeneous functional consequences.

The variability observed among individuals with identical genotypes, together with the greater consistency within families compared to across individuals sharing the same genotype, further supports a role for shared background factors in modulating phenotypic expression. Overall, these findings support a hierarchical model in which variant type determines the underlying level of disease severity, while additional modifiers shape the final phenotype.

In-frame deletions or insertions within the poly-leucine stretch of the ER signal sequence correlated with more severe phenotypes, consistent with impaired ER transport. Truncating variants were linked to the highest mortality (Supplementary Figure S14).

The clinical variability was mirrored by the diverse outcome observed in our functional studies. Across all disease-associated genotypes, TLR2 signalling, as assessed by TNF release, was consistently abolished, suggesting defective TLR signalling as a plausible contributor to DEE. By contrast, the uniform loss of downstream TLR2 signalling did not translate to TLR2 surface expression and TLR glycosylation. This discrepancy suggests additional, as yet unidentified functions of CNPY3, such as chaperoning of a TLR accessory protein or a more global role in cytokine secretion. Notably, the only variant that fully rescued TLR2 surface expression, CNPY3^Q210^^∗^ (c.628C>T), was also the only variant predicted to retain an intact core domain fold (Fig. 5E). These findings indicate that disease-associated missense variants induce conformational changes in the core domain that preclude TLR2 chaperoning, with the observed consequences on glycosylation and PM exposure. Based on these findings, we postulate that an intact DUF3456 domain fold is necessary to maintain TLR maturation and trafficking to the PM.

The DUF3456 domain, also listed as WE or SAPLIP domain in InterPro, is conserved within the CNPY family,^38^ and is also present in the unrelated ER chaperones CRELD1, CRELD2, and pERp1.^34^^,^^39^^,^^40^ This domain reportedly exhibits a protein disulfide isomerase (PDI) fold involved in ER-based disulfide bond formation. However, functional PDI activity in CNPY members has been questioned.^34^ Of note, pathogenic recessive *CRELD1* variants are associated with a neurodevelopmental disorder featuring early-onset epilepsy, global developmental delay, and premature death in childhood.^39^, ^40^, ^41^ This disorder shows phenotypic similarities with CNPY3-DEE and shares an ER chaperoning function of the respective disease-associated gene products.

Our findings indicate that TLR trafficking to the plasma membrane, TLR signalling, and inflammasome activation represent separable functions, likely mediated by distinct CNPY3 domains, as some variants were able to prime the inflammasome machinery independently of upstream TLR signalling. Notably, all individuals (F15-17) carrying the inflammasome-priming variants CNPY3^Y110C^ (c.329A>G) had high PSS values (14, 18, and 19) and died before the age of two years. This aberrant inflammasome activation mechanism could pose a potential link to neuroinflammation and in consequence, epileptogenesis. Accordingly, comprehensive analyses of IL-1β, IL-18 and other pro-inflammatory cytokines in serum and CSF of affected individuals should be conducted in future studies.

The variant CNPY3^R121Q^ (c.362G>A) also warrants special mention, as it is associated with moderate clinical severity (PSS 7 and 8, respectively) in a homozygous state, but with a severe phenotype (PSS 18) when present in compound heterozygosity with CNPY3^Q210^^∗^ (c.628C>T). In our cell model, this compound heterozygous background resulted in non-additive effects on TLR signalling and inflammasome activation, despite preserved TLR2 surface expression (Fig. 6D and E, I and J). We speculate that these variants may act in a complementary manner to produce a pathological phenotype, potentially through cooperative interactions within a CNPY3 dimer.^42^ Finally, the differential regulation of TLR trafficking and signalling by CNPY3 variants suggests additional regulatory mechanisms of TLRs that may be relevant for innate immunity beyond DEE.

Several limitations should be considered when interpreting these findings. The non-population-based recruitment strategy and retrospective multicentre design may have introduced ascertainment bias, including enrichment for clinically severe presentations and individuals with access to genomic testing, while statistical power for genotype-specific analyses remained limited. The retrospective design also resulted in variable data completeness across centres. Although the observed pattern of missingness was broadly compatible with a missing-at-random mechanism, this assumption cannot be formally verified. In addition, the in vitro analyses were restricted to selected variants and may not fully capture the biological complexity of *CNPY3* dysfunction. Nevertheless, our findings indicate that defects in CNPY3 are associated with abnormal neurodevelopment and epilepsy, involving TLR signalling, inflammasome regulation, and possibly growth control. This aligns with the current concept that innate immune pathways contribute to epileptogenesis through cytokine release, altered synaptic transmission, and neuronal hyperexcitability.^43^ In addition, intracellular immune signalling is shaped by subcellular organisation of pathway components, with compartment-specific interactions.^44^, ^45^, ^46^ In this context, the uncoupling of TLR trafficking and signalling observed here suggests that disruption of intracellular receptor routing is associated with altered immune responses, despite preserved receptor expression.

DEEs are now understood as disorders resulting from a complex interplay of genetic, inflammatory, and neurodegenerative processes.^47^ Conditions such as autoimmune encephalitis, febrile infection-related epilepsy syndrome (FIRES), and Aicardi–Goutières syndrome, primarily involve downstream effector pathways, including cytokine-dependent hyperexcitability and interferon-mediated activation. In contrast, our findings suggest a disturbance in TLR maturation and trafficking, pointing to a receptor-proximal defect that may impact multiple downstream signalling pathways. While TLR signalling influences brain development,^48^ TLR2 or TLR4 knockout in mice does not reproduce the severe phenotype of CNPY3 dysfunction in mice or humans.^49^^,^^50^ This suggests that additional substrates and pathways dependent on CNPY3 are likely to contribute to disease pathogenesis.

In summary, this study documents important clinical and functional features that improve our understanding of CNPY3-DEE and its impact on cellular immunity. The broad phenotypic and functional spectrum suggests that disease-associated variants have graded and multivariable effects on CNPY3 function and disease severity. Further functional and in vivo validation studies are required to elucidate the precise molecular mechanisms underlying CNPY3-related pathology and to identify potential therapeutic targets, including as yet elusive CNPY3 client proteins in immune and neuronal cells.

## Contributors

VGa, MGr, RM, SE, SSt, IR, and RAH contributed to the study methodology.

RAH and IR conceptualised the study.

VGa, MGr, RM, SE, SSt, DBe, CT, MGh, OO, AD, MW, SN, EB, RK, SB, IP, SBW, JRA, TS, AC, CSH, IM, ARoPa, EW, JV, CMM, RAJ, MRA, NA, RAz, HG, NC, MZ, GSh, PN, FBA, AFa, SNH, ZI, SK, MT, EA, SM, FR, VS, MGris, CR, CAlv, LJ, DC, CAB, CAlA, MA, MU, BOJ, SH, VJ, BN, FA, NE, EPP, LRP, RSM, DBa, LL, AF, MSZ, GSv, AMu, AB, KS, UG, KK, HN, ARePo, MSM, TTS, MMu, HAR, GO, ARa, MMi, AMa, VKG, VMS, RMA, ISA, BA, KB, MB, IR, and RAH contributed to investigation and data acquisition.

VGa, MGr, RM, SE, SSt, HdV, MW, IR, and RAH contributed to data analysis and interpretation.

VGa, IR, and RAH wrote the first draft of the manuscript.

MGr, RM, SE, SSt, MGh, SB, NA, EPP, RSM, GO, ARa, FSA, PH, HH, and TBH critically reviewed and edited the manuscript.

HH, TBH, IR, and RAH supervised the study.

All authors have read and approved the final version of the manuscript.

IR and RAH have accessed and verified the underlying data.

## Data sharing statement

DNA sequence datasets have been generated and contributed by different study sites and have not generally been deposited in a public repository due to varying local consent regulations. Selected datasets will be made available by the corresponding author on reasonable request. Mass spectrometry proteomics data have been deposited to the ProteomeXchange Consortium via the PRIDE repository with the dataset identifier PXD077397 (https://www.ebi.ac.uk/pride/archive/projects/PXD077397).

## Declaration of generative AI and AI-assisted technologies in the manuscript preparation process

During the preparation of this work, the authors used ChatGPT (OpenAI) for language and grammar checking. After using this tool, the authors reviewed and edited the content as needed and take full responsibility for the content of the published article.

## Declaration of interests

Hafiz Muhammad Mustafa was employed by SMS Pakistan; Gabriela Oprea and Aboufazl Rad are currently employed by Arcensus GmbH; Bader Alhaddad, Khadijah Bakur and Fowzan S. Alkuraya are employed by Lifera Omics; all are commercial providers of genetic diagnostic testing. Rikke Steensbjerre Møller has received consulting fees from UCB, Immedica, Orion, and payment or honoraria for lectures and presentations from EISAI, ANGELINI, UCB, Jazz Pharmaceuticals, Orion, Saniona. The remaining authors declare no conflict of interest.

## Appendix A Supplementary data

Supplementary Material – Figures, Case Reports, and Methods

Supplementary Data 1 Individual-Level Data

Supplementary Data 2 Rates of Missing Data

Supplementary Data 3 PSS and Cluster Data

Supplementary Data 4 Detailed Genetic Data

Supplementary Data 5 Haplotype Analysis

Supplementary Data 6 Variants Analysed
