# Supplementary material for: CNPY3 variants associated with a recessive developmental and epileptic encephalopathy affect inflammasome and TLR function
Source: eBioMedicine. 2026 Oct 14;133:106506. doi: 10.1016/j.ebiom.2026.106506 (PMC13694807; doi:10.1016/j.ebiom.2026.106506)
Supplement: Supplementary file 1 [file mmc1.pdf]

Supplementary Material

for

Vera Garloff et al.

CNPY3 variants associated with a recessive developmental and epileptic encephalopathy affect inflammasome and TLR function

eBioMedicine

Contents

		Page
Supplementary Figure S1.	Parental countries of origin in the study cohort	1
Supplementary Figure S2.	Pregnancy-related complications	2
Supplementary Figure S3.	Patient growth at birth and at last examination	3
Supplementary Figure S4.	Growth charts at the time of last examination	4
Supplementary Figure S5.	HPO term-based clustering of clinical phenotypes	5
Supplementary Figure S6.	Sensitivity analyses of phenotype severity score comparisons	6-7
Supplementary Figure S7.	Pedigrees and associated variants	8
Supplementary Figure S8.	Aberrant transcript analysis of the c.496-3_496-2del intronic variant	9
Supplementary Figure S9.	Verification of variants in the low-complexity region	10
Supplementary Figure S10.	CNPY3 structure predictions	11
Supplementary Figure S11.	AlphaFold models of full-length versus signal sequence-deleted CNPY3	12
Supplementary Figure S12.	Gating strategy for flow cytometric analysis of TLR2 surface expression in THP-1 cell lines	13
Supplementary Figure S13.	Complex glycosylation of TLR2 in CNPY3 variant monocyte lines	14
Supplementary Figure S14.	Variant consequence and mortality	15
Supplementary Case Reports		16-35
Supplementary Methods:	Phenotype Severity Score — detailed scoring manual	36-38

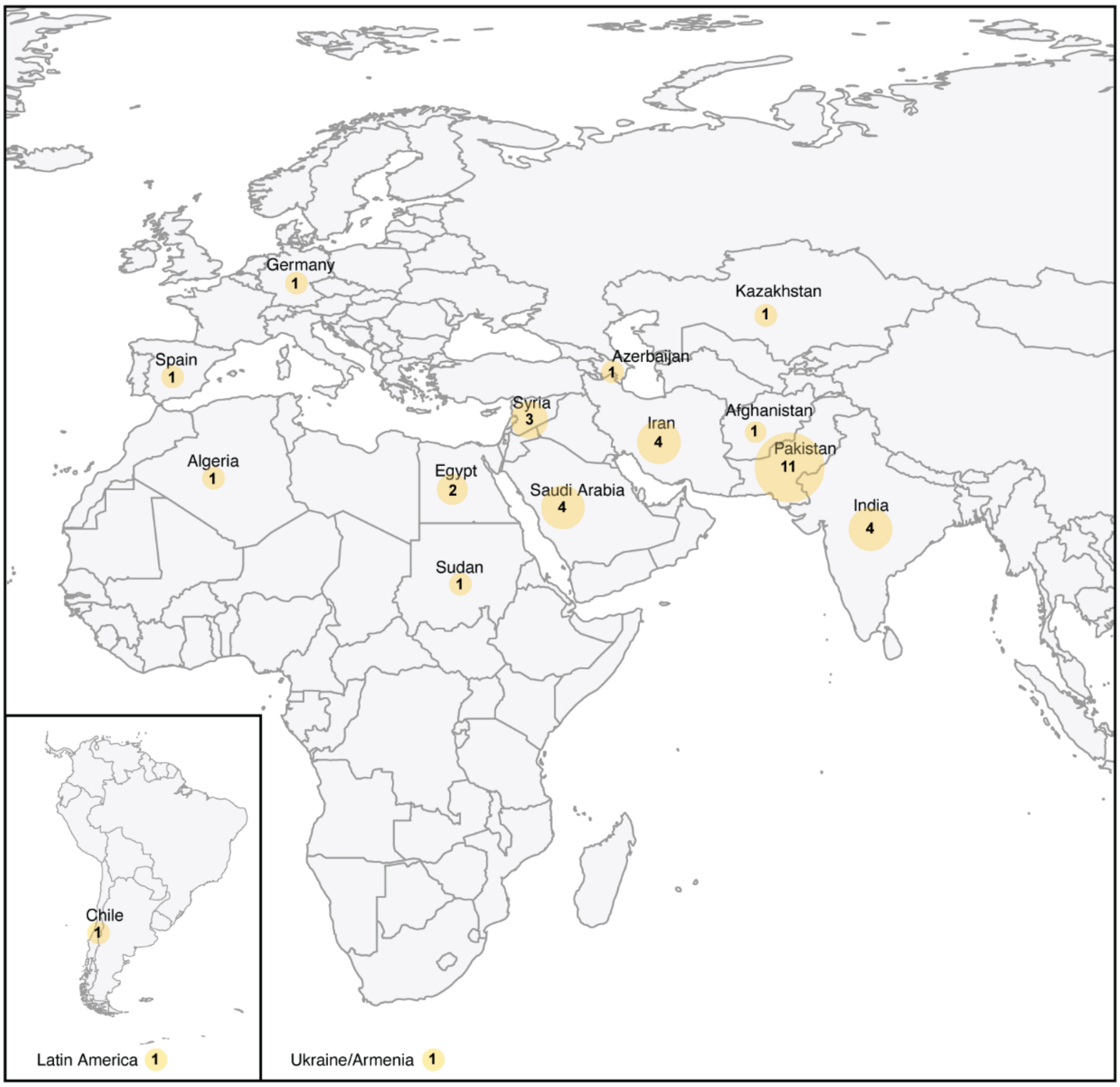

Supplementary Figure S1. Parental countries of origin in the study cohort

Map showing the geographic origin of the 38 unrelated families included in the cohort of biallelic *CNPY3*-associated disease. Proportional circles indicate the number of families originating from each country. Geographic origin was assigned based on the reported country of family origin.

Disclaimer: The boundaries and names shown on this map do not imply official endorsement or acceptance by the authors or publisher.

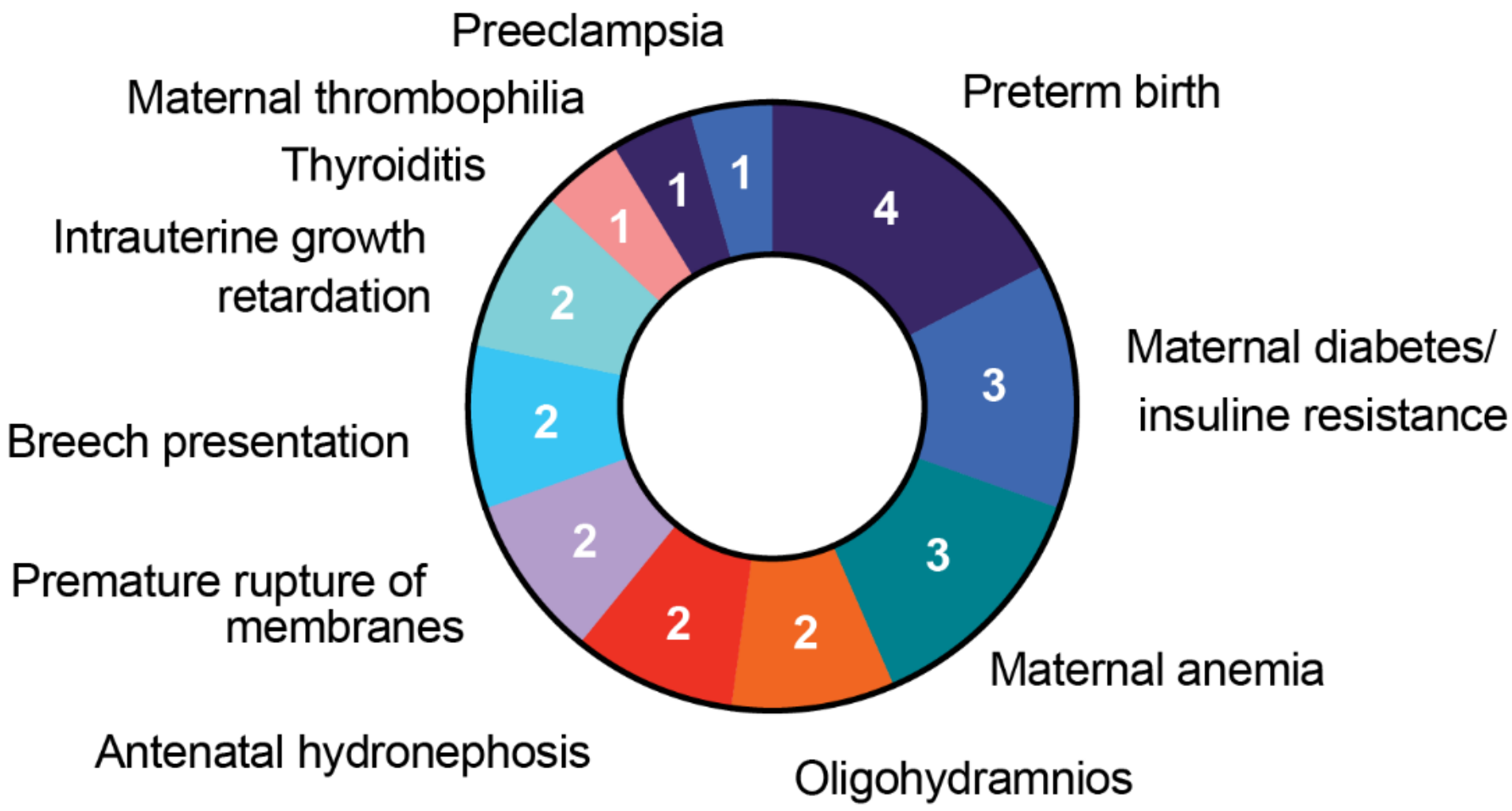

Supplementary Figure S2. Pregnancy-related complications

Chart depicting individual pregnancy-related complications and their frequencies. Overall, complications occurred in 33.3% (16/48) of pregnancies, with no unusual findings observed. Foetal CNS anomalies in F31:II.10 (hypoplastic corpus callosum, absent cavum septi pellucidi, and bilateral cystic periventricular white-matter changes) were not included, as they were likely attributable to the underlying condition rather than to general pregnancy complications.

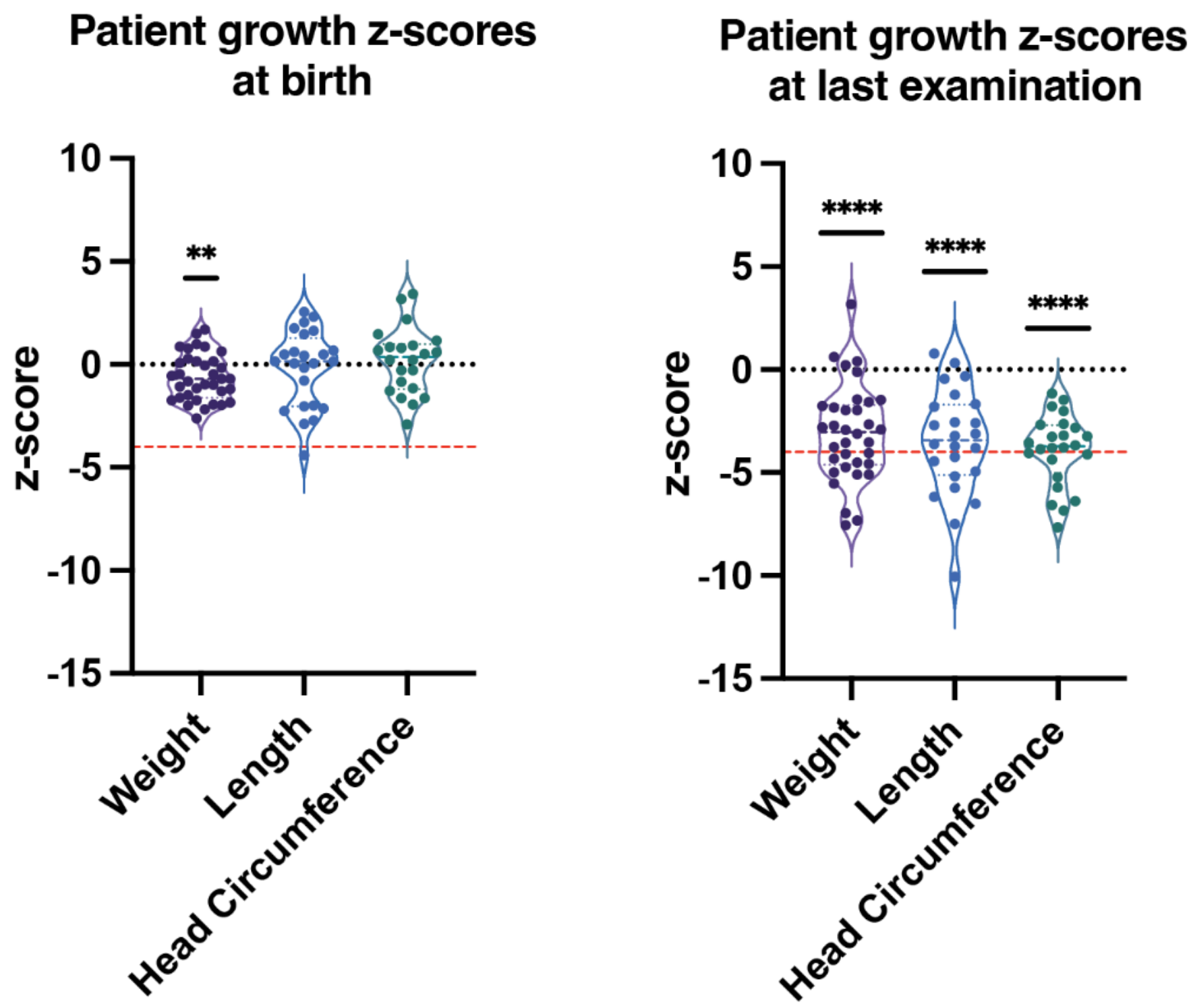

Supplementary Figure S3. Patient growth at birth and at last examination

Growth parameters - length, weight, and head circumference - were converted into age-independent z-scores using reference data from the World Health Organization (WHO) and the INTERGROWTH-21st Project. Growth parameters at birth were calculated with the R package gigs (using the INTERGROWTH-21st data set), accounting for variations in gestational age. For assessments from 3 months, z-scores were calculated using WHO growth standards and the R packages anthro and anthroplus. Calculations for preterm patients were based on age corrected for gestational age at birth until 2 years of chronological age, to account for prematurity and ensure accurate growth evaluation. Violin plots show the distribution of values; width reflects data density, the dashed line indicates the median, and the pointed lines represent the interquartile range. The black dotted line indicates the reference z-score of 0 used for the one-sample t-test. The red dashed line marks a z-score of –4. Statistical analysis (one-sample t-test) was performed with GraphPad Prism 10.5.0.
P-values: ** $P < 0.01$; **** $P < 0.0001$.

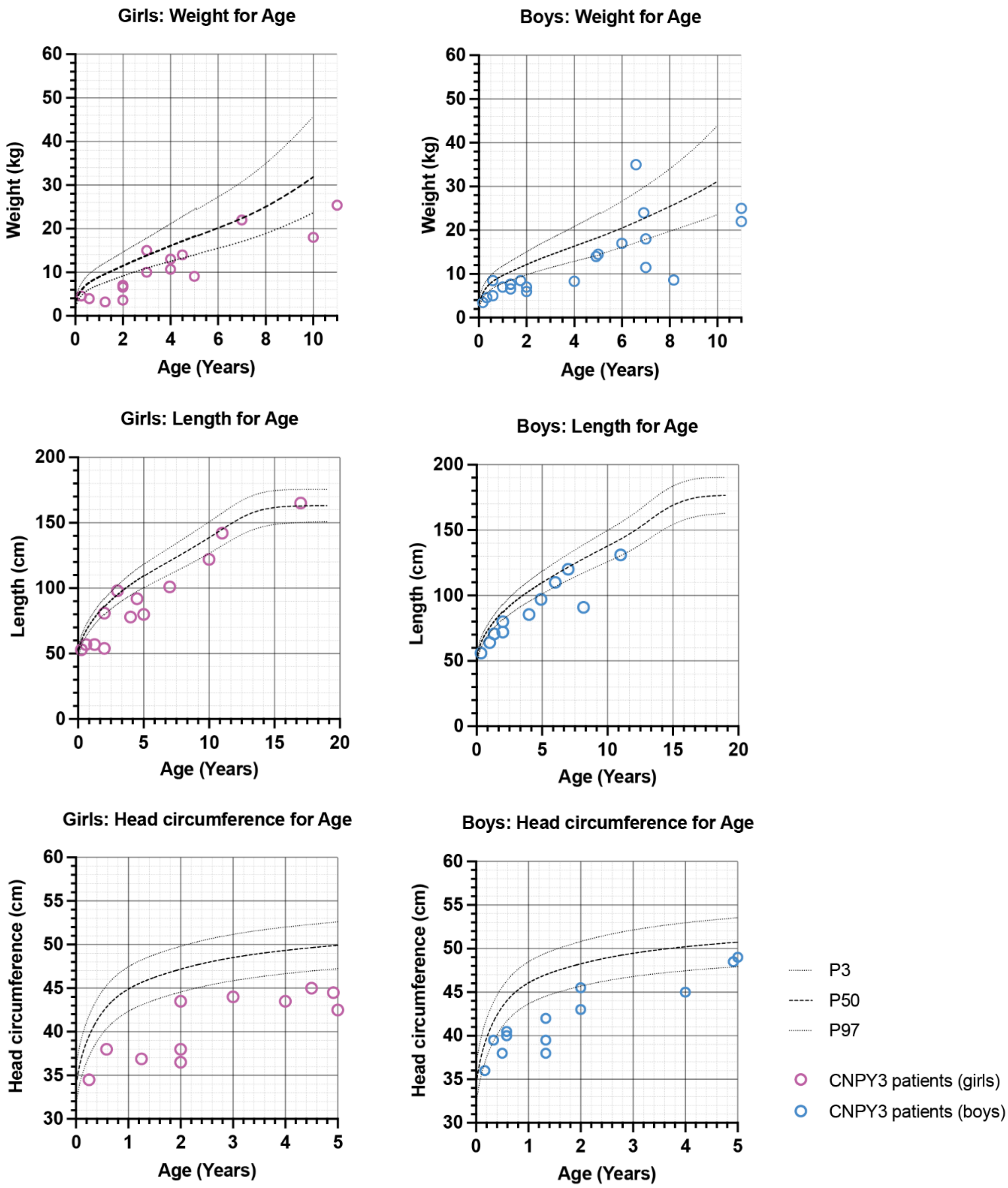

Supplementary Figure S4. Growth charts at the time of last examination

Patient weight, length, and head circumference by age are shown separately for girls and boys and were plotted against WHO growth reference percentiles (P3, P50, P97) using GraphPad Prism 10.5.0.

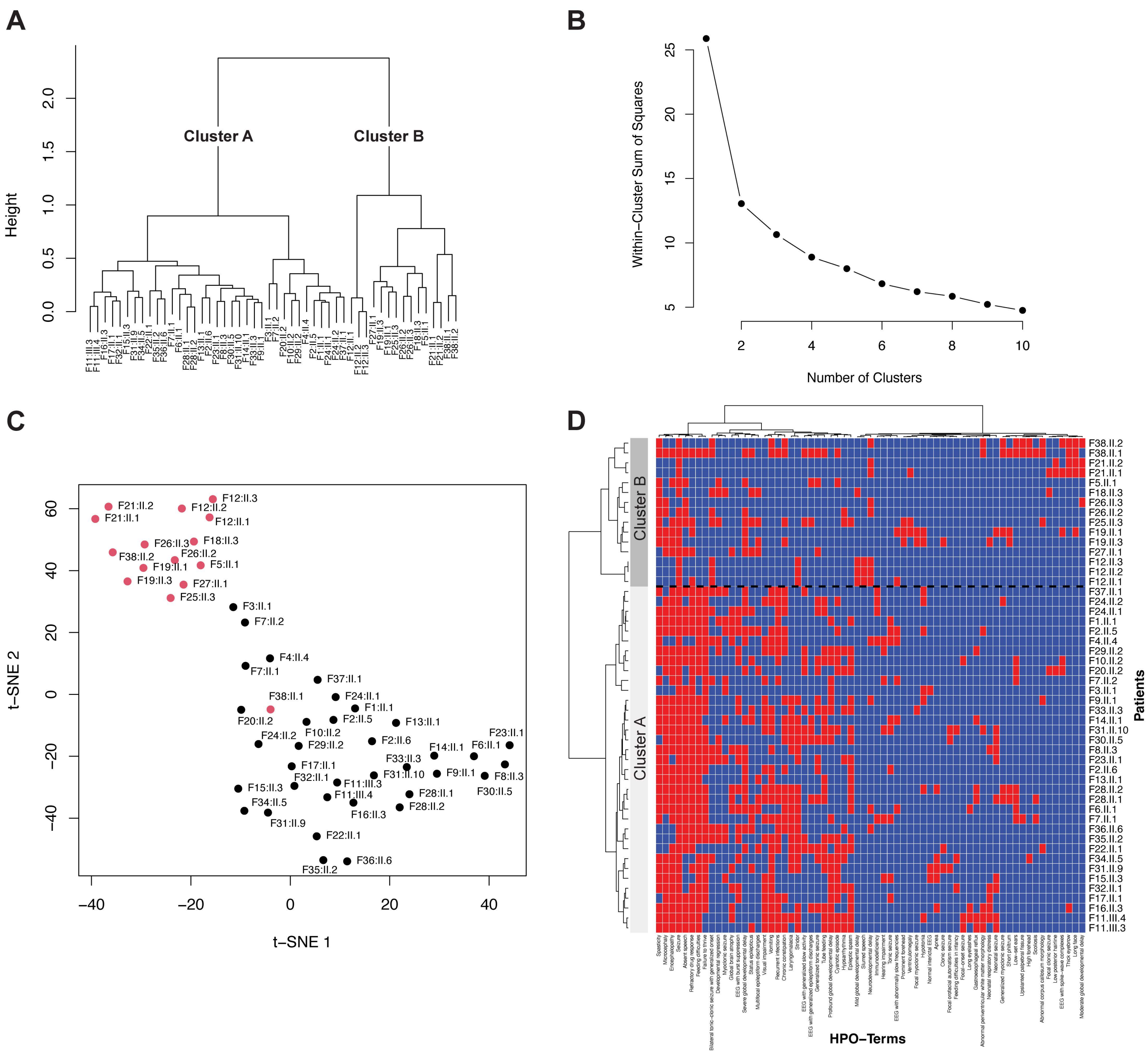

Supplementary Figure S5. HPO term-based clustering of clinical phenotypes

Clinical phenotypes were recorded by the attending physicians and mapped to Human Phenotype Ontology (HPO) terms by independent paediatricians. In total, 184 HPO terms were assigned; the full list is provided in Supplementary Data 1. For the cluster analysis, only HPO terms that were identified in at least three patients of the cohort were included. This resulted in a final list of 65 HPO terms. HPO ontology data were retrieved from purl.obolibrary.org using the R package *ontologyIndex*. Phenotypic similarity between patients was computed using the Lin method implemented in *ontologySimilarity*.

(**A**) Hierarchical clustering using Ward.D linkage (*hclust*) yielded two informative clusters (Cluster A and B). (**B**) The optimal number of clusters was assessed using an elbow plot of within-cluster sum of squares (WCSS), likewise supporting two informative clusters. (**C**) t-SNE visualisation of phenotypic similarity using the *Rtsne* package (perplexity = 10) independently confirmed the clustering pattern (black dots = Cluster A; red dots = Cluster B). (**D**) Heatmap of phenotypic similarity generated with *heatmap.2* (*gplots*), with individuals and features according to hierarchical clustering.

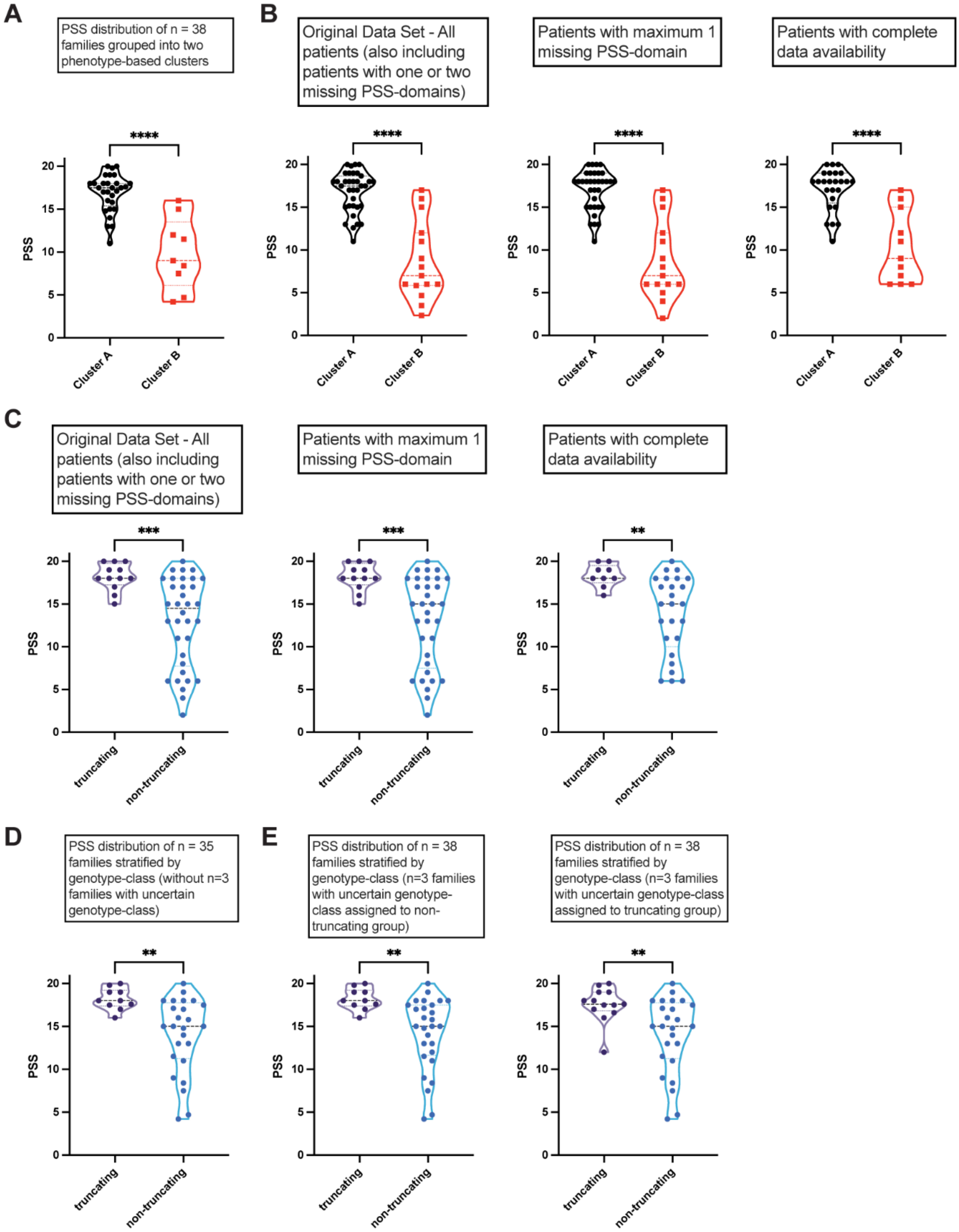

Supplementary Figure S6. Sensitivity analyses of phenotype severity score comparisons

(A) A family-level sensitivity analysis was performed to assess the robustness of the observed differences in phenotype severity scores (PSS) between the two HPO-derived patient clusters (Cluster A vs. Cluster B). To account for familial clustering, each family contributed a single value, defined as the median PSS of all affected individuals within that family. Family-level PSS values were compared using the Mann–Whitney U test. The differences remained significant at the family level. **(B)** Sensitivity analyses addressing incomplete PSS domain data confirmed the robustness of the observed differences in PSS between Cluster A and Cluster B. Patients with incomplete PSS domain data were sequentially excluded (maximum one missing domain; complete data only), and statistical significance was assessed using the Mann–Whitney U test. **(C)** Sensitivity analyses addressing incomplete PSS domain data confirmed the robustness of the observed differences in PSS between truncating

and non-truncating genotype groups. Patients with incomplete PSS domain data were sequentially excluded (maximum one missing domain; complete data only), and statistical significance was assessed using the Mann–Whitney U test. **(D)** A family-level sensitivity analysis was performed to assess the robustness of the observed differences in PSS between truncating and non-truncating genotype groups. To account for familial clustering, each family contributed a single value, defined as the median PSS of all affected individuals within that family. Family-level PSS values were compared using the Mann–Whitney U test. The differences remained significant at the family level. **(E)** An additional sensitivity analysis was performed by reassigning the three families carrying variants with uncertain genotype classification first to the truncating group and then to the non-truncating group. In both scenarios, the differences remained significant in the Mann–Whitney U test, demonstrating that the observed differences were robust to variant classification.

P-values: **** $P \leq 0{\cdot}0001$; *** $P \leq 0{\cdot}001$; ** $P \leq 0{\cdot}01$

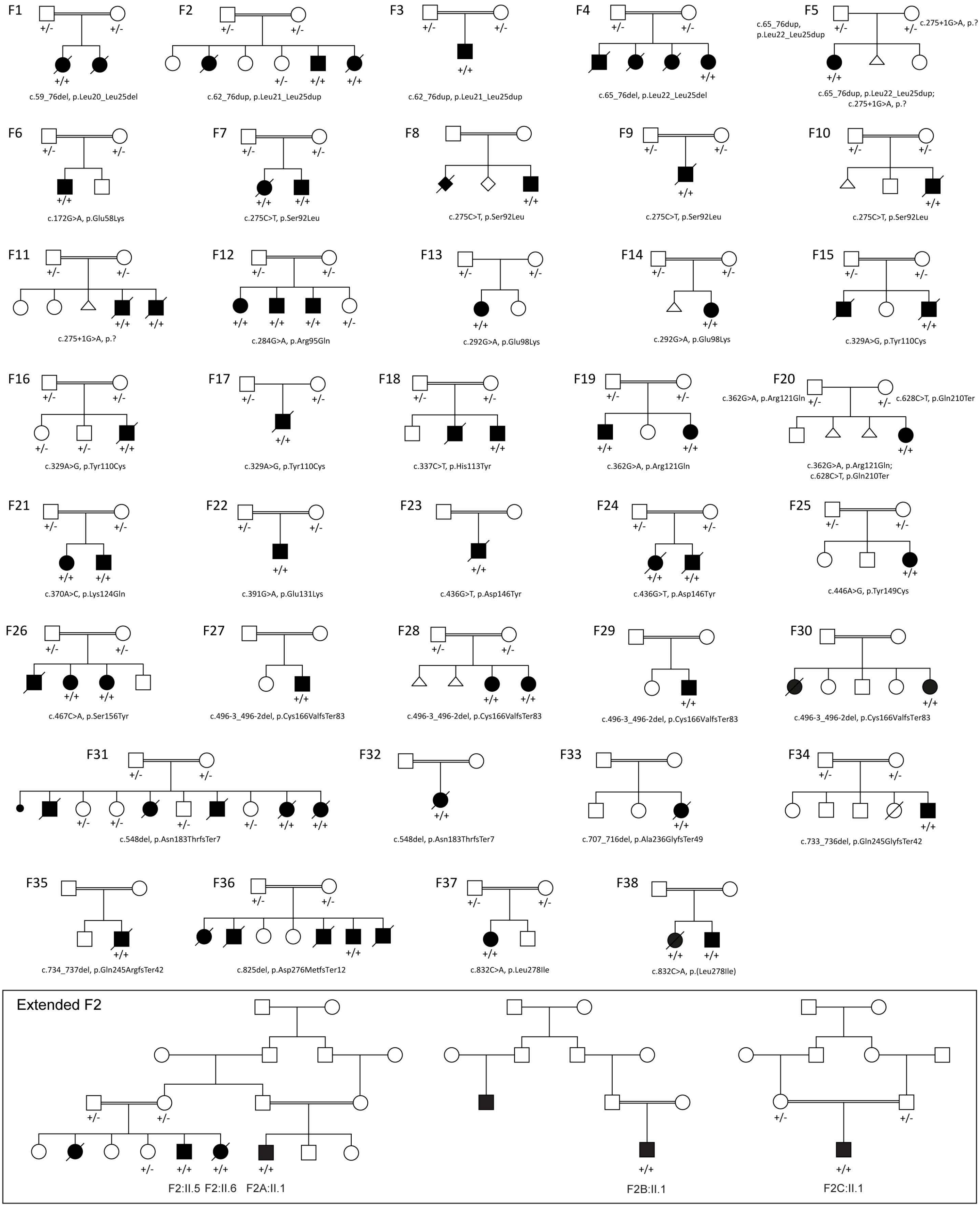

Supplementary Figure S7. Pedigrees and associated variants

Pedigrees of 38 families with biallelic CNPY3 variants, indicating the carrier status (+/+, homozygous or compound heterozygous; +/−, heterozygous) of all affected (closed symbols) and unaffected (open symbols) individuals available for genetic testing. The extended family F2 is shown in the boxed area at the bottom.

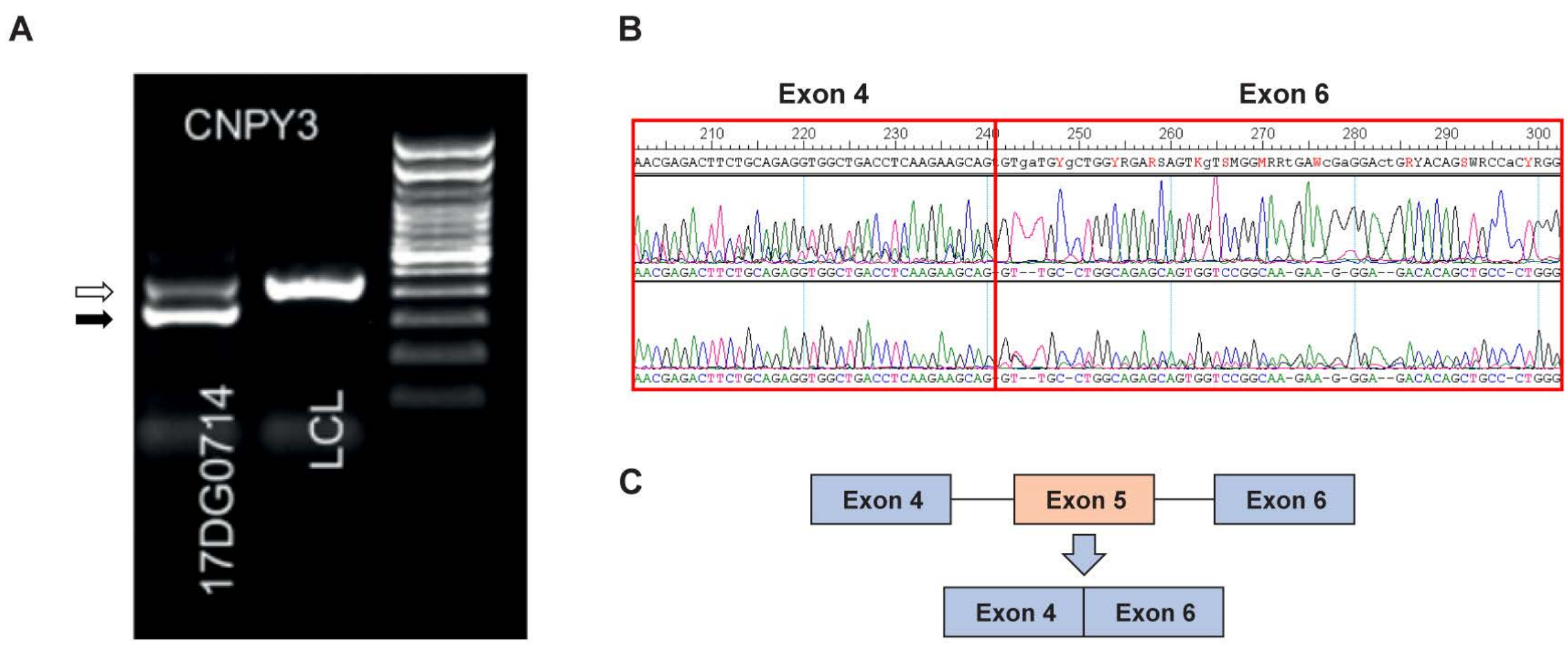

Supplementary Figure S8. Aberrant transcript analysis of the c.496-3_496-2del intronic variant

RT-PCR aberrant transcript analysis of the intronic variant c.496-3_496-2del (families F27-F30), classified as pathogenic (PVS1 and PM2 Supporting; LOFTEE score: HC; PP1). (**A**) RT-PCR agarose gel electrophoresis showing the wild-type band in LCL control cells and two bands in patient sample 17DG0714 (F28:II.1). The upper band (white arrow) corresponds to the wild-type transcript; the lower band (black arrow) corresponds to the aberrant transcript. (**B**) Chromatogram from Sanger sequencing after cloning and sequencing of both bands, showing skipping of exon 5 in the patient sample. (**C**) Schematic representation of complete exon 5 skipping.

A

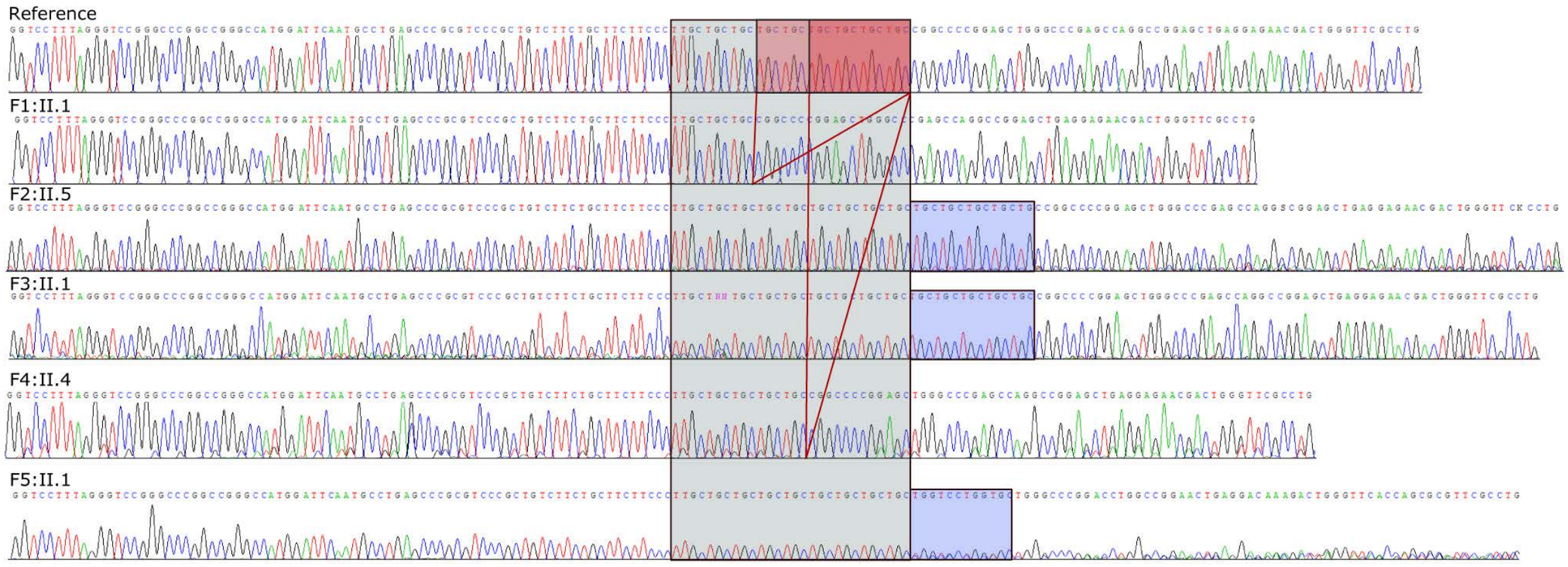

B

```
Reference     GGTCCTTTAGGGTCCGGGCCCGGCCGGGCCATGGATTCAATGCCTGAGCCCGCGTCCCGC     60
F1:II.1       GGTCCTTTAGGGTCCGGGCCCGGCCGGGCCATGGATTCAATGCCTGAGCCCGCGTCCCGC     60
F2:II.5       GGTCCTTTAGGGTCCGGGCCCGGCCGGGCCATGGATTCAATGCCTGAGCCCGCGTCCCGC     60
F3:II.1       GGTCCTTTAGGGTCCGGGCCCGGCCGGGCCATGGATTCAATGCCTGAGCCCGCGTCCCGC     60
F4:II.4       GGTCCTTTAGGGTCCGGGCCCGGCCGGGCCATGGATTCAATGCCTGAGCCCGCGTCCCGC     60
F5:II.1       GGTCCTTTAGGGTCCGGGCCCGGCCGGGCCATGGATTCAATGCCTGAGCCCGCGTCCCGC     60
              ************************************************************

Reference     TGTCTTCTGCTTCTTCCCTTGCTGCTGCTGCTGCTGCTG---------------CTGCTG     105
F1:II.1       TGTCTTCTGCTTCTTCCCTTGCTGCTGCCGGCCC--------------------------     94
F2:II.5       TGTCTTCTGCTTCTTCCCTTGCTGCTGCTGCTGCTGCTGCTGCTGCTGCTGCTGCTGCTG     120
F3:II.1       TGTCTTCTGCTTCTTCCCTTGCTNNTGCTGCTGCTGCTGCTGCTGCTGCTGCTGCTGCTG     120
F4:II.4       TGTCTTCTGCTTCTTCCCTTGCTGCTGCTGCTGC--------------------------     94
F5:II.1       TGTCTTCTGCTTCTTCCCTTGCTGCTGCTGCTGCTGCTGCTGC---TGCTGCTGCTGCTG     117
              **********************  *** *   *

Reference     CCGGCCCCGGAGCTGGGCCCGAGCCAGGCCGGAGCTGAGGAGAACGACTGGGTTCGCCTG     165
F1:II.1       -------CGGAGCTGGGCCCGAGCCAGGCCGGAGCTGAGGAGAACGACTGGGTTCGCCTG     147
F2:II.5       CCGGCCCCGGAGCTGGGCCCGAGCCAGGSCGGAGCTGAGGAGAACGACTGGGTTCKCCTG     180
F3:II.1       CCGGCCCCGGAGCTGGGCCCGAGCCAGGCCGGAGCTGAGGAGAACGACTGGGTTCGCCTG     180
F4:II.4       -CGGCCCCGGAGCTGGGCCCGAGCCAGGCCGGAGCTGAGGAGAACGACTGGGTTCGCCTG     153
F5:II.1       CCGGCCCCGGCGCTGGGCCCGAGCCAGGCCGGAGCTGAGGAGAACGACTGGGTTCGCCTG     177
                     *** ***************** ************************* ****
```

Supplementary Figure S9. Verification of variants in the low-complexity region

Sanger validation of variants located within the low-complexity region (LCR) of CNPY3. All in-frame deletions and insertions identified within the LCR based on exome analyses were independently validated using Sanger sequencing. (**A**) Representative sanger sequencing traces showing the wild type control, homozygous in-frame deletions in individuals F1:II.1 (c.59_76del) and F4:II.4 (c.65_76del), as well as homozygous in-frame insertions in individuals F2:II.5 and F3:II.1 (c.62_76dup). Individual F5:II.1 shows a heterozygous in-frame insertion within the LCR (c.65_76dup) and is compound heterozygous for this variant and a missense variant identified by trio exome sequencing. (**B**) Multiple sequence alignment of exported sequences using Clustal Omega (reference: Madeira F, Madhusoodanan N, Lee J, et al. The EMBL-EBI Job Dispatcher sequence analysis tools framework in 2024. Nucleic Acids Res. 2024;52(W1):W521–W525).

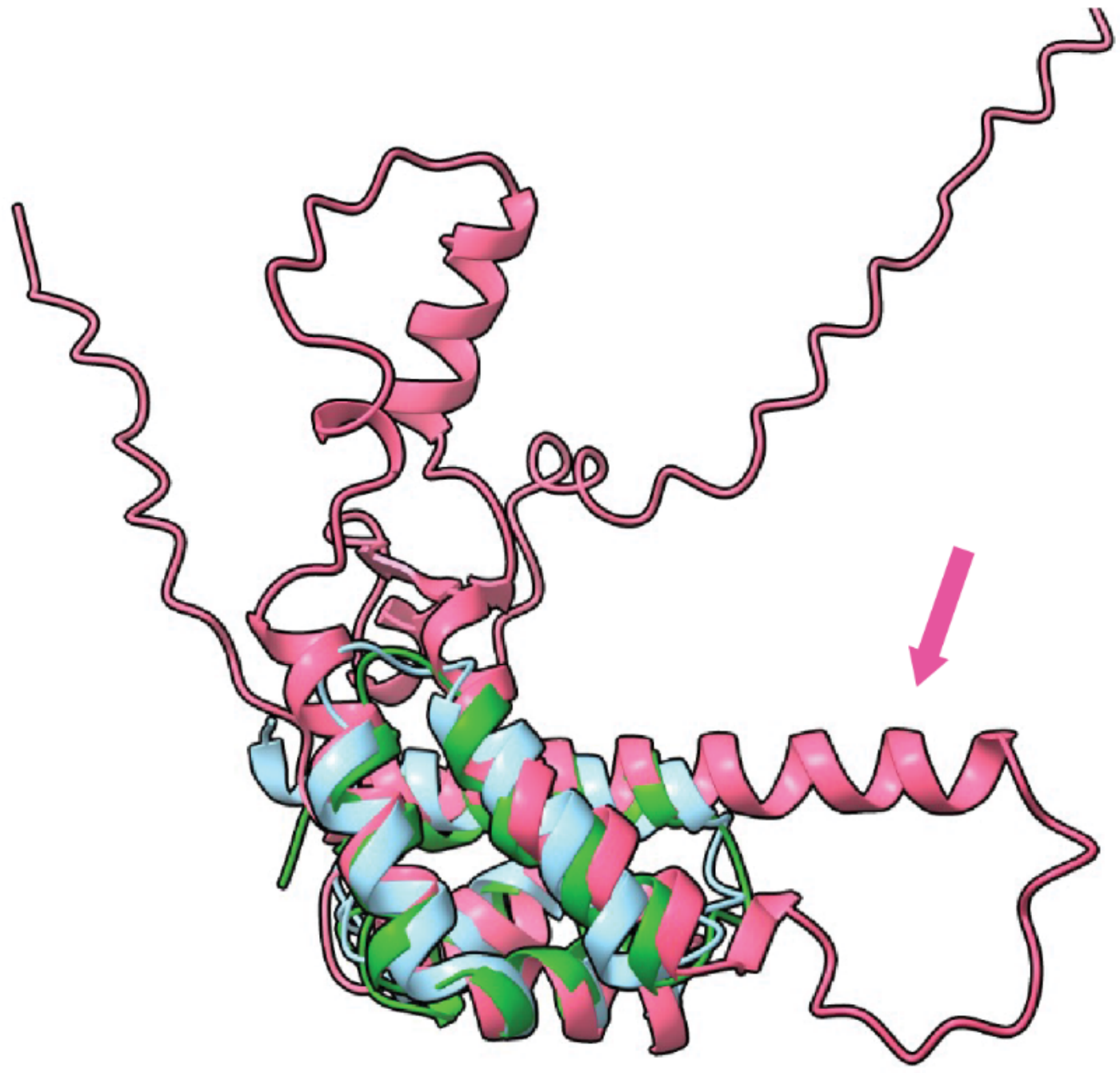

Supplementary Figure S10. CNPY3 structure predictions

Superposition of wild type CNPY3 (pink) with saposin family member NK-Lysin (pdb code: 1NKL; green) and saposin-like protein NA-SLP-1 (pdb code: 3S63; blue). The basic saposin fold is reflected in a similar orientation of the helices. However, the 5-residue loop between the saposin helical elements I and II in CNPY3 is extended by 23 additional residues, 13 of them extending helix I by approx. 3 turns (pink arrow). Similarly, the canonical 4-residue loop between helices II and III in the saposin fold is extended by 51 additional residues in CNPY3 allowing to accommodate the short anti-parallel β-sheet of 3 residues and a further 3-turn α-helix.

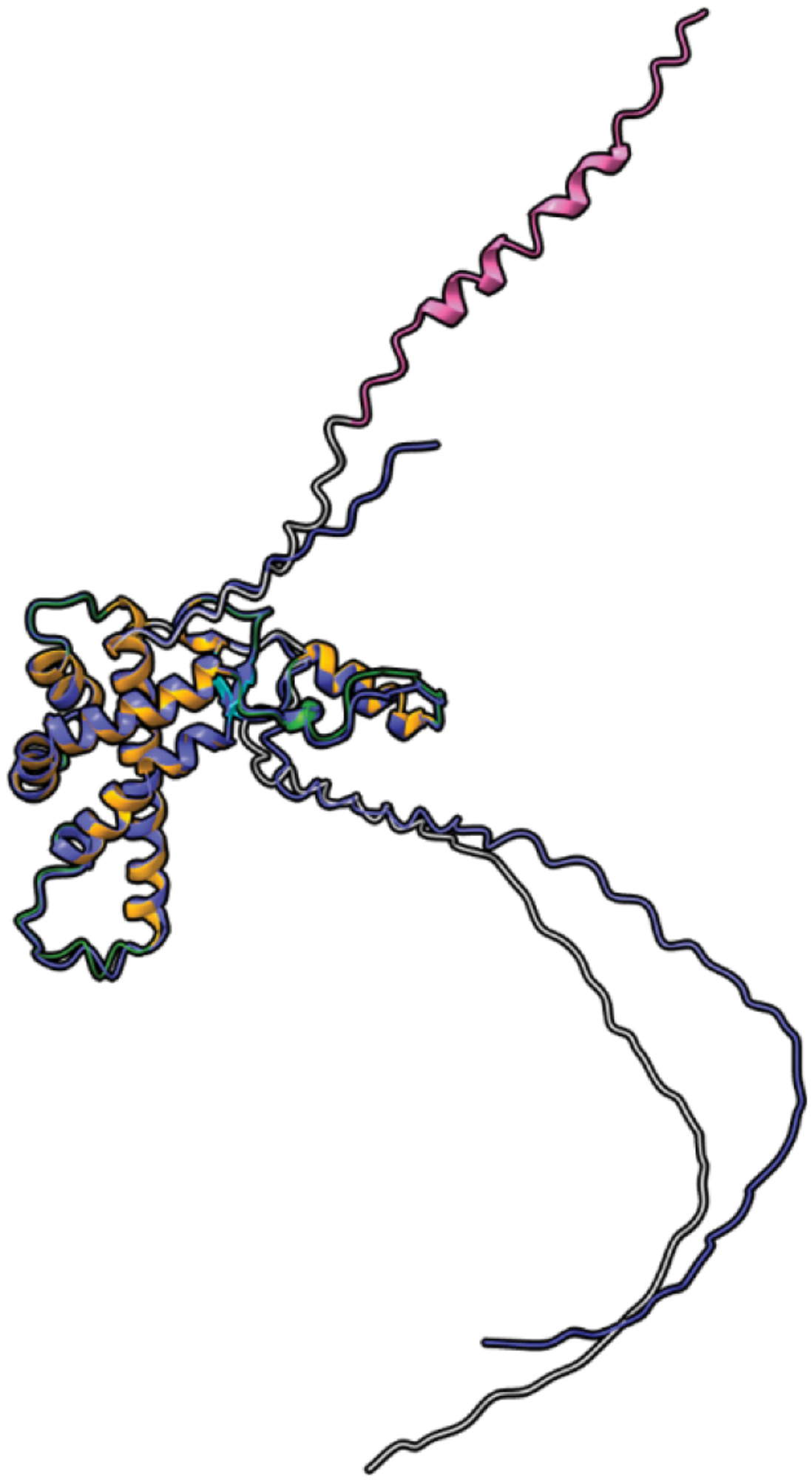

Supplementary Figure S11. AlphaFold models of full-length versus signal sequence-deleted CNPY3

Superposition of the AlphaFold predictions of full-length CNPY3 with CNPY3 lacking the signal sequence (blue). Cleavage of the signal sequence did not result in major differences in the tertiary structure of the molecular core as indicated by a root mean square deviation of 0.617 Å for the superimposition of backbone residues 48 to 208 (full-length numbering). Helices: orange, β-strands: cyan; loops and turns: green; N-terminus including a predicted α-helical stretch and signal sequence: violet.

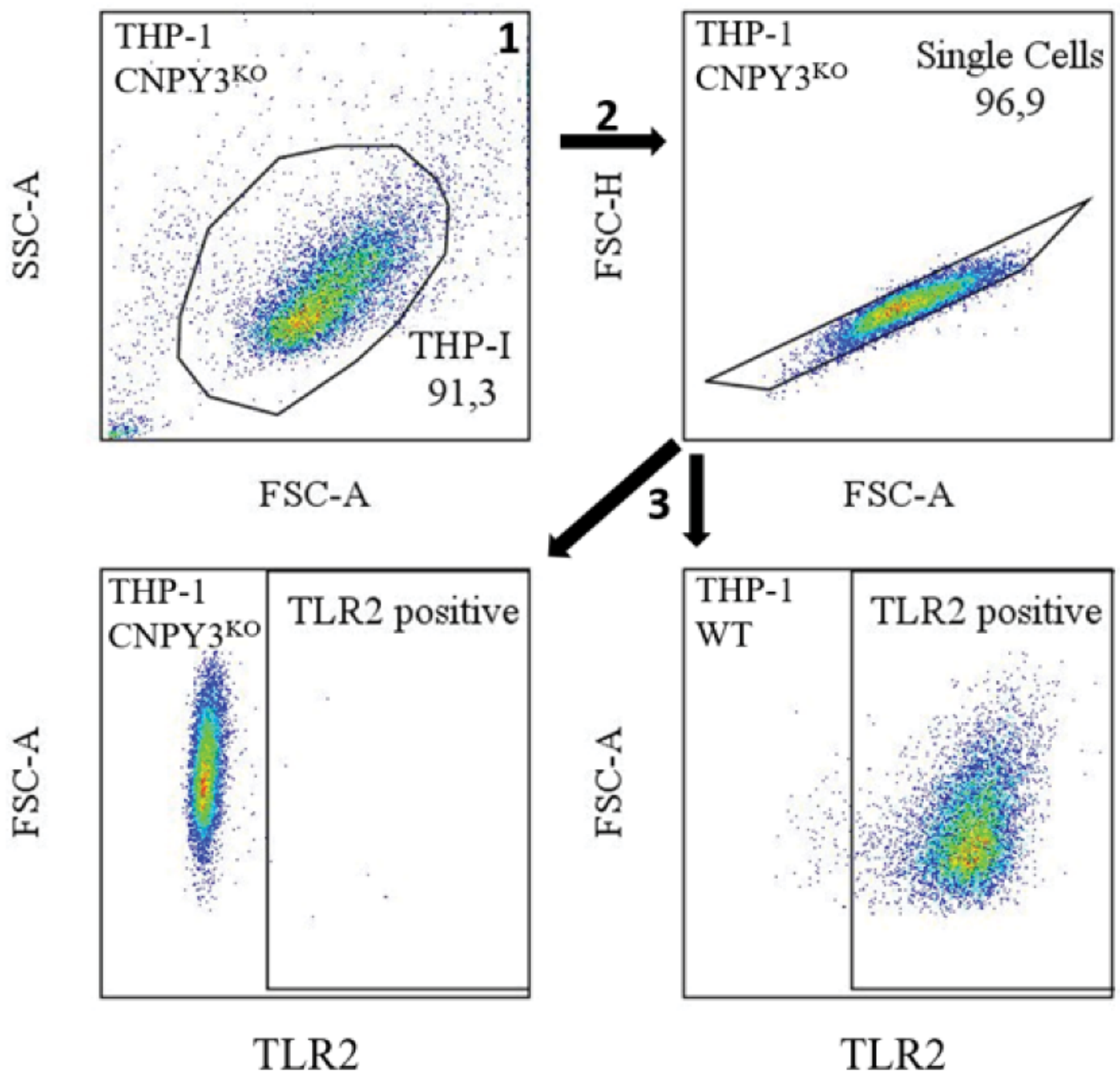

Supplementary Figure S12. Gating strategy for flow cytometric analysis of TLR2 surface expression in THP-1 cell lines

Cell debris (**1**) and doublets (**2**) were excluded from the analysis by appropriate gating. (**3**) The gate for TLR2 positivity was derived from the comparison of THP-1 wild type and THP-1 CNPY3^{KO} cell lines.

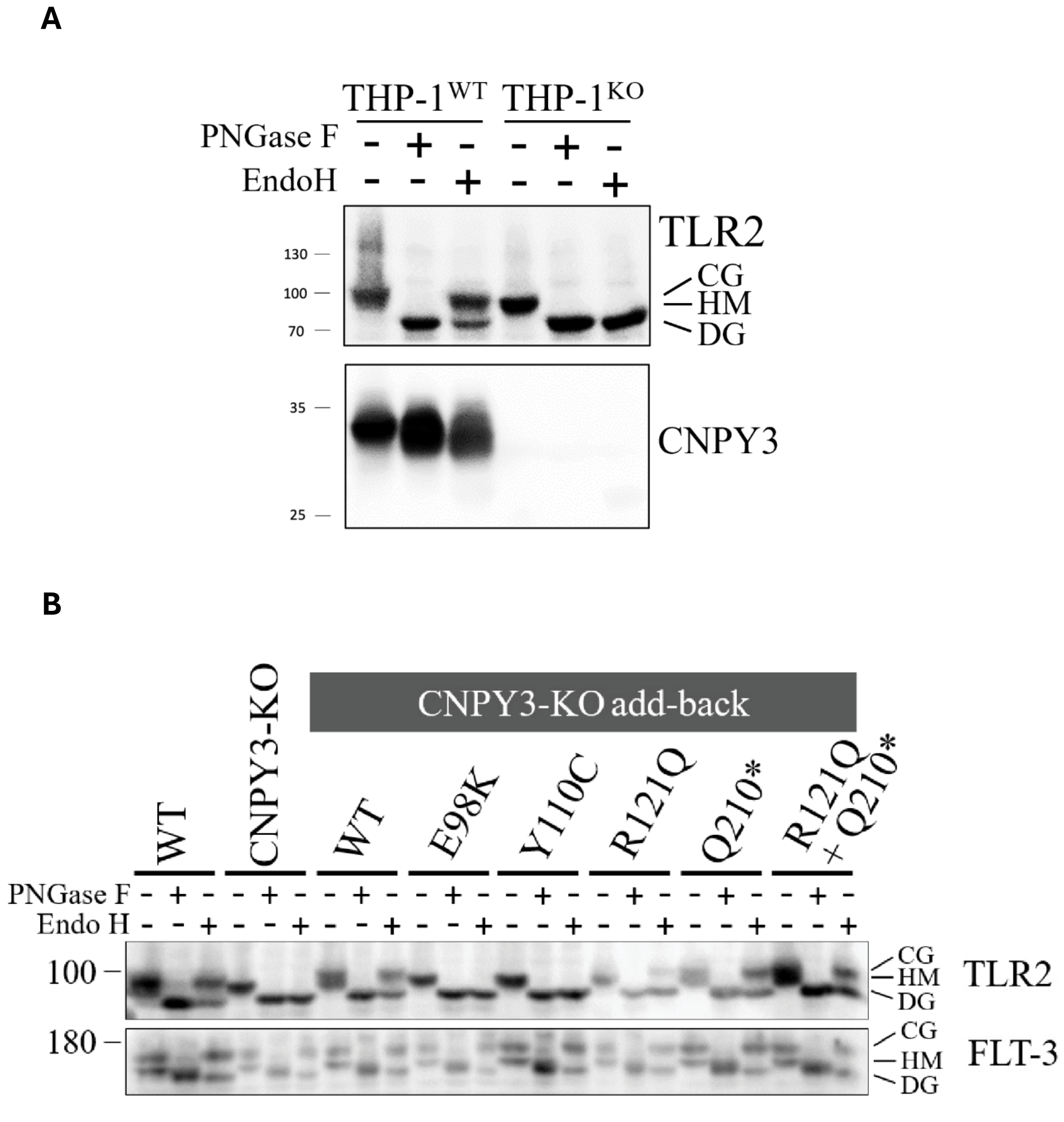

Supplementary Figure S13. Complex glycosylation of TLR2 in CNPY3 variant monocyte lines

(**A**) Cell extracts of the indicated THP-1 WT, CNPY3-KO cell lines were digested with PNGase F or Endo H as indicated and analysed by Western Blotting. Complex glycosylated (CG), high mannose (HM) and deglycosylated (DG) species are marked. Note the mobility shift in CNPY3, which is indicative of CNPY3 glycosylation. (**B**) Same experiment including add-back CNPY3 variant cell lines. Endo H does not cleave the complex glycosylated trees allowing to discriminate between HM and CG species. In R121Q, Q210* and R121Q+Q210* variant cells, Endo H is not able to fully digest the low mobility forms, undistinguishable from wild type cells, illustrating that TLR2 undergoes complex glycosylation in those variant cell lines. Glycosylation of the unrelated receptor tyrosine kinase FLT-3 is not affected by the CNPY3 genotype, confirming the specificity of CNPY3 action on TLR2.

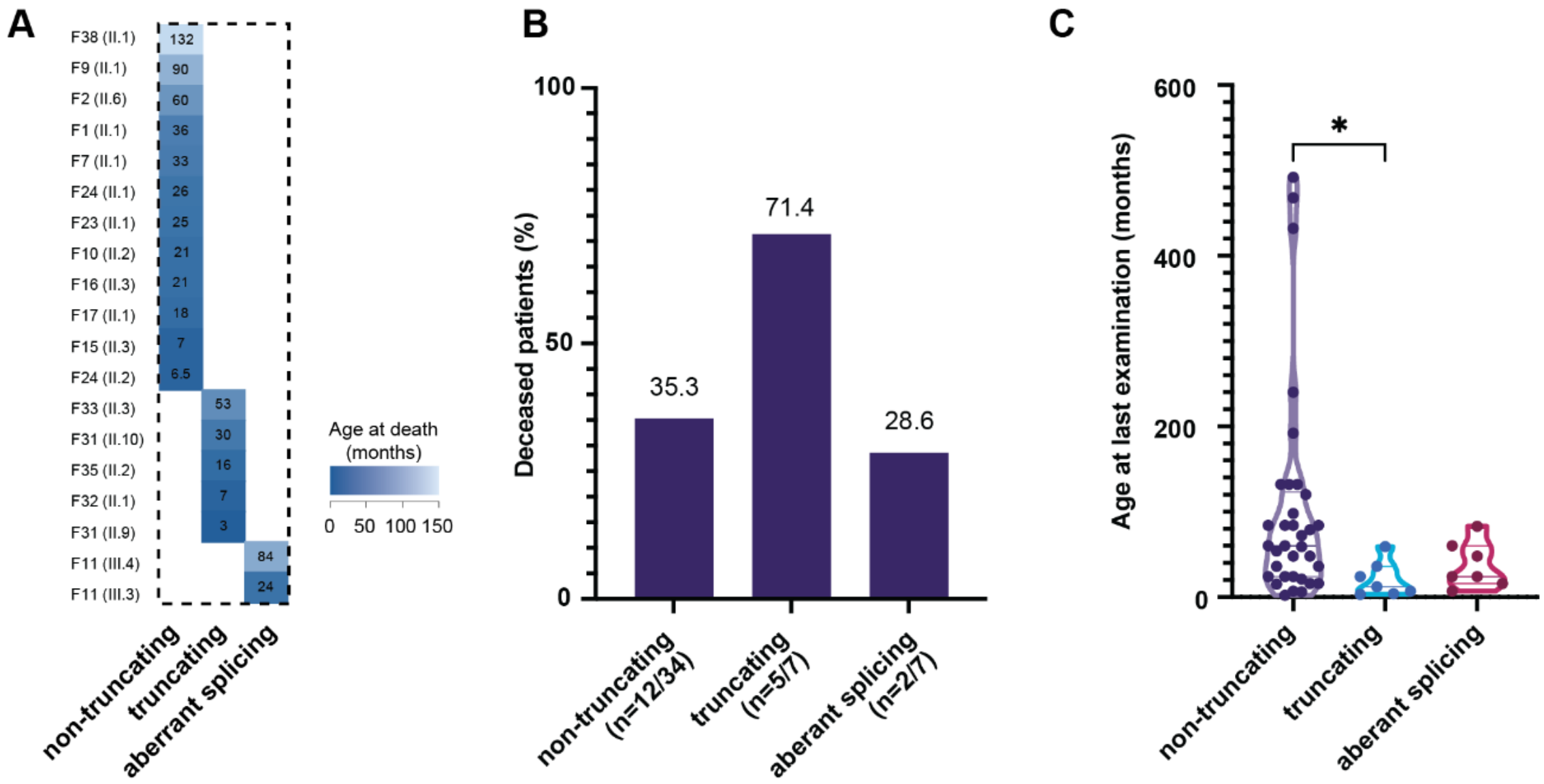

Supplementary Figure S14. Variant consequence and mortality

(**A**) Age at death of individual patients in relation to variant consequences. The age at death for patients with non-truncating variants ranged from 0.5–11 years (median 2.1; n=12), for those with truncating variants from 0.3–4.4 years (median 1.3; n=5), and for those with splice variants from 2.0–7.0 years (median 4.5; n=2). Given the limited numbers within each variant category, statistical analysis was not warranted, but a trend toward earlier mortality in patients with truncating variants may be suspected. (**B**) Proportion of deceased patients stratified by variant consequence. When patients were grouped according to variant consequence, the relative mortality (proportion of deceased among all carriers of the respective variant type) was higher among those with truncating variants. (**C**) Age at last examination stratified by variant consequences. Violin plots show the distribution of values; width reflects data density, the dashed line indicates the median, and the pointed lines represent the interquartile range. Group differences were analysed using a Kruskal–Wallis test followed by pairwise comparisons with Dunn's test. P-value: * < 0.05. Age at last examination, used as a surrogate for survival, was significantly higher in individuals with non-truncating variants, although follow-up times were heterogeneous. This group difference remained robust, retaining the same level of significance even after exclusion of the three oldest patients (data not shown), who exceed the age of the remaining cohort by a considerable margin.

Supplementary Case Reports

Family F1

Genotype: c.59_76del, p.(Leu20_Leu25del); homozygous

Family F1 consists of a 3-year-old girl (**F1:II.1**), the first child of healthy consanguineous parents of Azerbaijani descent (parents are first cousins). The pregnancy of the girl was full term and delivery was spontaneous vaginal delivery. She presented since 3 months of age with infantile spasms, which later developed to tonic and myoclonic jerks (without fever) at 6 months of age. This was associated with neurodevelopmental delay, including absence of speech and inability to sit, severe intellectual disability, severe loss of achieved milestones (such as neck holding) and failure to thrive. EEG revealed abnormal background with burst-suppression pattern - runs of high voltage of generalised burst of spikes and waves intermitted with period of suppression of background activity. She has been administered valproate and levetiracetam with partly effect. MRI carried out at 2 years of age showed diffuse brain atrophy. She also experienced major feeding problems, severe growth failure (her weight was 15 kg and height was 95cm) and recurrent (mostly respiratory) infections. Among the other neurological findings, she had microcephaly and spasticity. She died at 3 years of age because of long-term infection and pneumonia.

Second child of this family (**F1:II.2**) had a similar course of disease with a same onset age of seizures and seizure types, EEG features and developmental arrest. She also developed growth failure, spasticity and microcephaly. She died at 3.5 years of age because of pneumonia.

Family F2

Genotype: c.62_76dup, p.(Leu21_Leu25dup); homozygous

F2:II.5 is a male child born to healthy consanguineous parents of Iranian descent (first cousins). The pregnancy was full-term and uneventful, and delivery occurred via spontaneous vaginal delivery. Birth weight and head circumference were within normal limits. The neonatal period was unremarkable.

Seizure onset occurred at two months of age, initially manifesting as clusters of tonic spasms followed by myoclonic jerks, in the absence of fever. Seizure frequency progressively increased, becoming daily. Despite treatment with multiple antiseizure medications—including valproic acid, levetiracetam, clobazam, and phenobarbital—seizure control remained poor. EEG demonstrated a markedly abnormal background with a burst-suppression pattern that later evolved into multifocal epileptiform discharges, consistent with a diagnosis of early-onset epileptic encephalopathy.

Neurodevelopment was profoundly impaired. The patient never achieved head control or independent sitting, remained nonverbal, and exhibited severe global developmental delay. Neurological examination revealed microcephaly, chaotic eye movements, axial hypotonia with peripheral hypertonia, brisk deep tendon reflexes, and progressive spasticity. Feeding difficulties and frequent vomiting contributed to failure to thrive. Recurrent respiratory infections were also reported.

Brain MRI showed mild, diffuse cerebral and cerebellar atrophy including bilateral hippocampal atrophy with periventricular white matter signal changes, more prominent in the frontal regions. Extensive metabolic investigations—including tandem mass spectrometry (MS/MS) and gas chromatography–mass spectrometry (GC/MS)—were normal. Other laboratory studies and cerebrospinal fluid analysis were unremarkable.

The clinical course was progressive, characterised by severe epileptic encephalopathy, spastic quadriplegia, feeding impairment, and recurrent respiratory infections necessitating repeated hospitalisations.

F2:II.6 was the younger sibling of F2:II.5, born to the same parents. The pregnancy was full-term and uncomplicated, and delivery occurred via spontaneous vaginal delivery. According to maternal report, birth parameters were within normal limits.

Seizures began at two months of age, initially presenting as infantile spasms and myoclonic jerks, later evolving into generalised tonic seizures. As with her affected brother, seizures were refractory to multiple antiseizure medications. EEG recordings were unavailable for review at the time of reporting.

Severe neurodevelopmental impairment was evident from early infancy. She never achieved head control or sitting and remained nonverbal. Clinical documentation described microcephaly, chaotic eye movements, spasticity, and brisk tendon reflexes. Feeding difficulties and failure to thrive were marked. Ophthalmologic evaluation revealed poor visual tracking consistent with cortical visual impairment, while hearing was reportedly normal.

Neuroimaging data were not available; however, the clinical presentation suggested diffuse cerebral dysfunction. Comprehensive metabolic evaluations—including MS/MS, GC/MS, plasma amino acids, and urinary organic acids—were normal.

Her disease course closely mirrored that of her brother, marked by intractable seizures, profound developmental arrest, progressive spasticity, and recurrent infections. She died at five years of age due to pneumonia (COVID-19).

There are additional affected individuals from extended pedigrees of family F2 originating from the Ramhormoz region in southwestern Iran, all born to closely related parents and presenting with DEE (Supplementary Figure 5). The affected individuals who are homozygous for the familiar variant are as follows:

Male child **F2A:II.1**, who presented with severe visual impairment identified at 3 months of age, developmental delay, seizures with onset at 2 years, and a cerebral-palsy–like phenotype.

Male child **F2B:II.1**, who exhibited seizures beginning at 2 months, areflexia, neurodevelopmental delay, and severe neurological impairment.

Male child **F2C:II.1**, a 2.5-year-old boy with respiratory distress, developmental delay, hypotonia, speech delay, myoclonic jerks, and seizures.

Family F3

Genotype: c.62_76dup, p.(Leu21_Leu25dup); homozygous

F3:II.1 is a 7-year-old boy, born to consanguineous parents from Iran via emergency caesarean section at 35 weeks of gestation, was admitted to the neonatal intensive care unit immediately after birth because of respiratory distress. There were no complications during delivery, although rupture of the amniotic membrane had occurred prior to birth.

At seven months of age, developmental assessment revealed significant delays, including poor head control and generalised hypotonia. The child failed to achieve independent sitting and remained nonverbal throughout early childhood.

At the same age, he began experiencing episodes of myoclonic jerks accompanied by generalised seizures. These events have recurred approximately every one to two months and are currently managed with levetiracetam.

Although initial growth parameters were within normal limits, the past year has been marked by notable weight loss. The mother attributed this to decreased appetite secondary to seizure activity.

On current examination, the patient exhibits minimal responsiveness to environmental stimuli. Visual and auditory functions appear intact. There is no history of recurrent infections or evidence of immunodeficiency. Swallowing and gastrointestinal functions remain preserved.

Family F4

Genotype: c.65_76del, p.(Leu22_Leu25del); homozygous

Family 4 consists of an 8-year-old girl (**F4:II.4**), the fourth child of healthy consanguineous parents of Pakistani descent (parents are first cousins). Their first three children died at the ages of 2 years, 4.4 years, and 1.5 years, all were developmentally delayed, developed seizures (multiple types: clonic, tonic and myoclonic since early infantile life) with variable seizures severity (more severe in 3rd sibling with poor control). The pregnancy of the girl was full term. Birth length was 50 cm and head circumference was 34 cm. She achieved neck holding at 7 months, assisted sitting around 1 year after which developed regression in achieved milestones. She started to have tonic spasms since late infantile age, later GTC seizures and, neurodevelopmental delay, severe intellectual disability and failure to thrive. EEG examination was abnormal due to dysmaturation of background. She has been administered valproate and clobazam. Clinical examination showed spasticity, hearing and vision difficulties. Other clinical features included feeding difficulties, occasional vomiting and constipation. She has experienced recurrent respiratory infections, which led to immune compromise. An MRI showed non-specific brain atrophy.

Family F5

Genotype: c.65_76dup, p.(Leu22_Leu25dup); c.275+1G>A, p.(?); compound heterozygous

F5:II.1 is a 17-year-old female patient of Spanish descent, born to non-consanguineous parents. She had only one healthy sister, while a maternal uncle had an intellectual disability and marfanoid habitus. The pregnancy proceeded normally. The delivery was by caesarean section at 39 weeks. Her birth weight was 3860 g (+1.8 SD), and her height was 52 cm (+0.9 SD). The neonatal period was uneventful, but at four months, she began to present generalised hypertonic seizures, which persisted until three years of life. Subsequently, she has presented occasional episodes of disconnection with hypotonia and cyanosis. Several electroencephalograms performed in the first
six years showed no abnormalities, but those performed lately demonstrated bilateral frontal-central-parietal activity or acute waves in the right posterior region. She presented a severe global developmental delay since the first year. She always had autistic features, with little interest in communicating, a complete absence of verbal language (although she can express basic needs through gestures), irritability, stereotypes in the context of excitement, and disturbed sleep rhythm, but also aggressive behaviour, with frequent self-injuries and hyperactivity. She developed a profound intellectual disability. At the last follow-up at 17 years, she was under treatment with valproate, mirtazapine, clonazepam, aripiprazole, and levomepromazine, which managed to control her behavioural disorder. The physical exam at the first years of life showed hyperactivity, poor eye contact, lower limb spasticity with brisk deep tendon reflexes, and ataxic gait with flexed arms. At the age of 17 years, her weight was 82 kg (+2.5 SD), her height was 165 cm (+0.3 SD), and her head circumference was 53 cm (+0.5 SD). She had persistent drooling, with an open mouth, hypomimic face, and deep-set eyes. She had axial hypotonia with lower limb spasticity and absent deep tendon reflexes. Her movements were slow, and she had developed progressively a crouching gait. Magnetic resonance imaging showed a minimal increase in subarachnoid spaces at the temporal level and a decreased volume of the white matter in posterior regions, as well as an incomplete hippocampal inversion on the left. Extensive metabolic testing showed no relevant abnormalities, and genetic studies encompassing karyotype, array-CGH, MECP2, and Angelman studies were negative. The peripheral neurophysiological study and visual evoked potentials were also normal.

Family F6

Genotype: c.172G>A, p.(Glu58Lys); homozygous

The patient **F6:II.1** is an 8-year-old male, first child to healthy consanguineous parents of Pakistani origin. He was born at full term without complication during vaginal delivery. The family first concern was at age of 6-8 months, when the parents observed developmental delay and later, he had epileptic seizures. The affected individual suffers from global developmental delay (difficulty in holding head, crawling, difficulty in holding things, cannot walk, wheelchair bound), hypotonia, infantile spasms, intractable seizures, and absence of speech.

Brain-MRI showed fused sagittal suture, the rest of the sutures are open. There is flattening of the right occipital bone related to postural remodelling. There is no intracranial structural abnormality and the brain shows normal grey-white matter differentiation. The ventricles appear unremarkable and the posterior fossa and midline structures are maintained. The cervical spine appears unremarkable. Paravertebral soft tissues and airways appear unremarkable.

Skull X-rays suggesting an early fusion of the right coronal suture with prominent right orbital bone outline. Mild degree of generalised widening of cerebral sulci with prominent lateral and third ventricles and prominent sylvian cisterns and interhemispheric fissure are observed. Normal unmyelinated supratentorial white matter and no focal lesion or mass is observed. Normal myelination, fourth ventricle, cerebellum and brainstem appear normal. Basal cisterns are prominent with no midline shift and no obvious evidence of infarction or haemorrhage is observed. Thus, suggesting sagittal craniosynostosis.

EEG revealed burst suppression pattern during sleep, showing multifocal epileptiform discharges and diffuse slowing. ECG and other skeletal and laboratory examination were unremarkable.

Family F7

Genotype: c.275C>T, p.(Ser92Leu); homozygous

Family 7 consists of a deceased 2.9-year-old girl **F7:II.1** and a 2-year-old boy **F7:II.2** from a consanguineous family of Pakistani descent (parents are first cousins). The pregnancy of the girl was complicated by a premature leak and breech and delivery was via lower segment caesarean section. The girl presented at the age of 3 months with tonic seizures with up rolling of eyes, and exaggerated startle response around 6 months of age.

The boy was diagnosed at 1 week old with neonatal irritability and bilateral club foot disease. He started experiencing GTC and myoclonic jerks (1-2 episodes per day with increase to multiple times with intercurrent illness). EEG examination in the boy was abnormal due to dysmaturation of background. The girl experienced status epilepticus twice.
The girl was administered phenobarbital, levetiracetam, nitrazepam and valproic acid while the boy had been given levetiracetam and nitrazepam. The seizures were poorly controlled in both the siblings, 1-2 episodes daily. Both had global developmental delay, with no neck holding, poor gaze fixation, as well as failure to sit, walk or thrive and severe intellectual disability. In the oldest sib, there was evidence of motor, speech, vision, hearing and cognition difficulties. Ophthalmological examination of the 4-year-old girl showed decreased vision with refractory error of 6 with disc pallor. No hearing assessment done in either proband. Growth parameters for the boy at 2 years of age was 45.5 cm head circumference (-2.2SD), height of 72 cm (-4.3SD) and weight of 6 kg (-5.5SD). Other clinical features included feeding difficulties, occasional vomiting, constipation and scoliosis. They both experienced recurrent respiratory infections, which led to immune compromise and early death of the girl at the age of 2.9 years.

Family F8

Genotype: c.275C>T, p.(Ser92Leu); homozygous

F8:II.3 is a three-year-old boy of Pakistani origin born to consanguineous parents. His antenatal course was unremarkable, and he was initially stable at birth. He subsequently developed respiratory distress and required admission to the neonatal intensive care unit for four days. Seizures began on day two of life, remitted briefly, and recurred on day 40 as generalised tonic–clonic events lasting about one minute. The seizures have remained refractory despite phenobarbital and levetiracetam; sodium valproate was added after 12 months of age. EEG showed a burst suppression pattern and CCT scan at 2 months of age with increased sulcal and fissural depth and width and increased size of bilateral temporal horns was suggestive of global cerebral atrophy.

He has global developmental delay with regression. He achieved head control at approximately 12 months, and an early social smile was later lost. He has laryngomalacia with persistent stridor, and breathing issues which worsen during crying. The last documented examination at six months recorded a weight of 5 kg and a head circumference of 40 cm.

A sibling died at two and a half years of age with a similar condition, including seizure onset at two months.

Family F9

Genotype: c.275C>T, p.(Ser92Leu); homozygous

The male child **F9:II.1** baby was born to an Indian muslim couple in a first-degree consanguineous relationship. The birth at term was uneventful.

Developmentally, he showed global delay, which the parents first noted at 2 months of age. By 3 months, he began having tonic seizures, which later evolved into tonic spasms. At its best, seizure frequency was 4–5 episodes per day. EEG revealed a burst suppression pattern.

An MRI performed at 8 months of age revealed a bulky and upwardly tented corpus callosum.

The child exhibited significant failure to thrive, truncal hypotonia with appendicular spasticity, cortical visual impairment, and no developmental progress. He had no obvious dysmorphic features, except for slightly coarse facial features, an umbilical hernia, and mild hepatosplenomegaly. The eye examination was normal.

He received six different anti-seizure medications, none of which were effective. Eventually, the family opted to discontinue them. The child passed away at 7.5 years of age due to aspiration pneumonia.

Family F10

Genotype: c.275C>T, p.(Ser92Leu); homozygous

The male child **F10:II.2**, second child of a third-degree consanguineous marriage, with uneventful antenatal history, was born full term by caesarean section with a birth weight of 2.5 kg, without perinatal or neonatal complications.

Seizures began at 3 months of age, presenting as tonic posturing of all four limbs and drooling, lasting 30 minutes, followed by post-ictal drowsiness. A similar event occurred the next day. The child was hospitalised for 13 days. The child continued to have around 2-5 seizures daily during the initial 8 days, with some reduction in the duration. The child was started on Levetiracetam with minimal response. Further, phenobarbital was added with subsequent seizure control.
EEG showed multifocal epileptiform discharges with secondary generalisation and suppression. MRI brain and fundus exams were normal.
General examination: weight 5.7 kg, head circumference 37 cm. Dysmorphism noted included coarse facial features, low-set ears, corneal opacity, and umbilical hernia. The neurological exam showed spasticity with brisk deep tendon reflexes and preserved muscle strength. Abdominal examination and skeletal survey were normal. Mucopolysaccharidosis and other lysosomal storage disorders workup was negative.
At 6 months, epileptic spasms emerged with some response to prednisolone; then vigabatrin, nitrazepam and perampanel were added. Spasms continued at reduced frequency but never ceased; lamotrigine and clobazam were further added, with some response, but no complete seizure cessation.

At 1 year 3 months, the child had fever, pneumonia and collapse of the upper lobe of the lung, treated with antibiotics and respiratory support. He presented generalised tonic-clonic seizures, and encephalopathy. EEG showed generalised spike and sharp waves at irregular intervals with diffuse slowing. Zonisamide and valproic acid were added; tonic seizures ceased, but spasms continued.

The child was again hospitalised at 1 year 7 months, with pneumonia and right upper lobe collapse, sepsis and encephalopathy, treated with symptomatic therapy and antibiotics. Nasogastric feeds were started. During this admission, the child developed right focal seizures, phenytoin was added and dose escalated to maximum as per body weight with some response. The child could not be weaned off respiratory support, was discharged against medical advice at parental request, on home oxygen.

Developmentally, the child never attained neck control, social smile, or recognition of parents. He died at 21 months due to status epilepticus and respiratory compromise.

Family 11

Genotype: c.275+1G>A, p.(?); homozygous

Male patient **F11:III.3** was born to distantly related Afghani parents at term and had severe hydronephrosis detected antenatally due to pelvi-ureteric junction obstruction. Seizures occurred from 1st month of life. At 10 months his development was severely delayed. He was fixing but not following with his eyes and he had a social smile. His hearing was normal. He had gastroesophageal reflux and was fully nasogastrically fed.

He had an intractable seizure disorder with focal seizures and infantile spasms. He had an acquired microcephaly, his head circumference was 40.5 cm at 10 months (which is below the 2nd centile). He had severe motor impairment with truncal hypotonia and increased tone peripherally. He appeared facially similar to the younger brother F11:III.4. He had a glabella haemangioma, bitemporal narrowing, a tented mouth and upturned fleshy ear lobes. He had a transverse palmar crease on the left. An MRI at 5 days of age showed a posterior fossa subdural collection consistent with birth trauma and patchy petechial haemorrhages in the deep white matter. The white matter was abnormal in appearance, with periventricular cystic changes, and ventricular widening. Additional findings include partial agenesis of the septum, partial cavum septi pellucidi, and an abnormal configuration of both hippocampi. Repeat MRI at 21 months showed increased ventricular dilatation and excess CSF spaces around the brain consistent with progressive cerebral atrophy. The periventricular cystic change was no longer visible. He died at two years of age.

The younger brother **F11:III.4** was born by ventouse assisted delivery following an uneventful pregnancy. There was meconium at delivery, and the cord was around his neck. He was floppy and pale and required stimulation at birth. His Apgar scores were 4/8/9. He had respiratory distress and was treated with antibiotics and CPAP for 3 days. He had stridor which improved. He was commenced on trimethoprim because of antenatal renal pelvic dilatation, however postnatal scans were normal. Immediately after birth he was noted to be floppy with focal seizures from the newborn period. He developed intractable epilepsy with focal seizures and infantile spasms. His development was profoundly globally delayed. He was not able to fix and follow. His hearing was normal. He had an acquired microcephaly. On examination he had severe microcephaly, truncal hypotonia and increased peripheral tone with dystonic spasticity. Facially he had bitemporal narrowing of the upper face and full cheeks. He had large eyes with long eye lashes and low set ears. His fingers were tapering with small nails. Due to an unsafe swallow and gastro-oesophageal reflux, he had a fundoplication and gastrostomy. He had recurrent chest infections requiring admission to intensive care and passed away at 6 years 11 months of age. An MRI brain scan showed periventricular white matter changes and cyst formation.

A CT scan at age 5 and 7 showed multiple small intracranial calcific foci and progressive generalised cerebral atrophy. He has had extensive normal metabolic investigations, normal microarray studies and normal testing on the epilepsy gene panel.

There are two older sisters that are healthy and developmentally normal. Both parents are healthy. They have had one early miscarriage.

Family 12

Genotype: c.284G>A, p.(Arg95Gln); homozygous

The Iranian family consists of four offspring, including three similarly affected individuals born to healthy parents who are non-consanguineous, yet originated from the same region/village.

All three patients were born at term after an unremarkable pregnancy and neonatal period. Birth weight, length and head circumference are not available. The female case **F12.II.1** and her two affected male siblings (**F12.II.2**, **F12.II.3**) aged 41, 39, and 36 years, respectively at the time of examination. They presented with psychomotor delay starting at the age of two years, including poor head control, delayed ability to walk and impaired speech development. All manifested moderate intellectual disability, aggressive behaviour, and a history of generalised tonic-clonic seizure (being under medication). No spasticity and dysmorphic features were noted.

At the age of 41 years, the weight of F12.II.1 was 83 kg, the height was 153 cm and the head circumference measured 55 cm.

Seizures started at two years of age and the following EEG revealed slow waves with some fasciculation. In F12.II.2 the onset of seizure was at the age of two years, followed by a second episode at 27 years. Height and head circumference were 174 cm and 54.5 cm, respectively at the age of 39 years. F12.II.3 manifested seizures at two months of age with the second episode occurred at 25 years. The height was 177 cm at 36 years of age and head circumference 54 cm.

Family 13

Genotype: c.292G>A, p.(Glu98Lys); homozygous

F13:II.1 is a 5 years old girl born term to unrelated German parents, a 2.5-year-old sister is healthy. Birth measurements were within normal ranges. Seizures occurred at three months of age.

Serious epileptic encephalopathy is present. She continues to have several small seizures daily. Major seizures are treated with emergency medication (midazolam or diazepam). Smaller series, which she has daily, are not treated. As permanent medication, she is given levetiracetam, valproic acid and gabapentin, as gastric protection omeprazole, and dronabinol to treat spasticity.

The girl does not show any speech. She occasionally follows toys with her eyes and sometimes looks at the mother in a concentrated manner. However, she knows the parents from her voice and allows the parents to calm her down. She had a gastrostomy tube placed at 2.5 years of age, and in the meantime a percutaneous intestinal tube, because she has aspirated more often with the gastric tube. A normal meal is not possible because of the high risk of aspiration.

The motor development is clearly retarded. She often holds her hand in a fisted position. Sitting, standing, walking, crawling, and turning are not possible. However, the girl can relax in the water.

The head circumference of the girl 5 years of age was 44.5 cm (-5.7 SDS).

The occiput appeared clearly flattened. Facial features included laterally thinned eyebrows despite overall broad eyebrows, which the healthy sister also has. The nose was small, with a short philtrum and slightly anteverted nares. She had a high-arched palate, and the ears were slightly posteriorly rotated and low-set. No other significant dysmorphic features were noted.

A brain MRI study done in 5th year of life at was inconspicuous.

Family 14

Genotype: c.292G>A, p.(Glu98Lys); homozygous

Individual **F14:II.1** is an 11 years old male with intractable epilepsy, microcephaly, intellectual disability, optic atrophy, spastic paraplegia, and short stature. At this time his seizures are generally well controlled and may occur 3-6x/day when ill and each last about 40 seconds. For seizure management he is on oxcarbazepine, levetiracetam, and rufinamide.

The pregnancy and birth process were uncomplicated, and he was initially feeding well. About two weeks after receiving vaccinations at the age of two month, he had his first seizure. These seizures were initially extremely recurrent and led to a long admission. He used to have them every hour and after initiation of multiple regimen anticonvulsants, the frequency came down.

He essentially never gained any speech. He was never able to sit by himself. His head control has slightly improved over time.

He has significant hypotonia and is unable to make any usage of his hands. He does not have any social interactions and does not follow any faces. He is nonverbal. He has cortical blindness. His hearing is normal. He is G-tube fed.

The family history is notable for parents having 3 spontaneous abortions and 1 stillbirth at 4 months gestational age. The parents are niece and uncle, respectively. Mother's maternal half-sister has epilepsy which started at 8 months. One of the patient's father's sisters has seizures at 21 years old and some additional mental health concerns. Ancestry is Hispanic.

Family 15

Genotype: c.329A>G, p.(Tyr110Cys); homozygous

F15:II.3 is a deceased 7-month-old boy, the third child of healthy consanguineous parents of Pakistani descent (parents are first cousins). The pregnancy was full term with experienced anaemia and oligohydramnios in the mother. Delivery was via C-section. The boy presented with brief apnoeic episodes with cyanosis on day 1 postnatally and an echo examination performed at day 2 showed ASD with moderate pulmonary hypertension. He later started presenting on day 18 with myoclonic seizures, and then changed to tonic in the 5th month of life. He was diagnosed with encephalopathy even though an EEG showed normal background with no discharges. He has been administered phenobarbital and levetiracetam. Upon neurological examination, he is spastic and shows failure to thrive. He never achieved neck holding. He was found to have both impaired vision (no gaze fixation or following, no social smile) and hearing (poor response to voice). According to his parents, he also experienced feeding problems. His ammonia and lactate levels were raised. He passed away at 7 months of age due to probable aspiration secondary to vomiting.

His elder brother F15:II.1 also passed away at 9 months of age due to a similar condition.

Family 16

Genotype: c.329A>G, p.(Tyr110Cys); homozygous

F16:II.3 is a 1-year and 5-month-old male, born to consanguineous parents from Pakistan. He had two older siblings, aged 6.5 and 5.5 years, both developmentally normal. There was no family history of seizures or developmental delay. He was born preterm at 32 weeks via emergency lower segment caesarean section, with a birth weight of 1.5 kg. Postnatally, he developed neonatal jaundice requiring phototherapy for three days. There were no episodes of hypoglycaemia or neonatal seizures. He had respiratory distress syndrome and was admitted to the neonatal ICU for seven days, where he received oxygen therapy, CPAP, and IV antibiotics, and was discharged in stable condition.

He was first evaluated by neurology at the age of 4 months and 20 days, when he was hospitalised with cough and respiratory distress, and had frequent arching episodes. At that time, his weight was 2.5 kg, he had excessive oral secretions, a provisional diagnosis of Sandifer syndrome with bronchopneumonia was made. However, with persistent posturing, an EEG showed frequent bilateral posterior quadrant (occipital dominant) spikes, polyspikes, and sharp and slow waves. Levetiracetam was initiated. His respiratory symptoms improved, and he was discharged.

He was hospitalised six times due to respiratory distress and posturing episodes. Over subsequent clinic visits until 1 year and 5 months of age, he continued to have seizures (generalised body stiffness episodes), failure to thrive and developmental delay. At his last follow-up at 1 year and 8 months, had global developmental delay, no visual fixation and tracking.

He had marked microcephaly, long eyelashes and thick eyebrows. His length, weight, and head circumference were all below the 2nd percentile for age. Neurological examination showed global developmental delay, partial neck control, however, no other motor milestone was achieved. He also had globally reduced muscle bulk, spasticity in all four limbs, and brisk deep tendon reflexes. The rest of the systemic examination was unremarkable. Due to persistent posturing, phenobarbital was added. He had ongoing issues of posturing, as well as poor sucking, frequent oral secretions, and failure to gain weight, prompting the decision to place a gastrostomy tube. The child passed away at 1 year and 9 months of age due to severe respiratory distress.

Family 17

Genotype: c.329A>G, p.(Tyr110Cys); homozygous

The male child **F17:II.1** was born to Indian Muslim parents with no known consanguinity. He was delivered prematurely at 30 weeks of gestation, with a birth weight of 1.2 kg, which was appropriate for gestational age. The neonatal period was complicated by respiratory distress, transient hypothyroidism, and neonatal jaundice.

At the age of 5 months, he developed tonic seizures, followed shortly by erratic myoclonic movements, both of which persisted despite altogether seven anti-seizure medications. EEG revealed a burst suppression pattern, while the brain MRI was unremarkable.

The clinical course was marked by profound global developmental delay, with no acquisition of developmental milestones, absent speech, encephalopathy, apnoeas, and microcephaly. Neurological examination revealed generalised central hypotonia with intermittent dystonic posturing and absent visual tracking. Dysmorphic features included a round face, small nares, and a long philtrum. He also exhibited failure to thrive and hepatosplenomegaly. Despite nutritional concerns, the family declined gastrostomy tube placement.

The child passed away at 18 months of age due to aspiration pneumonia.

Family 18

Genotype: c.337C>T, p.(His113Tyr); homozygous

F18:II.3 is a 12½-year-old male child born to Indian parents in a third-degree consanguineous marriage. He was delivered vaginally at term with a birth weight of 3.6 kg, and there were no perinatal complications.

At 3 months of age, he developed multifocal clonic seizures requiring multiple antiseizure medications, including levetiracetam, valproic acid, and lacosamide. Around 7 months of age, he developed myoclonic jerks. EEG was suggestive of multifocal discharges. MRI brain was normal. He had mild motor delays and was walking independently at 15 months. However, he exhibited frequent falls and moderate spasticity (grade 2 on the MAS) in both lower limbs. Significant speech delay was noted; by 7 years of age, he was only able to speak a few meaningful words. He also developed behavioural issues that required medications. The child has been seizure-free for the past year under treatment with valproic acid and lacosamide. He remains partially dependent for activities of daily living and is attending school, although with academic concerns.

Since the age of 12, the patient's walking difficulties have progressively worsened, accompanied by the development of parkinsonian features, including generalised rigidity, festinant gait, and impaired postural reflexes. Treatment with levodopa/carbidopa and trihexyphenidyl was initiated, resulting in a partial clinical response, while the patient is also receiving regular physiotherapy. This has been slowly worsening over past one year and the patient has stopped participating in games in past
6 months and stopped going to school.

A male sibling was similarly affected and died at 6 weeks of age.

Family 19

Genotype: c.362G>A, p.(Arg121Gln); homozygous

F19:II.1 is an 11-year-old boy born at term from the first pregnancy and first delivery to consanguineous parents from Kazakhstan. The pregnancy was complicated by iron-deficiency anaemia (stage 1–2) and oligohydramnios. Birth weight was 3498 grams, length 51 cm, head circumference 34 cm. Apgar scores were 7 and 8 at 1 and 5 minutes, respectively. Neonatal period was unremarkable except for hypotonia, and the child was discharged from the maternity ward on day four.

Since birth, the child experienced paroxysmal episodes characterised by generalised muscle stiffness, leg abduction, backward head extension, jaw clenching, and tonic upward/rightward gaze deviation. These events occurred unpredictably, with each episode lasting approximately 30 seconds and sometimes repeating 2-4 times within five minutes. He gained head control from 1.5 months, sat up at 6 months, crawled at 8 months, but started walking independently only from the age of 5 years. At age 2 years and 1 month, he was found to be at the fourth (pre-verbal) level of speech development. At age 2 years and 3 months, he was diagnosed with mixed specific developmental disorder with autistic features.

Ophthalmologic evaluation at 9 months of age showed a normal fundus. Brain MRI at 10 months revealed mild enlargement of subarachnoid CSF spaces. EEG at that time showed normal sleep architecture without epileptiform activity.

Abdominal ultrasound and ECG were unremarkable except for mild sinus tachycardia and, later, partial right bundle branch block. Repeat brain MRI at age 4 years revealed multiple white matter lesions and bilateral frontal gliosis in close contact with the cortex, along with moderate ventriculomegaly.

Serial EEGs demonstrated progressive changes with emerging bilateral disorganised polymorphic slow-wave activity, and later, distinct epileptiform discharges, most prominently over central, parietal, and posterior temporal regions. By age 7, epileptiform activity included sharp waves, spike-wave, and polyspike-wave discharges, more prominent on the right. Epileptiform foci expanded to involve occipito-parietal and posterior temporal regions bilaterally. A diagnosis of focal frontal epilepsy with secondary generalisation and startle-induced seizures was made by a paediatric epileptologist.

His dysmorphic features included: slender build, dolichocephaly, prominent forehead, frontal bossing, long face, short philtrum, broad nasal tip, anteverted nares, thick lips, everted upper lip vermilion.

F19:II.3 is a 4-year-old girl born at 36 weeks of gestation via caesarean section from the third pregnancy. The pregnancy was complicated by iron-deficiency anaemia and vaginal candidiasis.

Birth weight was 2845 grams, length 51 cm, head circumference 35 cm. Apgar scores were 7 and 8 at 1 and 5 minutes, respectively. The neonatal period was marked by respiratory distress, suspected infection, and transient tachypnoea of the newborn, requiring admission to the neonatal intensive care unit. She was discharged from the hospital on day 19 of life.

Clinical seizures were first noted at the age of 3 months, described as clonic jerks involving primarily the lower limbs, with episodes of sustained gaze fixation and ocular proptosis. In terms of developmental milestones, she gained head control from 1.5 months, sat at 6 months, crawled at 8 months, but failed to gain independent walking yet.

Serial EEG studies provided evidence of early epileptiform abnormalities including video EEG monitoring at age 1 showing preserved and organised background activity of infantile type. During sleep, bilateral asynchronous epileptiform discharges were recorded over the central regions. A clonic seizure of 30 seconds duration was captured, beginning with rhythmic sharp wave activity over the central regions, more pronounced on the left, evolving into spike-slow wave complexes. Repeated EEG a month later showed sleep architecture as consistent with the infantile pattern; sleep spindles were present with adequate index. Isolated sharp waves and spike-wave complexes were seen in central and frontal leads, bilaterally and asynchronously. The overall index of epileptiform activity was low. During recorded myoclonic jerks and limb twitches (observed in both wakefulness and sleep), no concurrent epileptiform discharges were detected, although diffuse high-amplitude delta activity was seen during these events.

Ophthalmologic evaluation on at this age revealed retinal vascular angiopathy. Echocardiography identified a patent foramen ovale (PFO), probable atrial septal defect (ASD), and a restrictive patent ductus arteriosus (PDA). Cranial neurosonography demonstrated hypoxic changes and immature brain structures. MRI of the brain at age 1 did not reveal any structural abnormalities.

Her dysmorphic features included: dolichocephaly, thick hair, prominent forehead, frontal bossing, short philtrum, broad nasal tip, depressed nasal bridge, and widely spaced nipples.

Family 20

Genotype: c.362G>A, p.(Arg121Gln); c.628C>T, p.(Gln210Ter); compound heterozygous

Individual **F20:II.2** is the second child from the fourth pregnancy of healthy, unrelated parents of Ukrainian–Armenian origin. The first pregnancy resulted in a healthy boy; the second and third were terminated at maternal request due to severe toxicosis. The fourth pregnancy was complicated by maternal toxicosis, dyspnoea, oedema, and an acute respiratory viral infection. Delivery occurred by planned caesarean section at 41 weeks due to a prior caesarean section. Birth weight was 3160 g, length 50 cm, and Apgar scores were 8/8. Perinatal hypoxia was reported. The patient established visual contact by 2 months, held her head at 3 months and sat independently by 1 year, indicating delayed motor development, while psycho-speech development was initially age-appropriate.

Epileptic manifestations began at 2.5 months, initially with tremor-like movements of the legs, later involving the hands, occurring mainly in association with feeding. Pantogam (hopantenic acid) was administered and tremors resolved by 5.5 months. Between 6 and 12 months, she exhibited brief series of extensor head movements and generalised tonic posturing, also often triggered by eating, and occasional left leg clonic activity. At 6 months, EEG showed no epileptiform activity, and these early episodes subsided spontaneously.

At 7 months, Cortexin (polypeptide-based nootropic substance) and physiotherapy were prescribed without clear benefit. At 11 months, seizures were provoked by bright light and characterised by loss of consciousness, eye deviation, head tilting, and neck stiffening, lasting less than a minute. A similar event at 1 year led to hospitalisation; EEG revealed epileptiform discharges, while MRI was normal. Valproic acid therapy was initiated but initially associated with an increase in seizure frequency. Addition of levetiracetam at 1 year and 1 month resulted in shorter and less frequent seizures. At 1 year and 3 months, an increased dose of valproic acid coincided with intermittent strabismus and wide horizontal nystagmus, without concurrent epileptiform activity on EEG; nystagmus from the left eye gradually resolved. She has begun rolling onto her side, recognises relatives and shows spontaneous horizontal nystagmus (right greater than left). Dysmorphic features include low-set protruding ears, epicanthal folds, flattened nasal bridge, upturned nose, wide interdental spaces with large incisors, low posterior hairline, nipple hypertelorism, raised upper lip, partial cutaneous syndactyly of toes 2–4, and clinodactyly of the fifth fingers.

Later episodes included seizures during feeding consisting of clusters of head nods followed by generalised stiffening lasting about two minutes, often ending with a cry. Epileptic spasms with oculotonic features were observed after awakening, varying in intensity.

Family 21

Genotype: c.370A>C, p.(Lys124Gln); homozygous

Individual **F21:II.1** is a 20-year-old woman with intellectual disability, delayed speech and language development. Parents are consanguineous, originating from Iran. Pregnancy was uncomplicated and birth was at term. A transient respiratory compromise occurred immediately after birth, requiring brief resuscitation. She was noticed to have fifth finger distal phalanx clinodactyly, anteverted nares, thick eyebrow, abnormal facial shape, thin upper lip vermilion, hirsutism, low posterior and anterior hairline. Having drug dependent seizures, her EEG showed spike-wave complexes and sharp transient waves. Self-injurious behaviour was noted. CT imaging showed ventriculomegaly and enlarged interhemispheric fissure.

Her 16 years old brother **F21:II.2** was delivered at term via caesarean section. He had one seizure at age of 8 months. He has also speech delay, cannot make sentences and is dependent in daily activities such as feeding and dressing. He shares some dysmorphic features with his sister. His brain MRI report shows normal findings. Suspicion of Cornelia de Lange syndrome has been recorded in proband's medical documents.

Family 22

Genotype: c.391G>A, p.(Glu131Lys); homozygous

Individual **F22:II.1**, a 5-year-old male, is the first child of consanguineous parents from Chile. The parents are maternal first cousins (their mothers are sisters). There is no relevant family history of epilepsy.

The mother has thrombophilia, Hashimoto thyroiditis, and a heterozygous *MTHFR* mutation. A paternal aunt has systemic lupus erythematosus, and the maternal grandmother has a history of schizophrenia. The mother had a history of four previous spontaneous abortions (two first trimester abortions, one ectopic pregnancy, and one hydatidiform mole) and had a high-risk pregnancy due to comorbidities (insulin resistance, thyroiditis, and blood dyscrasia).

The patient was born at term via spontaneous delivery, small for gestational age (10th percentile), and discharged without complications.

The patient was described as normal up to 2 months of age when seizures began, characterised by upper limb cloni. Initial treatment with phenobarbital and phenytoin was ineffective. The patient developed generalised tonic seizures associated with cyanosis. Trials with vigabatrin, levetiracetam, and oxcarbazepine followed. By 4 months of age, seizures persisted and infantile spasms appeared. He received ACTH and topiramate without clinical or EEG improvement. Later, bilateral tonic-clonic seizures emerged, which treatment with valproic acid was started. At 8 months, due to ongoing infantile spasms, a ketogenic diet was started. At 15 months, with persistent spasms, oral methylprednisolone led to a partial response with decrease in spasm frequency, as well as some improvement in contact and attention. At 2 years 1 month, cannabidiol was introduced. At 2 years 4 months, ACTH and perampanel were added. Over the last 2 months, the patient has remained seizure-free, with clear EEG improvement. He is currently on vigabatrin, perampanel, cannabidiol, and levetiracetam.

The initial EEG at 2 months was consistently abnormal: slow, disorganised background activity with few physiological sleep graphoelements, multifocal interictal epileptic discharges predominantly in the posterior regions, and modified hypsarrhythmia during sleep.

The patient presents with severe delay in global psychomotor development, central hypotonia, and abnormal movements. He has difficulties to sit and hold objects and has no speech. He has no dysmorphic features and is normocephalic.

A metabolic work-up (plasma and CSF amino acids, acylcarnitines, organic acids, and pyridoxine trial) was unremarkable. Brain MRI at 3 months showed thinning of the corpus callosum.

Family 23

Genotype: c.436G>T, p.(Asp146Tyr); homozygous

F23:II.1 is a male infant of Pakistani origin born to consanguineous parents. The pregnancy was uneventful. He was well at birth but developed seizures on day two of life, requiring admission to the neonatal intensive care unit for five days. After discharge the seizures persisted, prompting readmission 10 days later. Cranial ultrasound was normal. Seizures were predominantly generalised tonic-clonic with intermittent myoclonic jerks. He was treated with phenytoin and levetiracetam.

At 3-4 months of age his events evolved into infantile spasms, and the electroencephalogram showed modified hypsarrhythmia. Prednisolone was initiated and transiently improved the spasms, but generalised tonic seizures continued. Brain MRI was unremarkable. Lacosamide was added without benefit. Repeat EEG showed similar findings; vigabatrin was prescribed with little improvement, and a second course of prednisolone was ineffective.

Development was markedly delayed. He first smiled at eight months and achieved partial head control at 12 months, followed by motor regression. He experienced recurrent chest infections. On examination he was failing to thrive; at 16 months his head circumference was 41 cm and his weight 7.7 kg. Spasticity developed in the lower limbs. He later had recurrent respiratory infections and died in hospital from pneumonia at 25 months of age.

Family 24

Genotype: c.436G>T, p.(Asp146Tyr); homozygous

Family 24 consists of two affected siblings from a consanguineous family of Pakistani descent (parents are first cousins). The pregnancy of the girl **F24:II.1** was full term. She started to have generalised tonic spasms since 1.5 months of age, with status epilepticus and clustering. EEG examination was abnormal due to burst suppression pattern. She has been administered levetiracetam, ACTH and vigabatrin. Clinical examination showed microcephaly, spasticity with cortical thumb sign, upgoing plantar response and lower limb contractures, as well as reduced muscle bulk. Other clinical features included feeding difficulties requiring tube feeding, chronic constipation and a single erythematous patch.

MRI imaging revealed diffuse brain atrophy with prominence of the extra-axial spaces and dilated ventricular spaces. She passed away at 2.2 years of age due to refractory status epilepticus.

The affected younger boy **F24:II.2** had a similar clinical picture with generalised myoclonic and tonic spasms and hypotonia since 1 month of age. His epilepsy was drug resistant and he also experienced apnoeas. On neurological examination at 2 months of age he presented with visual impairment and no gaze fixation/following or social smile. He had prominent eyes and an enlarged forehead. Other clinical features included feeding difficulties requiring tube feeding, vomiting and chronic constipation. He passed away at 6.5 months of age due to a respiratory infection leading to respiratory failure.

Family 25

Genotype: c.446A>G, p.(Tyr149Cys); homozygous

F25:II.3 is a 15-year-old female born to parents from Algeria. Family history is significant for consanguinity (parents are second cousins), and a first cousin with epilepsy. Clinical synopsis includes global developmental delay, and medically refractory epilepsy. Prenatal history was significant for maternal gestational diabetes. She was born full term via vaginal delivery. Birth was uncomplicated. Birth weight was 3.4 kg (50th centile). She was noted to have stereotypical movements (including frequent chewing, eye rolling and waving of hands) at 2-3 months of age. She was unable to sit without support at 10 months and was diagnosed with developmental delay. At about 11-12 months, she regressed (including loss of head control, loss of eye contact and tracking). Brain MRI at 11 months was significant for cortical atrophy mostly involving the anterior quadrants and opercular regions, and delayed myelination pattern pronounced in temporal lobes bilaterally. There were no obvious structural anomalies identified. EEG recorded a high amplitude disorganised background with multifocal and generalised epileptiform abnormalities that did not meet amplitude criteria for hypsarrhythmia and clinically there was concern for epileptic spasms. She was initially successfully treated with adrenocorticotropic hormone therapy (ACTH) followed by prednisone (hydrocortisone taper) and zonisamide, then vigabatrin. When she was 2 years old, she started on the ketogenic diet. EEGs at this time met amplitude criteria for hypsarrhythmia. Her seizures by this point were generalised tonic-clonic seizures. She was evaluated by genetics at 19 months: Plagiocephaly, prominent forehead, synophrys, fetal pads, premature adrenarche and a flat hyperpigmented lesion with undefined borders on thigh were noted on physical examination. Her weight was 9.5 kg (3-10th centile), height 79 cm (10-25th centile), and head circumference 45.5 cm (10-25th centile). She has had bouts of status epilepticus, once at 2 years of age and once at 10 years of age. At 15 years, she remains is non-verbal and non-ambulatory and has minimal purposeful interactions with others.

She has appendicular hypertonia and axial hypotonia, bilateral feet contractures and scoliosis. After trying several medications (levetiracetam, clobazam, phenytoin, cannabidiol) in combination with the ketogenic diet, her epilepsy has remained refractory, and she has generalised tonic-clonic seizures every 1-1.5 weeks. EEG shows moderate slowing of the background and both multifocal and generalised epileptiform discharges potentiated by sleep.

Family 26

Genotype: c.467C>A, p.(Ser156Tyr); homozygous

Family 26 includes two affected sisters born to consanguineous parents of Pakistani, Urdu-speaking origin (first cousins).

Individual **F26:II.2** (7 years) also presented with global developmental delay, more severe than in her sister. She achieved sitting at 2.5 years and remains non-ambulatory. Speech is absent, and she has severe intellectual disability. She developed generalised tonic–clonic seizures beginning at 3 days of life, which became controlled by age 3 years on valproic acid. EEG showed modified hypsarrhythmia. On examination, she has hypertonia, brisk reflexes, and spasticity. Brain MRI at 1.5 years of age was unremarkable.

Individual **F26:II.3** (4.5 years) presented with global developmental delay. She achieved unsupported sitting at 1.5 years and independent walking at 3 years. Speech development is severely delayed, with no spoken words.

She has moderate intellectual disability and is unable to perform basic activities of daily living. Neurological examination showed hypertonia and brisk deep tendon reflexes. Brain MRI performed at 3 years of age was unremarkable.

Family 27

Genotype: c.496-3_496-2del, p.Cys166ValfsTer83; homozygous

F27:II.1 is a 16-month-old male referred with a diagnosis of epileptic encephalopathy, specifically Ohtahara syndrome. The clinical history began between the age of 1–2 months with frequent seizures characterised by multiple daily myoclonic jerks and spasms. At that time, he was admitted to the hospital for two months and received steroid therapy. An EEG performed during admission demonstrated a burst-suppression pattern, and MRI of the brain revealed cerebral atrophy. He was discharged on levetiracetam and clonazepam, which he continues to receive to date. Currently, the patient experiences seizures approximately every other day. According to the mother, the episodes now present as brief tonic events lasting a few seconds, typically once per day. Developmental milestones are significantly delayed. He exhibits a positive head lag, is unable to roll over or sit independently, and is not babbling. However, according to the mother, he is able to visually follow a person.

Birth and Family History: The child was born to consanguineous parents (first cousins) via spontaneous vaginal delivery following an uncomplicated pregnancy. Birth weight was 2 kg. He cried immediately after birth and was discharged with the mother on the same day. The mother is gravida 2, para 2, with one healthy 5-year-old daughter. Family history is notable for epilepsy in a paternal cousin, who is otherwise developmentally normal. There is no history of abortion or stillbirth.

On physical examination, the patient had a head circumference of 40 cm, with an open and flat anterior fontanelle. No dysmorphic features were noted. There was no evidence of organomegaly. Neurologically, he exhibited severe truncal hypertonia with relatively preserved peripheral tone. Deep tendon reflexes were diminished (+1), and no clonus was observed.

During the examination, abnormal myoclonic movements were noted, likely representing seizure activity. Developmental assessment revealed significant global delay: positive head lag, inability to roll over or sit independently, and absence of babbling.

EEG revealed frequent multifocal sharp wave discharges, predominantly in the occipital regions bilaterally, along with continuous generalised background slowing, consistent with a severe epileptic encephalopathy pattern.

MRI of the brain demonstrated volume loss of the medulla, pons, and cerebellar hemispheres, with bulging of the left occipital area. The craniovertebral junction appeared normal. The lateral, third, and fourth ventricles were of normal size and configuration, and the corpus callosum was unremarkable. There was prominence of the extra-axial spaces in the frontal lobes and Sylvian fissures, as well as subtle left-sided temporal lobe volume loss. No abnormal white matter signal or midline shift was identified.

Family 28

Genotype: c.496-3_496-2del, p.Cys166ValfsTer83; homozygous

F28:II.1 is a 5-year-old female born at full term to a consanguineous Saudi couple via spontaneous vaginal delivery. Her birth weight was 2900 g, and Apgar scores were 8 and 9 at 1 and 5 minutes, respectively. She was admitted to the nursery immediately after birth due to dysmorphic features and developed her first seizure shortly thereafter. She remained hospitalised for one week before being discharged against medical advice. In the second week of life, she developed recurrent seizures and was admitted to the hospital again. The seizures were initially tonic in nature, later evolving to myoclonic seizures. She exhibited profound developmental delay. The initial EEG was markedly abnormal with frequent high voltage spike discharges, background of burst suppression pattern. She was started on levetiracetam and valproic acid. Metabolic workup including ammonia as well as brain MRI and CT at that time were unremarkable.

The patient was referred to the genetic service at the age of 12 months. She was having global developmental delay, hypotonia, brain atrophy, encephalopathy, laryngomalacia and recurrent aspiration.

Developmentally, she was unable to support her head, demonstrated an immature grasp, did not produce sounds, did not smile, and showed no visual fixation or tracking.

The patient had failure to thrive, with all growth parameters below the 3rd percentile: length 65.4 cm, weight 6.71 kg, and head circumference 41 cm. Facial dysmorphism included microcephaly, a hairy forehead, short philtrum, low-set ears, micrognathia, and an open mouth. She was on nasogastric tube feeding and exhibited harsh breathing sounds. Neurologic examination revealed axial hypotonia with peripheral spasticity. Orthopaedic findings included bilateral hip dislocation and scoliosis.

Metabolic investigations - including ammonia, carbohydrate-deficient transferrin (CDT), very-long-chain fatty acids (VLCFA), purine/pyrimidine panel, acylcarnitine profile, and urine organic acids -were all unremarkable. Plasma amino acids showed a mild persistent elevation in alanine, likely secondary to valproic acid therapy. She is on the following medications: valproic acid, rufinamide, topiramate, levetiracetam, and omeprazole.

Currently, she remains severely developmentally delayed, hypotonic, and suffers from intractable seizures and failure to thrive. Her most recent growth parameters continue to be below the 3rd percentile: length 80 cm, weight 9.1 kg, and head circumference 42.5 cm.

A follow-up EEG revealed generalised spike, polyspike, and wave discharges, with independent bilateral posterior epileptic activity (left > right), consistent with myoclonic epilepsy. Visual evoked potentials were significantly abnormal, showing prolonged P100 latency and widened NPN complex bilaterally, indicating optic nerve pathway pathology.

F28:II.2 is a 4-year-old female patient born at full term via spontaneous vaginal delivery. Apgar scores were 9 and 9 at 1 and 5 minutes. Birth parameters were within normal limits: weight 3290 g (50th–75th percentile), length 52 cm (90th percentile), and head circumference 32 cm (10th percentile).

Due to the family history, the patient was admitted to the nursery for observation immediately after birth. She was noticed to have dysmorphic features including a depressed nasal bridge, arched eyebrows, and a high-arched palate.

She presented with axial hypotonia and peripheral spasticity, including scissoring and bilateral severe talipes. During admission, she had recurrent episodes of desaturation, mainly during feeding. The initial ophthalmologic evaluation was normal.

All initial investigations were unremarkable including newborn screening, ammonia, urine organic acids, plasma amino acids and lactic acid. Brain MRI at one week of age was normal for the patient's age.

Shortly after discharge, she began exhibiting infantile spasms with seizure semiology similar to her sister's, primarily presenting as intractable myoclonic seizures. She remained to have axial hypotonia and peripheral spasticity. EEG showed a burst suppression pattern with focal epileptiform discharges, prompting initiation of antiepileptic therapy. Current medications: Rufinamide, Levetiracetam, Valproic acid, Clobazam, Omeprazole.

She has severe gastroesophageal reflux disease and is on nasogastric tube feeding. Growth parameters remain below the 3rd percentile: length 78 cm, weight 10.7 kg, and head circumference 43.5 cm.

Developmentally, she remains severely delayed: she is unable to support her head, has an immature grasp, does not produce vocalisations, does not smile, and does not fixate or follow visually.

Follow-up brain MRI at 15 months showed generalised cerebral volume loss and cerebellar hypoplasia.

Family 29

Genotype: c.496-3_496-2del, p.Cys166ValfsTer83; homozygous

The male child **F29:II.2** was born to consanguineous parents from Saudi Arabia, at full term by spontaneous vaginal delivery with a birth weight of 2.6 kg. The neonatal period was uneventful. At 26 days of life, he was admitted to the paediatric intensive care unit following four brief postprandial cyanotic episodes lasting a few seconds and associated with sneezing, all of which resolved spontaneously. At two months of age, he developed abnormal movements described as spastic seizures occurring after feeding, lasting less than 10 seconds without loss of consciousness. EEGs at both times were normal.

At three months, he presented with generalised spasms associated with crying. EEG demonstrated bihemispherical intermittent sharp and slow wave clusters with burst suppression lasting 1–1.5 seconds, consistent with hypsarrhythmia, and he was started on vigabatrin, prednisolone, and clonazepam.

At seven months of age, he was admitted with fever and seizures, was noted to be apnoeic and cyanosed, and required intensive care for more than two weeks. He received antibiotics and high-frequency oscillatory ventilation. EEG during this admission showed continuous bi-hemispheric lateralised periodic discharges with left posterior temporal predominance, indicating marked cortical irritability and high seizure vulnerability. The background was diffusely slow, consistent with moderate to severe encephalopathy, although no clinical seizures were recorded. Levetiracetam was added to his regimen.

At one year and nine months, he experienced recurrent intractable seizures associated with fever reaching 39 °C, cough, post-tussive vomiting occasionally blood-streaked, and repeated choking episodes. Perampanel was introduced at this stage. At two years of age, he was last reviewed in the context of a viral illness.

The family history was remarkable for consanguinity, as the parents are first-degree relatives, and for a similar neurological disorder in both a maternal nephew and a paternal cousin. His eight-year-old sister is healthy.

Growth parameters were at the fifth centile at birth but had fallen below the third centile by the age of two years (weight 7 kg, length 80 cm, head circumference 43 cm), consistent with severe growth failure. On examination, he was vitally stable and had normal results regarding chest, heart, and abdomen. Genital examination revealed bilateral palpable undescended testes and hypospadias. Neurological evaluation showed profound global developmental delay with spasticity.

Echocardiography revealed a patent foramen ovale with a normal ejection fraction of 71% and no cyanotic congenital heart disease. Brain MRI performed at two months of age was normal.

Family 30

Genotype: c.496-3_496-2del, p.Cys166ValfsTer83; homozygous

Patient **F30:II.5** is a two-year-old female who was born to consanguineous parents from Pakistan after an uneventful pregnancy. She was born at term via spontaneous vaginal delivery. Abnormal movements were noticed by her parents at the age of two days, and she was admitted to the neonatal intensive care unit at the age of one week for further investigations, where she stayed for one week. The abnormal movements lasted for about one minute and occurred four times a day, particularly upon awakening from sleep. She was diagnosed with infantile spasms and started on levetiracetam, but the seizures remained uncontrolled.

Her medications were gradually escalated. At the age of four months, she was receiving a maximised dose of levetiracetam, and phenobarbital was added with minimal effect. Vigabatrin was subsequently introduced, which helped decrease the frequency of spasms. At the age of five months, the semiology of her seizures consisted of spasms involving the upper limbs, upward eye deviation, and lip smacking. EEG at that time was suggestive of generalised epileptogenicity and evolving epilepsy consistent with Lennox–Gastaut syndrome.

Additionally, she had hypotonia, head lag and strabismus. She also developed inspiratory stridor and feeding difficulties, leading to laparoscopic-assisted gastrostomy tube insertion at age of 1.5 years. She had profound global developmental delay; at age of two years, she still did not roll over, sit or hold her head up. She was unable to hold toys, had no social interaction, and did not babble.

At two years and nine months of age, the following treatment regimen was followed: levetiracetam (60 mg/kg/day); vigabatrin (112 mg/kg/day); topiramate (7.5 mg/kg/day); clobazam (2 mg/kg/day) with the best clinical response; and valproic acid (30 mg/kg/day), which had been added just recently. Previous anti-seizure medications that were discontinued included phenobarbital and steroids due to lack of response.

On examination at the age of two years and nine months, her growth parameters were all below the third percentile. Her weight was 6.6 kg (approximately −8.7 SD), her length 81 cm (−3.0 SD), and her head circumference 43.5 cm. She had microretrognathia, epicanthal folds, a high-arched palate, and a fish-mouth appearance.

She exhibited stridor, and neurological examination revealed severe generalised hypotonia. The remainder of the general examination was unremarkable. A brain MRI was unremarkable.

Her family history was remarkable for consanguinity, as parents were first cousins, and for similar history of seizures in an older sister who passed away at the age of 8 years. She has cousins with similar issues of hypotonia, global developmental delay and seizures with positive genetic testing for the familiar CNPY3 gene variant.

Family 31

Genotype: c.548del, p.(Asn183ThrfsTer7); homozygous

Patient **F31:II.9** is a female born term with weight, length and head circumference below the 10th centile (weight -2.3SD, length -2.72SD, HC -2.36SD). Postnatal adaptation was initially uncomplicated but on the first day of life cyanotic apnoeas and stridor occurred.

In the further course, seizures became evident leading to antiepileptic medication with phenobarbital. Furthermore, oral feeding led to choking and aspirations. Failure to thrive was met with energy-supplemented feedings. Ongoing symptoms were cyanotic apnoeas treated with caffeine citrate and high-flow nasal cannula therapy, stridor due to laryngomalacia, episodes with muscular hypertonia and crying interpreted as pain, inability to swallow and retrognathia leading to tube feeding. In spite of recurrent seizures repeated EEGs did not detect any ictal or interictal epileptiform discharges. Due to worsening of clinical seizures and apnoeas, pyridoxal phosphate and levetiracetam were given. Phenobarbital and subsequently pyridoxal phosphate were suspended. A short period with slight clinical stabilisation was observed. In the sixth week of life a critical neurological worsening occurred with severe prolonged apnoeas and seizures (cloni, lip smacking, sneezing, coughing) leading to respiratory insufficiency and bradycardia requiring mechanical ventilation. Amplitude-integrated EEG did not detect any epileptiform patterns. With the suspicion of status epilepticus intravenous phenytoin was given in addition to pyridoxal phosphate, phenobarbital and levetiracetam. After 7 days the patient was extubated and treated with CPAP and HFNC. Despite thriving with tube feedings head growth was inadequate (weight -1.3 SD, length -2.5 SD, HC -5.6 SD), while swallowing problems continued. No neurodevelopmental progress could be noticed with the patient being non-communicative. Eventually a palliative care concept was established. At the end of the third month of life, cyanotic apnoeas occurred more frequently without complete recovery, eventually leading to the patient's death at an age of 3 months.

Extensive and repeated laboratory investigations including infectious and metabolic diseases did not reveal any causes or explanations for the clinical course.

Brain MRI in the fourth week of life did show signs of partial incomplete hippocampal inversion on the left, cavum septum pellucidum and slightly prominent subarachnoid spaces on the left, likely secondary to suspected atrophy in the left frontal and temporal lobes, and three periventricular punctual bleedings. Repeat brain MRI in the 11th week of life showed corpus callosum hypoplasia, progressive myelination and occipital punctual bleedings. MR spectroscopy showed lactate elevation ubiquitous in the white matter and focally in the cortex whereas N-acetylaspartate was bifrontally reduced.

Family history was highly conspicuous with consanguineous parents of Syrian origin, and in a total of eight older siblings, of which two brothers and one sister showed a similar course with epileptic encephalopathy, failure to thrive, and early demise (1st, 2nd and 3rd month of life, respectively).

Parents were advised regarding the strong suspicion of an autosomal recessive disease with a correspondent recurrence risk.

The younger sister **F31:II.10** was prenatally diagnosed as genetically affected via chorionic villus sampling. Foetal sonography revealed midline brain anomalies, with a cavum septum pellucidum and hypoplasia of the corpus callosum. A foetal MRI performed at 27 weeks of gestation confirmed these findings and showed fused, dilated lateral ventricles. Adjacent to the lateral ventricles, bilateral cystic changes in the periventricular white matter were noted, morphologically resembling those observed in her older sister.
Due to the expected extremely poor prognosis, it was agreed with the parents - following a positive vote from the ethics committee prior to delivery - that the patient would receive palliative care only. The parents declined inpatient and, in particular, intensive medical treatment, and expressed their wish for the child to be cared for at home.
The girl was born term by spontaneous vaginal delivery small for gestational age with weight, length and head circumference all below the 10th percentile (weight: -2.13SD, length: -2.59SD, head circumference: -1.9SD). Postnatal adaptation was generally adequate, with spontaneous breathing. However, she was cyanotic until the 8th minute of life, accompanied by grunting and nasal flaring. APGAR scores were 8/8/8.
Following birth, the patient presented with growth retardation, suspected seizures (lip smacking), laryngomalacia, and impaired swallowing. From the second week of life, she was cared for by our specialised paediatric palliative care team. Management focused primarily on feeding difficulties and recurrent vomiting, which persisted despite intermittent nasogastric tube feeding. High-calorie fortified nutrition was not tolerated, and adequate weight gain was not achieved.

From the third month of life, she began experiencing recurrent seizures of varying morphology, including BNS-like seizures and tonic episodes (axial and fencing postures), often accompanied by deep cyanosis. Despite trials of vigabatrin, valproic acid, midazolam, and phenobarbital, seizure control remained inadequate, with persistent daily seizures.

Over time, the patient exhibited decreasing wakefulness, continued vomiting, constipation, and complete absence of developmental progress. At 3.5 months of age, EEG showed diffuse slowing, frequent bilateral poly-spikes and poly-spike-waves. Brain MRI showed cerebral atrophy with irregularly shaped lateral ventricles. The sedation performed for this purpose with midazolam and chloralhydrate led to a temporary but severe impairment of vital functions, which eventually resolved spontaneously.

In the further course, epileptic encephalopathy with no neurological development was observed leading to a most severe neurodevelopmental impairment. Initial generalised muscular hypotonia evolved into marked spasticity toward the end of the first year of life, which was initially intermittent but became persistent over time.

Additionally, extreme growth failure occurred. At 24 months of age, weight, length, and head circumference were all far below the 1st percentile (-7.51 SD, -6.11 SD, and -10.95 SD, respectively). The child passed away at the age of 30 months - significantly later than her previously affected siblings.

Family 32

Genotype: c.548del, p.(Asn183ThrfsTer7); homozygous

F32:II.1 was a female infant born at 39+0 weeks of gestation by primary caesarean section due to breech presentation. Birth measurements were within normal limits: weight at the 48th percentile, length at the 81st percentile, and head circumference at the 47th percentile. Postnatally, the patient required hospitalisation for respiratory adaptation disorder and significant orofacial dysfunction leading to poor feeding. She was discharged after 23 days on bottle feeds, with a body weight 100 grams below birth weight.

At 4 months and 3 days of age, she was admitted for failure to thrive with weight and head circumference both below the 1st percentile and length at the 3rd percentile. Due to lack of responsiveness, absence of fixation, and generalised muscular hypotonia, an EEG was performed, which revealed a hypsarrhythmia pattern. The parents retrospectively reported seizure episodes consistent with BNS type. Therapy was initiated according to guidelines with prednisolone and vigabatrin. After 7 days, the patient was seizure-free, and follow-up EEG on day 14 showed resolution of hypsarrhythmia. However, the following day, seizures recurred, prompting the addition of levetiracetam.

A concurrent respiratory infection was diagnosed with detection of enteroviruses and rhinoviruses. One week later, new clusters of BNS-like seizures occurred. Valproate was added, and prednisolone was tapered following the ICISS study protocol. After initial clinical stabilization, seizure frequency worsened again approximately two weeks later. EEG showed increasing epileptiform activity with a burst-suppression-like pattern.

Around 6 weeks after initial admission, the patient developed recurrent apnoeas with significant desaturations. Treatment with benzodiazepines and phenobarbital was initiated in case of a convulsive aetiology. However, neither aEEG nor standard EEG showed epileptiform activity during the apnoeas, which were therefore considered non-epileptic. Supportive therapy with flumazenil, caffeine citrate, doxapram, and high-flow nasal cannula (HFNC) provided only minimal passive stabilisation. Despite escalation of anticonvulsant therapy - adding phenobarbital, lacosamide, and topiramate - there was no improvement in seizure control or apnoea frequency.

The patient was predominantly tube fed with a high-calorie diet but failed to achieve adequate weight gain. She remained unresponsive to her environment and showed no signs of motor development.

Brain MRI revealed prominent cerebrospinal fluid spaces indicating white matter deficiency, as well as severe myelination delay consistent with a neonatal stage. Extended diagnostic workup, including metabolic screening, neuro-ophthalmological evaluation, and CSF analysis for glucose transporter deficiency, yielded no pathological findings.

The patient was born to consanguineous Syrian parents; the child's father is the son of the maternal grandfather's sister. One child in the extended family (uncle's child) was also reported to have severe developmental delay.

After withdrawal of anticonvulsant therapy, seizures did not increase, but the apnoeas with profound desaturations progressed. The patient was transferred to a hospice setting for palliative care and died at the age of 7 months.

Family 33

Genotype: c.707_716del, p.(Ala236Glyfs*49); homozygous

F33:II.3 is a 3-year-old female patient, the third child of consanguineous parents (first-degree cousins) of Egyptian origin. The family history was unremarkable. The perinatal history was uneventful, and she was born at 38 weeks of gestation via normal spontaneous vaginal delivery.

Seizure onset occurred at 1 month of age, with various seizure types. Initially, the patient experienced daily focal myoclonic seizures, which significantly decreased in frequency after the initiation of levetiracetam and clonazepam. The last reported episode occurred at 1 year of age. Epileptic spasms, primarily of the extensor type, occurred in daily clusters and were controlled with the addition of valproic acid. The last episode of spasms occurred at 8 months, at which point they evolved into generalised tonic seizures. These generalised tonic seizures were characterised by tonic posturing, staring, and drooling. They occurred daily - up to 10 times per day - and were resistant to multiple antiseizure medications. However, seizure frequency was reduced by more than 50% following the initiation of a ketogenic diet. At the time of last follow-up, the patient was experiencing 2-3 seizures per month. There is also a history of status epilepticus, with no identifiable triggers.

An EEG at 3 months showed a suppression-burst pattern, with two myoclonic seizures captured and electrographically correlated to generalised polyspike wave complexes, followed by an electrodecremental response lasting 1 second. By 6 months of age, the EEG had evolved to show a modified hypsarrhythmia pattern. A brain MRI conducted at 10 months of age was reported as normal.

The patient had a profound developmental delay. At 3 years of age, she was non-ambulatory, had no head control, and was non-verbal, although she vocalised nonspecific sounds. Visual impairment was present, though she had gained the ability to fixate and follow objects. Spasticity and lower limb contractures were managed with baclofen. She developed secondary microcephaly, first noted at 3 months of age, along with significant failure to thrive and swallowing difficulties, necessitating gastrostomy tube placement.

The patient died at 53 months of age without a clear precipitating event; sudden unexpected death in epilepsy (SUDEP) was considered a possible cause.

Family 34

Genotype: c.733_736del, p.Gln245GlyfsTer42

F34:II.5 is a 4-month-old male, born to consanguineous parents of Sudanese origin. He has three healthy siblings, one sister and two brothers. Another sister (F34:II.4) was born with alobar holoprosencephaly and a congenital cardiac malformation and died within the first hour of life. Genetic investigations in that case (karyotype, chromosomal microarray, and trio-based exome analysis) were unremarkable. There is no other family history of malformations or premature death.

F34:II.5 was born at 37 weeks of gestation from a pregnancy complicated by maternal diabetes and intrauterine growth restriction.

His early neonatal period was marked by feeding difficulties, episodes of loss of consciousness, and generalised hypertonia affecting both upper and lower limbs. He soon developed abnormal movements, including frequent episodes of opisthotonus. He experienced multiple episodes of cardiorespiratory arrest with successful resuscitation. Brain MRI and MR spectroscopy were nonspecific. EEG recordings were abnormal, showing hypsarrhythmia and a burst-suppression pattern. Clinically, he showed no developmental progress and presented with strabismus. Based on this constellation of findings, a diagnosis of epileptic encephalopathy was made.

He is now 4-month-old. Given gastroesophageal reflux, laryngomalacia (associated with stridor) and inappropriate weight gain, he is fed exclusively via gastrostomy. Seizures are currently well controlled with a combination of vigabatrin and levetiracetam, after being exacerbated by carbamazepine.

He presents with congenital microcephaly (-2.1 SD) and failure to thrive, with weight and length both significantly below the norm (-3.5 and -3.7 SD, respectively). Dysmorphic features include a high forehead, triangular face, strabismus, and posteriorly rotated ears. Extensive metabolic workup, including blood, urine, and cerebrospinal fluid analysis, yielded normal results.

He exhibits minimal interaction with his surroundings and does not track visually. However, he can remain calm when held by his mother. Given his relative clinical stability, absence of further syncopal episodes, and in agreement with the family's wishes, he has been discharged home under paediatric palliative care. The parents have been counselled on the poor prognosis and have agreed with the collective decision of comfort care.

Family 35

Genotype: c.734_737del, p.(Gln245ArgfsTer42); homozygous

The male patient (**F35:II.2**), a 1.5-year-old child of Pakistani descent, was born to healthy consanguineous parents. He was the second child in the family and had one unaffected older brother. There was no family history of seizures or other neurological disorders.

The patient was delivered via caesarean section due to previous scar with a birth weight of 2.6 kg. The neonatal course was otherwise unremarkable. At 3 months of age, concerns were raised regarding abnormal body movements, prompting neurological evaluation at 4 months. At that time, the patient was able to control his head.

Over the following five months, the patient developed frequent seizure episodes, including generalised tonic-clonic and myoclonic seizures, occurring approximately 7–8 times per day, sometimes with cyanotic apnoeas. He also experienced recurrent episodes of gastroesophageal reflux and feeding difficulties, necessitating nasogastric tube feeding. Developmental regression was noted during this period, with loss of previously achieved head control.

MRI of the brain, performed at 8 months of age, revealed no structural abnormalities. However, EEG at the same age demonstrated a pattern consistent with hypsarrhythmia, characterised by a disorganised background and frequent high-voltage spike-and-wave discharges, most prominent in the bilateral parieto-occipital regions. These discharges were variably generalised or multifocal in distribution.

Seizure control was not achieved despite multiple antiepileptic drugs, including phenobarbital, levetiracetam, and clobazam. The patient exhibited profound global developmental delay. His clinical course was further complicated by multiple hospitalisations for recurrent pneumonias.

At 16 months of age, he contracted a methicillin-resistant Staphylococcus aureus infection during hospitalisation, which ultimately led to death due to infectious complications.

Family 36

Genotype: c.825del, p.(Asp276MetfsTer12); homozygous

F36:II.6 is a 5-year-old boy, the sixth child of healthy consanguineous parents of Pakistani descent (parents are first cousins). He was born full term via spontaneous vaginal delivery. He presented at 6 months of age with generalised tonic-clonic seizures, which later developed to myoclonic jerks at 8 months of age. This was accompanied by neurodevelopmental delay, severe intellectual disability, failure to thrive and severe loss of achieved milestones, such as neck holding. EEG revealed runs of high-voltage multifocal spike-and-wave discharges, poorly spreading with a generalised bursts of high-voltage spike-and-wave complexes followed by periods of suppression. He was treated with valproic acid and levetiracetam. Clobazam was trialled but had to be discontinued due to extrapyramidal side effects and insomnia. Brain MRI carried out at 5 years of age showed T2-weighted hyperintensities in the peritrigonal region, periventricular white matter, and centrum semiovale. Additional clinical features include constipation, feeding difficulties, and recurrent infections, predominantly respiratory.

He has four deceased siblings due to severe seizures and status epilepticus.

Family 37

Genotype: c.832C>A, p.(Leu278Ile); homozygous

F37:II.1 is a 4-year-old girl, the first child of healthy consanguineous parents of Syrian descent (parents are first cousins). She has a healthy younger brother. The birth of the girl was at full term. She started to have focal tonic seizures since 3 months of age, at around 6 months of age she developed myoclonic spells, which were followed by tonic-clonic and continued myoclonic seizures. She has been administered levetiracetam at first with improvement, then valproic acid was added with mild improvement. However, it had to be discontinued due to severe thrombocytopenia. Topiramate was then introduced, leading to mild improvement in seizure control. Clinical examination showed severe global developmental delay, generalised weakness, hypotonia, and bilateral lower limb spasticity. She barely sits with support. EEG showed generalised slowing and right-sided focal spikes. She presents with failure to thrive, absent speech and severe intellectual disability. Other clinical features included feeding difficulties, episodes of vomiting and regurgitation, constipation, and recurrent severe infections. She has a history of very frequent hospitalisations due to aspiration pneumonia, bronchitis and recurrent otitis media. She also presents with nystagmus and exophthalmos. Brain MRI performed at 2 years of age was unremarkable.

Family 38

Genotype: c.832C>A, p.(Leu278Ile); homozygous

Family F38 consisted of two affected siblings, a female (F38:II.1) and a male (F38:II.2), aged 10 and 6 years, respectively. They were the only children of healthy first-cousin parents from Upper Egypt. Both pregnancies and perinatal periods were uneventful.

The female patient **F38:II.1** was first evaluated at 6.5 years of age. She showed severe global developmental delay with absence of motor milestones, spastic quadriparesis, and refractory epilepsy. Myoclonic seizures began at 37 days of age, occurring in clusters, and remained uncontrolled thereafter. She also exhibited severe irritability, sleep disturbance, and repetitive behaviours.

Seizure frequency ranged from 4 to 7 episodes per day, predominantly myoclonic spasms, with occasional generalised tonic–clonic seizures and cyanotic tonic episodes. Despite multiple antiepileptic regimens—including valproate, levetiracetam, topiramate, vigabatrin, oxcarbazepine, and clonazepam—seizure control was never achieved.

At her last clinical evaluation (10 years of age), she remained encephalopathic, irritable, and nonverbal, with absent purposeful responses. She showed visual tracking and turned toward sounds but had no expressive language. Neurological examination revealed severe spastic quadriparesis with brisk tendon reflexes.

Anthropometric assessment demonstrated failure to thrive: weight of 18 kg (-3.1SD), length of 122 cm (-2.4SD), and head circumference of 48.5 cm (-3.3SD). A gastric feeding tube was inserted at age 8 years, but her weight did not improve.

Investigations revealed normal karyotype, normal metabolic workup, and unremarkable ophthalmologic and electrophysiological studies (fundus, ABR, VEP, ERG). EEG showed a markedly abnormal background with generalised spike-wave discharges. Cranial CT at 40 days was normal; brain MRI at 2 and 4 years demonstrated mild thinning of the corpus callosum and minimal periventricular deep white matter signal changes.

She died at 11 years of age due to high fever, chest infection, and status epilepticus.

The younger male sibling **F38:II.2** showed a milder clinical course. He achieved head control at 2 years, independent sitting at 3 years, and was able to stand and walk with support by 6 years. Myoclonic seizures began at 2 years of age and were moderately controlled with levetiracetam and valproate by age 4.

He exhibited hyperactivity, sleep disturbance, and less irritability compared to his sister. At the most recent evaluation (6 years of age), he demonstrated autistic features, responded to sound, produced infrequent single-syllable words, but could not use his hands purposefully. He also had episodes of loud vocalisations.

Anthropometric parameters were as follows: 17 kg for weight (-1.6SD), 110 cm for height (-1SD), and 49 cm for head circumference (-1.8SD). Neurological examination revealed hypertonia of both upper and lower limbs with brisk reflexes and spontaneous clonus. EEG showed bilateral temporoparietal spike–slow wave discharges. Brain MRI at 2 years of age was near normal, revealing only mild deep white matter signal abnormalities and mild thinning of the corpus callosum.

Supplementary Methods: Phenotype Severity Score — Detailed Scoring Manual

Overview

The phenotype severity score (PSS) is a domain-based composite score designed to quantify clinical severity across seven domains: development/cognition, motor function, speech, epilepsy, EEG, growth, and respiratory involvement. Each domain is scored from 0 to 3, with higher scores indicating greater severity. The total score ranges from 0 to 21.

Scoring was based on retrospectively collected HPO-coded clinical data and applied using predefined rules to ensure consistency and reproducibility.

Rationale for score design

The PSS was designed to balance clinical interpretability, robustness to missing data, and applicability across a wide age range. The domain-based structure captures key dimensions of disease severity while enabling aggregation into a single continuous measure.

Domains were selected to reflect major aspects of disease burden that were consistently documented across the cohort, including neurodevelopment, motor function, epilepsy, and respiratory involvement, complemented by objective measures such as EEG and growth.

Microcephaly, although a common feature, was not included, as z scores could not be reliably calculated in approximately half of cases due to missing data or lack of appropriate reference standards.

Speech was retained as a domain despite its non-continuous nature, as the presence or absence of functional speech was considered a clinically relevant discriminator, particularly in individuals with severe motor impairment.

Premature death, although clinically relevant, was not included, as it represents an outcome event rather than a cross-sectional phenotypic characteristic and may be influenced by follow-up duration and survival bias.

General scoring rules

- Scoring was based on the most severe documented phenotype per domain.
- If multiple data points were available, the highest severity category was assigned.
- Domains were scored independently.
- Missing data were recorded as missing and were not imputed.
- If partial data were available for a domain, scoring was based on the available information.
- All rules were defined prior to genotype–phenotype analyses.

Domain definitions and scoring criteria

Domain	Variables	Rules	Score 0	Score 1	Score 2	Score 3
Develop-ment / cognition	GDD (<5y), ID (≥5y), developmental regression	Use GDD <5y, ID ≥5y; if both available use more severe; regression overrides	Normal development	Mild or moderate delay or ID	Severe delay or ID	Profound impairment or any developmental regression
Motor function	Best motor milestone achieved, presence of spasticity	Age-adjusted; based on deviation from expected milestones; spasticity contributes to grading	Age-appropriate	Mild delay	Moderate impairment	Severe impairment (minimal or no milestone acquisition)
Speech	Presence or absence of functional speech	Binary classification due to limited data granularity	Functional speech present	n/a	n/a	Absent or non-functional speech
Epilepsy	Presence of epilepsy, drug response, status epilepticus (SE)	Seizure type not used; drug resistance/ SE define highest severity	No epilepsy	Controlled epilepsy	Active epilepsy without clear drug resistance	Drug-resistant epilepsy and/or SE
EEG	EEG findings	Use most severe recorded abnormality	Normal EEG	Nonspecific abnormalities (e.g. background slowing)	Epileptiform activity	Severe ence-phalopathy patterns (e.g. hypsarrhyth-mia or burst-suppression)
Growth	Weight z score, presence of failure to thrive/growth faltering	z score primary; if unavailable, FTT used; assign highest severity if both available	$z > -2$	$-3 < z \leq -2$	$-4 < z \leq -3$	$z \leq -4$
Respira-tory involve-ment	Apnoeas or cyanotic episodes, stridor, laryngo-malacia	Apnoea or cyanosis define max. severity; otherwise based on combination of structural abnormalities	No abnormalities	Stridor or laryngomalacia	Stridor and laryngomalacia	Apneas or cyanotic episodes

GDD, global developmental delay. ID, intellectual disability.

Interrater reliability and consensus scoring

All patients were independently scored by two clinical raters using the above rules. Interrater agreement was assessed using Cohen's kappa (or weighted kappa) for individual domains and the intraclass correlation coefficient (ICC) for total scores. Discrepancies were resolved by consensus review, and consensus scores were used for all subsequent analyses.

Calculation of total and normalised scores

The raw PSS was calculated as the sum of all available domain scores:

$$PSS_{raw} = \sum D_i$$

To account for missing data, a normalised score was calculated:

$$PSS_{norm} = \frac{PSS_{raw}}{3 \times (\text{number of observed domains})} \times 21$$

This approach preserves comparability across individuals with incomplete data while maintaining the full 0–21 scale. The normalised score (PSS_norm) was subsequently reported as PSS throughout the manuscript. For display purposes, individual total PSS values are shown as rounded integers to facilitate interpretation.

Robustness of the PSS

The PSS was applied to datasets with varying degrees of completeness; normalisation allows comparability between cases with and without missing data. Independent scoring by two raters, followed by consensus resolution, reduced subjectivity and supported reproducibility. The relationship between PSS values and phenotypic clustering was explored to assess whether the score reflects variation in clinical severity.
