# Supplementary material for: CNPY3 variants associated with a recessive developmental and epileptic encephalopathy affect inflammasome and TLR function
Source: eBioMedicine. 2026 Oct 14;133:106506. doi: 10.1016/j.ebiom.2026.106506 (PMC13694807; doi:10.1016/j.ebiom.2026.106506)
Supplement: Supplementary file 2 [file mmc3.docx]

## **Supplementary Data 2: Rates of Missing Data for Clinical Variables**

Calculated from the detailed clinical data in Supplementary Data 1.

| **Missing rate** | **Variables** |
| --- | --- |
| 0% | Country of origin, Consanguinity, Sex, Age at clinical presentation, Age at last examination, Global developmental delay, Intellectual disability, Epilepsy, Seizures, Decreased immune function |
| <5% | Absent speech, Encephalopathy, Status epilepticus, Failure to thrive, Feeding difficulties, Recurrent infections, Stridor, Number of antiseizure medications |
| 5–9% | Microcephaly, Spasticity, EEG findings, Current medication |
| 10–19% | Developmental regression, Intractable epilepsy, Apneas/Cyanotic episodes, Laryngomalacia, Chronic constipation |
| 20–39% | Weight/head circumference at last examination, Birth weight, Visual impairment |
| 40–50% | Length at last examination, Birth length, Birth head circumference, Hearing impairment |
