# Supplementary material for: CNPY3 variants associated with a recessive developmental and epileptic encephalopathy affect inflammasome and TLR function
Source: eBioMedicine. 2026 Oct 14;133:106506. doi: 10.1016/j.ebiom.2026.106506 (PMC13694807; doi:10.1016/j.ebiom.2026.106506)
Supplement: Supplementary file 3 [file mmc7.docx]

## **Supplementary Data 6: *CNPY3* variants analysed in this study**

The table lists the investigated *CNPY3* variants, the corresponding cell lines, and the zygosity of each variant. Italicized variants were only analysed for expression.

| **DNA change** | **Protein change^a^** | **Cell line designation** | **Zygosity** |
| --- | --- | --- | --- |
| *c.275C>T* | *S92L* | CNPY3^S92L^ | hom |
| c.292G>A | E98K | CNPY3^E98K^ | hom |
| c.329A>G | Y110C | CNPY3^Y110C^ | hom |
| c.362G>A | R121Q | CNPY3^R121Q^ | hom, het |
| *c.373G>C* | *G125R* | CNPY3^G125R^ | het |
| c.467C>A | S156Y | CNPY3^S156Y^ | hom |
| *c.548del* | *N183X* | CNPY3^N183X^ | hom |
| c.628C>T | Q210* | CNPY3^Q210*^ | het |
| c.362G>A; c.628C>T | R121Q; Q210* | CNPY3^R121Q^; CNPY3^Q210*^ | comp-het |

comp-het, compound-heterozygous; het, heterozygous; hom, homozygous

^a^Protein changes are given using the IUPAC one-letter amino acid code.
